# Optimizing a Massive Parallel Sequencing Workflow for Quantitative miRNA Expression Analysis

**DOI:** 10.1371/journal.pone.0031630

**Published:** 2012-02-20

**Authors:** Francesca Cordero, Marco Beccuti, Maddalena Arigoni, Susanna Donatelli, Raffaele A. Calogero

**Affiliations:** 1 Department of Computer Sciences, University di Torino, Torino, Italy; 2 Molecular Biotechnology Center, University of Torino, Torino, Italy; University of Rome, Italy

## Abstract

**Background:**

Massive Parallel Sequencing methods (MPS) can extend and improve the knowledge obtained by conventional microarray technology, both for mRNAs and short non-coding RNAs, e.g. miRNAs. The processing methods used to extract and interpret the information are an important aspect of dealing with the vast amounts of data generated from short read sequencing. Although the number of computational tools for MPS data analysis is constantly growing, their strengths and weaknesses as part of a complex analytical pipe-line have not yet been well investigated.

**Primary findings:**

A benchmark MPS miRNA dataset, resembling a situation in which miRNAs are spiked in biological replication experiments was assembled by merging a publicly available MPS spike-in miRNAs data set with MPS data derived from healthy donor peripheral blood mononuclear cells. Using this data set we observed that short reads counts estimation is strongly under estimated in case of duplicates miRNAs, if whole genome is used as reference. Furthermore, the sensitivity of miRNAs detection is strongly dependent by the primary tool used in the analysis. Within the six aligners tested, specifically devoted to miRNA detection, SHRiMP and MicroRazerS show the highest sensitivity. Differential expression estimation is quite efficient. Within the five tools investigated, two of them (DESseq, baySeq) show a very good specificity and sensitivity in the detection of differential expression.

**Conclusions:**

The results provided by our analysis allow the definition of a clear and simple analytical optimized workflow for miRNAs digital quantitative analysis.

## Introduction

The fine detail provided by sequencing-based transcriptome surveys suggests that RNA-seq is likely to become the platform of choice for interrogating steady state RNA. Massive Parallel Sequencing methods (MPS) can extend and improve the knowledge obtained by conventional microarray technology both for mRNAs and non-coding RNAs, e.g. miRNAs. It has been described that, in the area of miRNAs, Locked Nucleotide based Arrays (LNA) show a detection performance comparable to that of MPS technology [1]. However, MPS has the advantage that data does not rely on a specific annotation release as in the case of microarrays and quantitative real-time RT PCR (qPCR). Therefore, any time a new release of the genome or miRNA database [2] appears it is possible to map again MPS data, thus gaining new knowledge on the basis of the updated annotations. Last but not least MPS can facilitate the discovery of new miRNAs.

**Figure 1:**
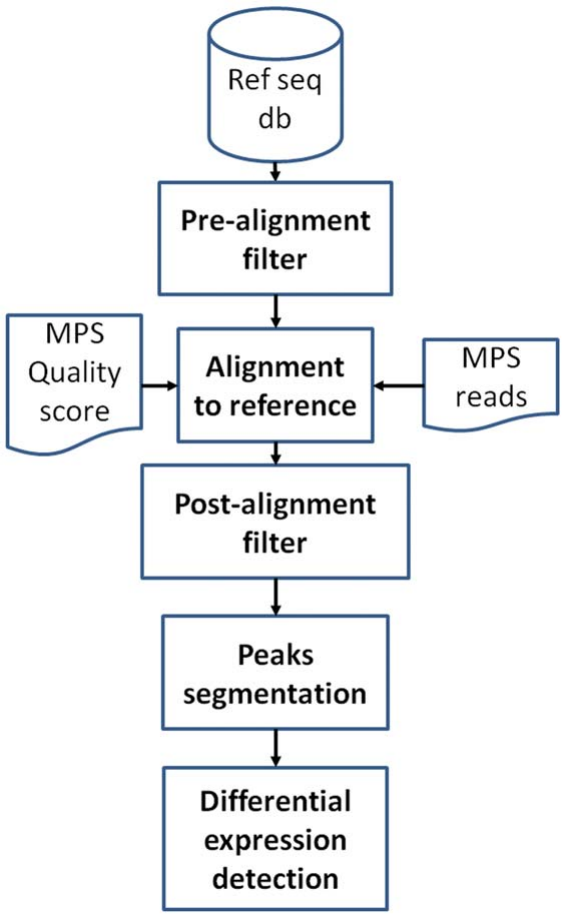
MPS workflow. Ref seq db is the reference sequence used to align reads, e.g. whole genome, miRBase. Pre-alignment filter refers to filters used to trim 5′ and 3′ linkers. Alignment to reference refers to the step in which a specific algorithm is used to align each of the reads to the reference sequence. This alignment can be done with/without considering the quality score associated with each base. Post-alignment filters are those used to remove low quality reads, alignments characterized by sequencing errors or multiple mismatches. Peaks segmentation refers to the definition of genomic regions characterized by enrichment of reads mapping, i.e. clusters of reads. Differential expression detection is the part of the analysis in which digital data are used to identify differentially expressed genes. Each of the workflow steps can be done using a variety of bioinformatics and statistical tools.

An important aspect of dealing with the vast amounts of data generated from short reads sequencing is the processing methods used to extract and interpret the information. A bottleneck in data analysis is given by the mapping, counting and characterization of the short sequence reads produced by massive parallel sequencing technologies. Although the number of computational tools for MPS data analysis is constantly growing, their strengths and weaknesses as part of a complete analytical pipe-line have not yet been well investigated. The steps involved in quantitative differential expression analysis of miRNAs are highlighted in Figure 1. The steps shown in Figure 1 can be performed using various bioinformatics/statistical tools.

In this paper, we have compared, for each step of the workflow (Figure 1), the efficacy of different tools in defining the optimal set of methods which will maximize the analytical power of the MPS workflow. Finally we suggest an optimized workflow for the quantitative detection of differential expression for miRNA digital data.

## Results

### Benchmark dataset

To evaluate the performance of tools used to map and quantify MPS data a benchmark data set of short reads, possibly characterized by spikes-in of known miRNAs amounts with multiple experimental replications, was needed. Such a data set was deposited on GEO (www.ncbi.nih.gov/geo), as GSE14511 series, by the Willenbrock group [1] and represents a tremendous instrument for building up a benchmark dataset.

Willenbrock and co-workers, as part of their paper that compares the efficacy of MPS and LNA microarrays for the quantification of miRNAs, released four barcoded sets of reads for two experiments: A and B. These experiments were generated using a total of 744 human mature miRNA spiked-in at different concentrations (additional information S1). The barcoded libraries for A and B were produced by four independent cDNA syntheses, named 1 to 4, tagged by a different barcode, i.e. short oligonucleotide sequence, inserted in one of the adaptors used to produce the cDNA library A1 to A4 and B1 to B4. The barcoded libraries were sequenced directly, without mixing them with a common complex background, e.g. cell line total RNA. To make the Willenbrock barcoded reads set more similar to a real data set we mixed them with four runs of short reads from healthy donor peripheral blood mononuclear cells, BG1 to BG4. This approach resulted in the production of a set of reads, which resembled a situation in which miRNAs are spiked in a paired biological replication experiment (Table 1).

**Table 1:** Spike-in experiment.

	Experiment A(10^6^ reads)				Experiment B(10^6^ reads)			
Sample Name	A1BG1	A2BG2	A3BG3	A4BG4	B1BG1	B2BG2	B3BG3	B4BG4
**Spike-in**	1.2	1.0	1.6	1.4	1.0	0.7	1.2	9.2
**PMBC** **(backgroud)**	5.8	11.0	8.4	7.0	5.8	11.0	8.4	7.0
**Total**	7.0	12.0	10.0	8.4	6.8	11.7	9.6	16.2

It has been recently highlighted that results produced with Illumina technology can be affected by many variables, e.g. library preparation protocol [3], barcoding [4], local sequence composition [5], etc. Also, Willenbrock [1] highlighted the presence of bias affecting miRNA quantification upon multiplexing, due probably to individual barcode differential ligation and amplification efficiencies. In our experiment setting, we tried to moderate as much as possible these effects. To moderate library preparation, the background dataset was generated using the same procedure used by Willenbrock group and libraries were run on the same type of Illumina sequencer. Concerning the barcoding bias affecting the Willenbrock barcoded data, we could not incorporate it in our background data, since background data were generated without barcoding. However, since Willenbrock barcoded data provides simply a set of true positive differentially expressed miRNAs, the presence of replicated data characterized by high sample to sample variability will simply increase the dataset variability, making the true positive set more similar to a biological replication instead of a technical replication.

### Defining the optimal reference sequence set for alignments

The first step in the analysis workflow is the alignment of short reads to a reference set of sequences. Mapping reads over the whole unmasked genome (*wg-set* for short; ftp://ftp.ncbi.nih.gov/genomes/) represents an unbiased option, allowing the detection of known and still undiscovered miRNAs. Mapping reads against the mirBase [2] miRNA precursor (*mir-set* for short, http://www.mirbase.org/) is a more conservative view, which resembles the situation observed in miRNA microarray analysis, where the analysis is focused only on the hybridization on miRNA specific microarray and not on a whole transcriptome array. The first has the weakness that it might favor alignment ambiguities due to the limited alignment specificity given by the small length of mature miRNAs (18–25 nts), detected by the short reads, and to the size and high complexity of an unmasked reference genome. The latter option is limited since it does not allow the identification of uncharacterized miRNAs. To evaluate which of these two reference sequences gave the best results in the view of quantifying digital data, we mapped the Willenbrock barcoded experiment, i.e. the set without PBMC background addition, against wg-set and *mir-set* using the SHRIMP mapping tool [6]. We also applied a post-alignment filter retaining only perfect matches and matches with one SNP (Single Nucleotide Polymorphism).

To simplify the interpretation of the above described analysis we decided to not take into account the entire set of mature miRNA isoforms available in the Willenbrock spike-in experiments, but we reorganized the Willenbrock spike-in set to have nominal spike-in concentrations recalculated at miRNA precursor-level. We considered only the subset of miRNAs which could be associated with a unique miRNA ENSEMBL gene identifier. This reorganization was required to minimize inconsistencies at mapping level, thus avoiding the counting of short reads directly on different mature miRNA isoforms [7] and counting miRNAs present in clusters and therefore characterized by multiple locations of the same sequence in the ENSEMBL genome annotation. This reorganization resulted in a total of 427 miRNA genes (benchmark set BS; additional information S2).

The use of the *wg-set* as a reference sequence allowed the mapping of about 9% more short reads compared to the *mir-set*. However, the use of the *wg-set* resulted in an increment of short reads removal by the post-processing filter, due to the presence of a higher number of reads with multiple SNPs. The intersection of the miRNAs detected using the *wg-set* and *mir-set* showed that both reference sets had nearly the same ability in detecting miRNAs which were part of the BS benchmark set: 404 miRNAs (94.6%) were detected by using both the *wg-set* and mir-set as reference. 8 miRNAs (1.9%) were only detected by *wg-set* and 6 (1.4%) only by the *mir-set*.

**Figure 2:**
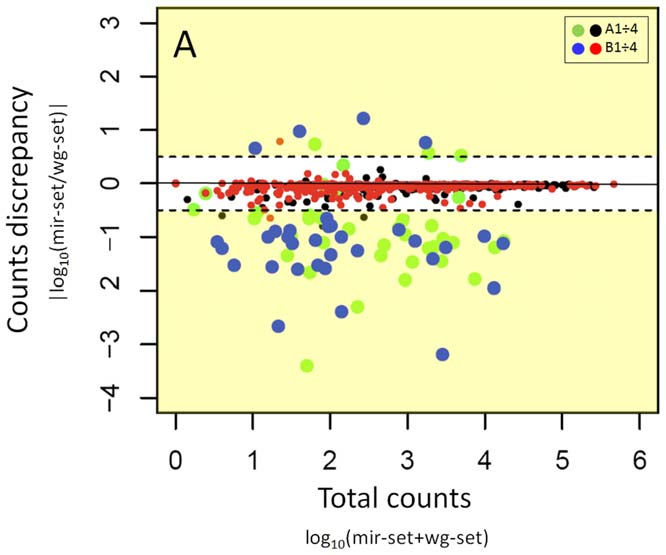
Discrepancy in short reads counts detection using whole genome (*wg-set*) and miRNA precursor set (*mir-set*) as reference. We expect that, if reference is not playing any specific role in the alignment procedure, then the same number of counts should be detected independently from the reference set in use. A higher number of miRNAs are shown to be underestimated when the wg-set is used as reference for the mapping (36 miRNAs) with respect to the *mir-set* (4 miRNAs). Red and black dots refer respectively to miRNAs detected in experiment A and B without significant variation between *mir-set* and *wg-set*. Green and blue dots refer respectively to miRNAs detected in experiment A and B with significant variation between *mir-set* and *wg-set*.

Interestingly, when inspecting the total counts detected for each miRNA using the two reference sets, the presence of 9 times more miRNAs, characterized by average fold change underestimation of counts, was notable when the wg-set is used, compared to the *mir-set* (Figure 2, green and blue dots). We further investigated this issue to understand the reason of such differences in counting upon the use of different reference set. We observed that the underestimation was mainly due to erroneous mapping of the reads on the *wg-set*. Table 2 summarized the data referring to four out of 36 under estimated miRNAs for the *wg-set* and all four underestimated for *mir-set*. The above mentioned erroneous reads assignment is due to the possibility of finding by chance an alignment of a mature miRNA sequence over a large genome such as the human, e.g. miRNA targets sites located in the 3′ end of genes. These erroneous associations could be removed applying an annotation-based post-alignment filter, e.g. filtering out all reads that do not overlap to ENSEMBL miRNAs annotation. Such filter can be easily implemented, e.g. using the functionalities present in the GenomicRanges Bioconductor package. Unfortunately this approach cannot fix the above mentioned under-estimation issue, which could be instead moderated by applying post alignment procedures focusing on specific characteristics of miRNA structure, e.g. miRDeep [8] investigates the secondary structure of each potential precursor as well as the positions of the reads that align to it. Another option could be to align reads against a genome sequence where pseudo-miRNA mature sequences, not inserted in a correct miRNA precursor sequence content, are masked. However, we could not find any published post-alignment tool able to handle both erroneous mapping assignment and reads counting as well as any genomic masking tool that could be easily adapted to the above mentioned masking procedure. Therefore, since the use of miRBase as reference is less affected by erroneous mapping, we suggest using it as a reference at least at the present time.

**Table 2:** Discrepancies in mapping between *mir-set* and *wg-set*.

Experiment A1(reads)												
*wg-set* under estimation					mir-set under estimation							
miRNA	378	202	548c	151	517	218	16	509				
	-	-	-	-	a	b	1	2	1	2	1	3
**mir-set**	767	827	149	1395	242	1926	60	1	30	31	85	290
**wg-set** **(common with mir-set)**	277	79	0	29	242	-	-	1	30	-	85	-
**mir-set only**	490	744	149	1366	1926	-	-	61	31	-	292	-
**wg-set total**	767	823	149	1395	2168	-	-	62	61	-	377	-
**% of common assignment**	35	9.5	0	2.0	11.1	-	-	0.1	49.1	-	22.5	-

In case of under estimation in the *wg-set*, we observed that all reads detected as associated with a specific miRNA on the *mir-set* are only partially associated with one location, the others are scattered over various locations in the genome (Table 2 wg-set under estimation). The four miRNAs showing underestimation when reads are mapped against *mir-set* instead of *wg-set*, are all characterized by the presence of differential mapping of reads over paralogs, which is unexpected since the mature form of miR218, miR517a, miR16 as well as the star form of miR509 form are identical between paralogs. Upon a careful check of aligned reads we observed that reads assignment was given only in part based to sequence specificity by the aligner. Specifically we founr that in cases where only one alignment for each read has to be reported and two alignments with the same score are found on the two paralogs, the software will report only the alignment associated with the first sequence found in the reference dataset.

It is notable that the relative behavior of the *wg-set* and the *mir-set* is not dependent on the alignment method used, since it does not change when SHRiMP or MicroRaserS (not shown) are used. The tools miRanalyzer, miRNAkey, miRExpress and miRProf cannot be used to compare whole genome and miRBase as reference, since the option to map against a user defined reference set is not available.

On the basis of the above mentioned results, at the present time and with the available techniques, the use of the whole genome as reference provides results that are not particularly robust in terms of quantitative analysis and therefore miRBase should be preferred as reference set.

### MPS alignment tools

A variety of primary mapping tools, i.e. software mapping short reads to a reference set of sequences, have been made available to the bioinformatics community in the last few years. The ability of primary mapping tools to correctly map the vast majority of short reads is an important point that has to be considered in a quantitative data analysis workflow. In this paper we focused our attention on a set of primary mapping tools specifically devoted to miRNA mapping or having a specific set of parameters for miRNA detection (Table 3). Out of the six software tested by us, four are stand alone applications (SHRiMP [6], miRExpress [9], MicroRazerS [10], and miRNAkey [11]), and two are web services (miRProf [12] and miRanalyser [13]).

**Table 3:** Primary mapping tools evaluated in this paper.

Name	Download site	Version	Reference set	Running time§(mir-set/wg-set)	Spike-in detection rate(mir-set/wg-set)
**SHRIMP**	http://compbio.cs.toronto.edu/shrimp	2.0.1	mir-set/wg-set	4 min/40 min	96%/96%
**MicroRazerS**	http://www.seqan.de/projects/MicroRazerS.html	1.2	mir-set/wg-set	2 min/14 min	96%/96%
**miRNAkey**	http://ibis.tau.ac.il/miRNAkey	1.2	mir-set/NA*	9 min/−	94%/−
**miRExpress**	http://miRExpress.mbc.nctu.edu.tw	2.0.1	mir-set/NA	16 min/−	91%/−
**miRanalyzer**	http://web.bioinformatics.cicbiogune.es/miRNA/miRanalyser.php	Webservice	NA/wg-set	−/−	−/73%
**miRProf**	http://srna-tools.cmp.uea.ac.uk/	Webservice	mir-set/NA	−/−	46%/−

The analyses were done on a server equipped with 16 CPU (4×Quad-Core Intel Xeon E7320 processor 2.13GHz), 132 Gb RAM, running Linux SUSE enterprise 10.

§ Running time is referred to the use of 1 processor for standalone tools. In case of on-line tools running time and number of processors is unknown.

*NA indicates that the specific reference set was not supported by the algorithm.

We mapped A1 to A4 and B1 to B4, i.e. the spike-in set without PBMC background, using the default parameters suggested by the authors and allowing only up to one mismatch. Only the microRNAs with non-zero counts in at least four out of the eight samples were considered as detected and subsequently intersected with the BS benchmark set, allowing the calculation of the efficacy of each tool to detect the spiked-in miRNAs (Table 3). Our data show that SHRiMP and MicroRazerS outperformed the other methods in sensitivity and SHRiMP was ranked also as the fastest among the evaluated tools.

### Filtering

In a miRNA-seq workflow we have two different types of filtering steps: pre and post-alignment.

Pre-alignment filters are mainly used to remove library adaptors, which are present as part of the read sequence since the mature miRNA are usually shorter than 35 nucleotides, which is the average sequencing length used in miRNA-seq. Adaptors need to be removed before alignment to the reference to avoid the loss of a significant number of reads, due to the rejection, during alignment, of reads characterized by more than one mismatch with respect to reference. Since adaptor trimming is a relatively straightforward step, we did not test multiple tools and we focus on the characterization of the performance of a tool that is routinely used in our laboratory: Adapter_trim (see material and methods). To test the ability of this tool to remove adaptors, we constructed a synthetic set of reads, in which the 3′ end adaptor (21 nts) is attached to the end of the human mature set of miRNAs extracted from miRBase (1212 miRNAs). Subsequently all sequences were chopped at the 3′ end to have a length of 35 nts. Therefore, since the mature miRNAs length ranges between 19 and 30 nts we obtained different fragment length of the 3′ end adaptor contaminating miRNA sequences. The trimming was 100% effective in removing adaptor, with limited effect on miRNA sequences, which are lacking the last two nucleotides at 3′ end.

Post alignment filters are usually applied to remove mapped reads containing sequencing errors and mismatches. In this paper we filtered SHRiMP output removing mapped reads containing at least one sequencing error and/or more than one mismatch. However, since the filtering procedure does not contain any critical issue the use of a specific software tool is not mandatory. Furthermore, in cases where the whole genome is used as references we took advantage of the ENSEMBL miRNA annotation (Ensembl Genomes Release 8) to discard all alignments not referring to know miRNAs [14].

### Segmentation algorithms

Segmentation algorithms allow the definition of peaks, i.e. intervals of bases on the reference sequence, on which short reads counts are over-represented. In cases where the *mir-set* is used as reference short read cluster and microRNA precursor sequence are synonymous, therefore the use of a peak segmentation algorithm is not required. Similarly when the ENSEMBL genome is used, if peaks are defined on the basis of the microRNA annotation on the genome, segmentation algorithms are not required. We decided to not consider in this quantitative analysis workflow peak segmentation algorithms, since this analysis is focused on the quantification of known microRNA and their annotation is therefore available.

### Statistical analysis of MPS differential expression

The statistical analysis of differential expression for digital data is a relatively new area, but is a critical issue in a quantitative analysis workflow (Figure 1). We tested five tools (Table 4), all available at Bioconductor (www.bioconductor.org). Four of them were specifically devoted to differential expression detection for MPS data: edgeR and baySeq use a model based on negative binomial distribution to estimate differential expression [15], [16]. DESeq [17] method assumes that the mean is a good predictor of the variance and tests for differences between the base means of two conditions. DEGseq package [18] uses a modified t-test statistics [19] frequently utilized for microarray differential expression detection. The fifth, called rank product (RankProd) [20], is instead a non-parametric statistic efficiently used in microarray differential expression analysis, but never tested for the detection of digital data differential expression.

**Table 4:** True positive and negative miRNAs set.

Group	log2(A/B)	miRNA genes
1	>3.5	20
2	3	17
3	2	20
4	≥1.0;≤1.9	43
5	≥0.1;≤0.9	66
6	0	37
7	≥−0.9;≤−0.1	72
8	≥−1.9;≤−1.0	42
9	−2	18
10	−3	17
11	≤−3.5	21

The Willenbrock's spike-in set was reorganized to have spike-in concentrations recalculated at miRNA precursor-level.

The efficacy of the five tools in detecting miRNAs differential expression was carried out on the mapping data produced by SHRiMP using the Willenbrock spike-in set after mixing them with four sets of reads derived from miRNA MPS sequencing of healthy donor PBMC to simulate biological background (Table 1). We used receiver operating characteristic (ROC) curves [21] to evaluate sensitivity and specificity of the above mentioned statistics.

**Figure 3:**
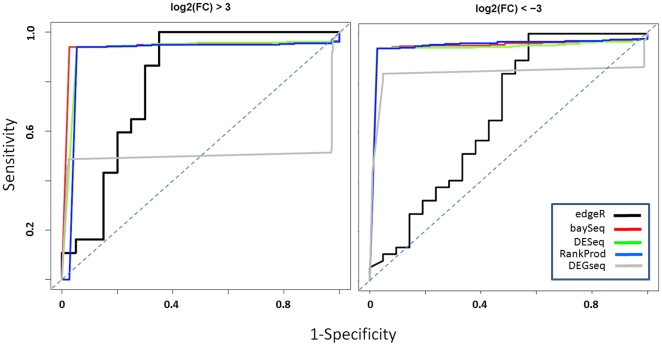
Efficacy of detecting differentially expressed miRNAs. The ability of edgeR, DEGseq, DESeq, baySeq and RankProd to detect differential expression in presence of absolute log_2_ fold change >3 folds was evaluated by mean of ROC analysis.

**Figure 4:**
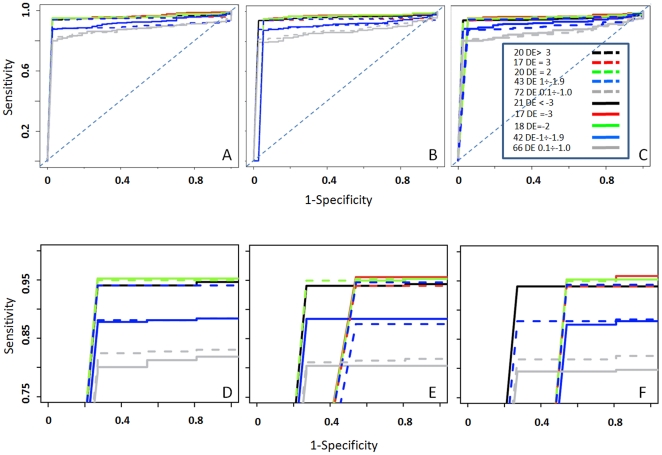
ROC curves describing differential expression for baySeq (A), RankProd (B) and DESeq (C). D-F as A-C but zooming above 75% sensitivity and below 10% 1-specificity. The legend shows the number of expected differentially expressed miRNAs associated to each of the 10 groups of spike-in and the corresponding expected log_2_ fold change variation range.

Furthermore, we used BS benchmark set to evaluate the ability of the statistics to detect miRNAs differential expression. Initially we evaluated the ability of the five statistics to detect differential expression in presence of wide expression changes between the samples A and B, i.e. absolute log_2_ fold change >3 folds (Figure 3, Table 4, groups 1 and 11). On the basis of this analysis was clear that baySeq, DESeq and RankProd were very efficient in detecting miRNAs differential expression. Instead performance of DEGseq and edgeR were lower (Fig. 3 black and grey curves). We refined this analysis focusing on the three methods that gave the best performances, looking at their ability to detect differential expression over a range of fold changes (Table 4, Figure 4). The three tools performed quite efficiently over the all ranges of fold change variations, although RankProd shows a slightly lower specificity (Fig. 4B) with respect to the other two methods (Fig. 4A, C).

Sensitivity is clearly associated with the absolute range of fold change variation (Fig. 4 D–F). It is notable that sensitivity, for the three statistics, moves from 80% sensitivity, in case of absolute fold changes lower than 1, to above 94% sensitivity for fold changes greater than 2. Furthermore baySeq outperforms the other statistics tools for the false positive rate that always remains below 25% for all fold change ranges.

**Figure 5:**
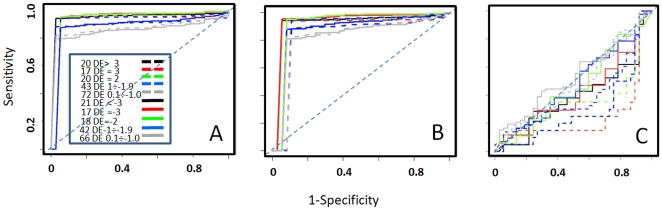
ROC curve of the sample size effect for RankProd. A) Four replicates for each experimental condition. B) Two replicates for each experimental condition, using background bk0. C) Two replicates for each experimental condition, using background bk1.

We also tested the dependency of the three tools performances of the basis of sample size. We used various combination of backgrounds: bk0 (A1BG1 and A2BG2 versus B2BG2 and B4BG4) was designed to combine a small sample size with a library size unbalance (Table 1), but with a limited background variability, i.e. BG2 is present in both experimental groups. bk1 to bk8 combine the same true set with different backgrounds (additional information S3). Interestingly baySeq (additional information S4) and DESeq (additional information S5) performed very well independently from the background considered. Rank Product instead showed a very strong dependency on the background (Figure 5 and additional information S6). Already in the presence of 4 replications for each group Rank Product is characterized by a slightly reduced specificity, however in the case of a small sample size the increase of sample to sample variability, which is greater in bk1 to bk8 completely destroys the ability to detect differential expression.

**Figure 6:**
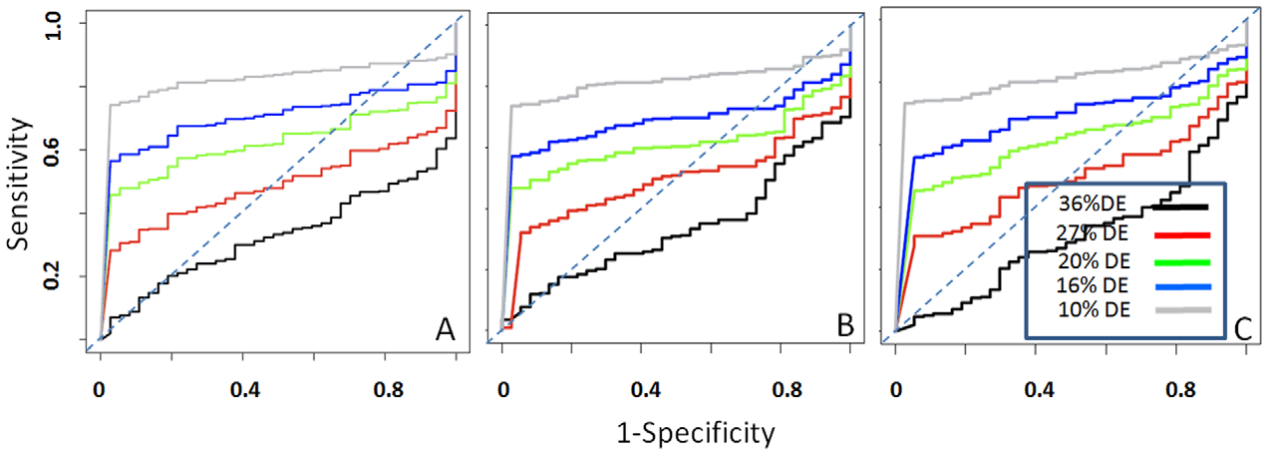
ROC curves describing the effect of an increasing number of differentially expressed miRNAs: baySeq (A), RankProd (B) and DESeq (C). Legend shows the ratio between expected differentially expressed miRNA and the full set of mapped miRNAs.

We also tested the effect of an increasing number of expected differentially expressed miRNAs on the ability to efficiently detect differential expression. All three tested methods are very sensitive to an increase in the number of expected differentially expressed miRNAs (Fig. 6). Already with 10% of expected differential expression (Fig. 6 black curve) the efficacy of the tests was degraded.

### Software implementation

Any data manipulation, i.e. data reformatting and statistical analyses, done on the output data produced by the various alignment tools, i.e. SHRiMP, miRExpress, MicroRazerS, miRProf and miRanalyzer, was implemented in oneChannelGUI [22] Bioconductor package. oneChannelGUI was designed specifically for life scientists who are not familiar with R language but do wish to capitalize on the vast analysis opportunities of Bioconductor. It was designed to provide an interface for multiplatform microarray data analysis and it now also allows secondary analysis of digital data.

## Discussion

Since we have already demonstrated the efficacy of semi-synthetic datasets in defining the performances of workflow for high throughput transcription data, by dissecting an exon-level analysis workflow for Affymetrix 1.0 ST arrays [23], we applied a similar approach to the workflow for quantification of microRNAs digital MPS data. Our results indicate that the use of a focused reference data set, i.e. the miRbase microRNA precursor set, is quite important to guarantee a precise and specific counts detection. Furthermore, we highlighted that the selection of the alignment software is very important to maximize the detection rate of the microRNAs. Our results clearly indicate that SHRiMP and MicroRazerS provide the best miRNA detection rate. Concerning the statistical detection of differential expression of digital data we observed that different statistical approaches specifically designed for digital data, as the NB model implemented in the baySeq package and the variance model implemented in DESeq, perform quite efficiently in the detection of differential expression for digital data. Performances of the above mentioned methods are retained even in presence of a very small sample size. We have also showed that the non-parametric method based on ranking implemented in RankProd, an approach frequently used in differential expression in microarray based transcription profiling, when applied to digital data proved to be very sensitive to background composition.

**Figure 7:**
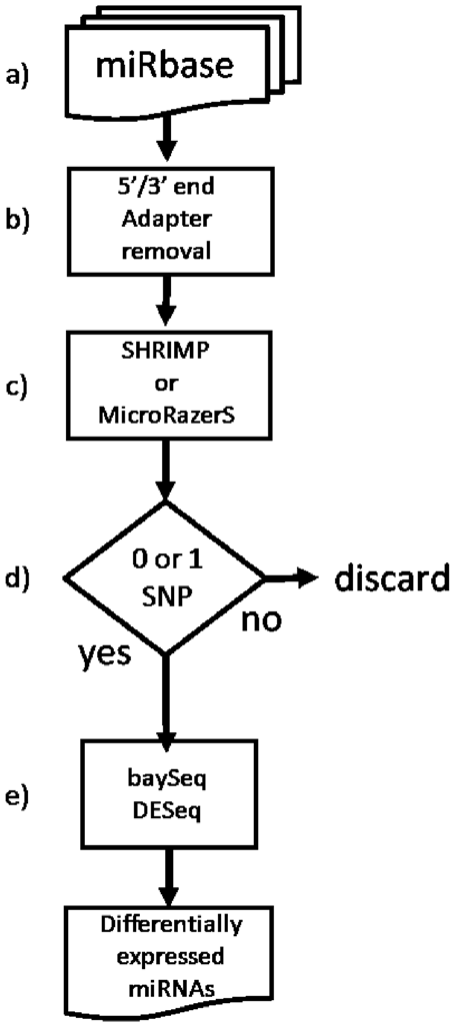
Optimized microRNA differential expression analysis workflow for digital data. a) reference sequence, b) post-processing filter, c) alignment tool, d) post-processing filter, e) differential expression statistics.

The considerations discussed so far leads to definition of the optimized workflow for quantitative detection of microRNA differential expression (Figure 7). Furthermore, although we used Illumina data to evaluate the various steps of the analysis workflow, the proposed pipeline is not platform dependent, therefore it can be applied to data derived using other highthroughput sequencing technologies, e.g. 454 (Roche) and SOLiD (ABI).

## Materials and Methods

### Reference sequences

The whole unmasked human genome, release hg19, (*wg-set*) was retrieved from ftp://ftp.ncbi.nih.gov/genomes/H_sapiens/Assembled_chromosomes/; the miRNA precursors subset for mirBase 15.0 (*mir-set*) was retrieved from http://www.mirbase.org/and reformatted to produce a fasta file having as sequence names only the miRNA symbol.

### Datasets

Barcoded sets (1–4) of short reads for A and B experiments were retrieved from GEO (www.ncbi.nih.gov/geo), series GSE14511 [1]. Since on GEO the short reads are deposited after linker removal, we combined for each barcoded sample the reads of length between 21 and 32 nts. The Willenbrock spike-in set contains a total of 36 non-annotated mature miRNAs and 708 annotated mature miRNAs (additional information S1). The spike-in set also contains mature miRNA located in the 5′ end of the loop (−5p), mature miRNA located in the 3′ end of the loop (−3p) and short mature miRNA (*). Since in this paper mapping procedures are based on alignment over miRNA precursors, nominal spike-in concentrations were recalculated at miRNA precursor-level. We defined a total of 427 miRNA genes (benchmark set BS; additional information S2), which could be associated with unique miRNA ENSEMBL gene identifiers, using ENSEMBL release 62. The 427 miRNAs are organized into 11 groups on the basis of the log_2_ fold change variation between A and B experiments (Table 3, additional information S2). Each of the Willenbrock barcoded short reads set (A1 to A4 and B1 to B4) was mixed with four sets of reads derived from miRNA MPS sequencing of healthy donor PBMC (BG1 to BG4), as shown in Table 1. Also in this case we kept only reads with length 21–32 after removing the 3′ adaptor sequence using the trimLRPatterns function provided in the ShortRead [24] Bioconductor package.

The miRNA MPS sequencing of healthy donor PBMC (BG1 to BG4) represents the control group of an experiment detecting miRNA associated with Multiple Sclerosis. miRNA libraries were produced using a procedure very similar to that used in the Willenbrock experiment. The Small RNA Sample Prep Kit (Illumina, CA, USA) was used and 35 mer short reads were produced using four lines of GAII platform (Illumina, CA, USA). Fasta files for the BG1 to BG4 sets as well as for Willenbrock A and B sets, with and without association with BG1 to BG4 are available at: http://www.bioinformatica.unito.it/downloads/microRNA.workflow.

### Mapping tools

As primary mapping tools we tested: SHRiMP version 2.0.1 [6], miRExpress version 2.0.1 [9], MicroRazerS version 1.2 [10], miRNAkey version 1.2 [11], miRProf [12] and miRanalyser [13]. All analyses were carried out using the optimal (default) configuration suggested by the developers and allowing the detection of no more than one SNP. Short reads containing sequencing errors were all discarded.

**SHRiMP:** This is a general short reads aligner with specific parameters for miRNA analysis. The aligner first discovers reads candidate mapping locations by a seed scanner, which implements *spaced seeds* [25] and *Q-gram filters* [26], and subsequently validates the alignments by the vectorized Smith-Waterman algorithm [27].

**miRExpress:** miRExpress is a tool made of three modules. The first module allows raw data preprocessing, e.g. adaptor removal. The second module carries out the alignment of all short reads against those of known mature miRNAs. The alignment is done, using as reference miRBase mature miRNAs, by a Smith-Waterman algorithm [27]. The third module organizes miRNA expression profiles by computing the sum of read counts for each miRNA according to the alignment criteria (e.g. the length of the read equals the length of the miRNA sequence and the identity of the alignment is 100%).

**MicroRazerS:** This tool is a special version of the general purpose short read mapping tool RazerS [28]. It is based on a q-gram counting strategy which builds an index over the reads and uses an implementation of the Swift filter algorithm [26] to scan over the reference and efficiently filter regions containing possible read matches. MicroRazerS guarantees the finding of all matches and reports a configurable maximum number of equally best matches. Perfect matches are given preference over matches containing mismatches.

**miRNAkey:** The tool uses SEQ-EM algorithm [29] to optimize the distribution of multiply-aligned-reads among the observed miRNAs, rather than discarding them. Reads counting is generated for each sample (i.e. sequencing lane), and counts are converted into the normalized RPKM expression-index (reads per kilobase-pair per million mapped reads) to allow comparison across experiments. Differential expression for miRNAs between paired samples is quantified using chi-squared analysis. This tool provides, as part of the output, additional information regarding the input data, such as multiple mapping levels and post-clipping read lengths.

**miRProf:** This tool is part of the UEA sRNA toolkit (http://srna-tools.cmp.uea.ac.uk/animal/cgi-bin/srna-tools.cgi) and uses the PatMaN algorithm [30] to perform the searches of short reads against miRBase.

**miRanalyzer:** this tool provides three internal analysis levels: (i) detection and counting of known microRNAs (the mapping is done against miRBase and the tool generates a prefix tree of all input reads and subsequently walks in a single run over the genome to detect the reads), (ii) mapping against libraries of transcribed sequences (mRNA, ncRNA, etc.) and (iii) prediction of new microRNAs.

Where the *wg-set*, i.e. whole genome, was used short reads aggregation and annotation was done with the Bioconductor Genominator package [31], using as peaks definition the miRNA annotation of ENSEMBL, retrieved using the Bioconductor package ChIPpeakAnno [32].

The outputs generated by each of the aligner used in this paper are available at: http://www.bioinformatica.unito.it/downloads/microRNA.workflow.

### Filtering

**Pre-alignment filters:** To trim adaptors we used a modified version of Adapter_trim (http://centre.bioinformatics.zj.cn/mirtools/adaptortrim.php), a perl script that can remove low quality reads, 3′/5′ adapters and polyA from a fastq file and provides as output a fasta file. The modified version of Adapter_trim, is available as part of the oneChannelGUI package [22]. The modifications applied to the original filter simply provide a fastq file as output, instead of a fasta file. The efficacy of the filter was tested on a synthetic fastq file generated using the human mature miRNAs retrieved from miRBase version 15 (1212 miRNAs). The 3′ end Illumina adaptor (TCGTATGCCGTCTTCTGCTTG) was attached to the 3′end of the miRNAs. Sequences were then trimmed, at 3′ end, to be 35 nucleotides (nts) long. Since the size range of miRNAs, in the above mentioned set, is between 19 to 30 nts and the 3′ end Illumina adaptor is 21 nts long in this data set contains adaptor ranging from 5 to 16 nts. The fastq files are available at http://www.bioinformatica.unito.it/downloads/microRNA.workflow.

**Post-alignment filters:** The Post alignment filter is usually applied to remove mapped reads containing sequencing errors, mismatches or low reads. An R script was used on SHRiMP output to remove reads containing at least one sequencing error or more than one mismatch. Furthermore, in cases where the whole genome was used as reference sequence only the subset of genomic locations associated with miRNA genes were considered, thus discarding all other non-coding RNA types.

### Differential expression

We tested edgeR [15], baySeq [16], DESeq [17], RankProd [20] and DEGseq [18].

**edgeR:** This package provides statistical routines for determining differential expression in digital gene expression data [15] for two and multiple group experimental designs. It takes into account the total read number of each library during the computation of fold-changes, concentration and statistical significance and uses an empirical approach to estimate the bias affecting library size [33]. Differential expression estimation is made using a model based on Negative Binomial distribution (NB). The NB model dispersion represents sample to sample variability and can be estimated for all tags together (common) or in a tag specific way (tagwise). The latter resembles the moderation of gene intensity variance [34] in microarray data.

**baySeq:** this package [16] offers the possibility to define differential expression using both Poisson-Gamma and NB models. Authors highlighted that the NB model is more accurate, although potentially computationally more intensive and thus slower than the Poisson-Gamma model. The main difference with respect to the NB model used in edgeR is the estimation of empirical distribution on the parameters of the NB distribution by bootstrapping from the data and the subsequent acquisition of posterior likelihoods, thus estimating the proportions of differentially expressed counts.

**DESeq:** this package [17] provides a tool to estimate the variance in digital data and tests for differential expression. The core assumption of the method is that the mean is a good predictor of the variance, i.e. that genes with a similar expression level also have similar variance across replicates. Hence, it is necessary to estimate for each condition a function that allows the prediction of the variance from the mean. This estimation is done by calculating, for each gene, the sample mean and variance within replicates and then fitting a curve to this data. The statistics [17] tests for differences between the base means of two conditions.

**RankProd:** this package utilizes the so called rank product non-parametric method [20] to identify up-regulated or down-regulated genes under one condition against another condition. The method was not designed to detect differential expression in digital data but, since it is based on a non-parametric assumptions, we decided to test its efficacy in the detection of miRNA differential expression.

**DEGseq:** this package [18] has a function embedded which detects differential expression using SAM [19], which is a well know tool for microarray data analysis.

Since each package offers multiple analysis conditions we evaluated all of them and we used those giving the best performance in differential expression detection. We used Receiver Operating Characteristic (ROC) curves [21] to evaluate Sensitivity and Specificity of the above methods.
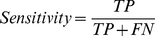
(1)

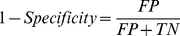
(2)In equation (1) TP and FP are respectively the true positives and the true negatives detected as differentially expressed. In equation (2) TN and FP are respectively the true negatives detected as differentially expressed and those undetected as differentially expressed.

## Supporting Information

Additional Information S1 **Willenbrock's A and B experiments generated using a total of 744 human mature miRNA spiked-in at different concentrations.**
(PDF)

Additional Information S2 **Subset of Willenbrock's A and B experiments which could be associated with a unique miRNA ENSEMBL gene identifier, (427 miRNAs).**
(PDF)

Additional Information S3 **Structure of experiments encompassing the same set of spike-in data with different backgrounds.**
(PDF)

Additional Information S4 **ROC curves describing differential expression for baySeq in presence of different backgrounds.**
(PDF)

Additional Information S5 **ROC curves describing differential expression for DESeq in presence of different backgrounds.**
(PDF)

Additional Information S6 **ROC curves describing differential expression for rank Product in presence of different backgrounds.**
(PDF)
