# Supplementary material for: Optimizing a Massive Parallel Sequencing Workflow for Quantitative miRNA Expression Analysis
Source: PLoS One. 2012 Feb 20;7(2):e31630. doi: 10.1371/journal.pone.0031630 (PMC3282730; doi:10.1371/journal.pone.0031630)
Supplement: Additional Information S1 — Willenbrock's A and B experiments generated using a total of 744 human mature miRNA spiked-in at different concentrations. (PDF) [file pone.0031630.s001.pdf]

#Concentration = concentration of the sequence in amol/ul

mature miRNA	Concentration A	Concentration B	Group	log_2(A/B) mature miRNA	miRNA.gene
hsa-let-7a	266.67	133.33	4	1.00	let-7a
hsa-let-7a*	355.56	44.44	2	3.00	let-7a
hsa-let-7b	10.00	10.00	6	0.00	let-7b
hsa-let-7b*	23.53	376.47	14	-4.00	let-7b
hsa-let-7c	133.33	266.67	11	-1.00	let-7c
hsa-let-7c*	100.00	100.00	8	0.00	let-7c
hsa-let-7d	80.00	320.00	12	-2.00	let-7d
hsa-let-7d*	1.00	1.00	6	0.00	let-7d
hsa-let-7e	234.30	165.70	5	0.50	let-7e
hsa-let-7e*	376.47	23.53	1	4.00	let-7e
hsa-let-7f	44.44	355.56	13	-3.00	let-7f
hsa-let-7f-1*	266.67	133.33	4	1.00	let-7f-1
hsa-let-7f-2*	165.70	234.30	10	-0.50	let-7f-2
hsa-let-7g	376.47	23.53	1	4.00	let7g
hsa-let-7g*	80.00	320.00	12	-2.00	let-7g
hsa-let-7i	133.33	266.67	11	-1.00	let-7i
hsa-let-7i*	234.30	165.70	5	0.50	let-7i
hsa-miR-1	44.44	355.56	13	-3.00	miR-1
hsa-miR-100	1.00	1.00	6	0.00	miR-100
hsa-miR-100*	133.33	266.67	11	-1.00	miR-100
hsa-miR-101	320.00	80.00	3	2.00	miR-101
hsa-miR-101*	165.70	234.30	10	-0.50	miR-101
hsa-miR-103	234.30	165.70	5	0.50	miR-103
hsa-miR-105	320.00	80.00	3	2.00	miR-105
hsa-miR-105*	133.33	266.67	11	-1.00	miR-105
hsa-miR-106a	133.33	266.67	11	-1.00	miR-106a
hsa-miR-106a*	80.00	320.00	12	-2.00	miR-106a
hsa-miR-106b	23.53	376.47	14	-4.00	miR-106b
hsa-miR-106b*	355.56	44.44	2	3.00	miR-106b

hsa-miR-107	1000.00	1000.00	9	0.00	miR-107
hsa-miR-10a	266.67	133.33	4	1.00	miR-10a
hsa-miR-10a*	44.44	355.56	13	-3.00	miR-10a
hsa-miR-10b	100.00	100.00	8	0.00	miR-10b
hsa-miR-10b*	266.67	133.33	4	1.00	miR-10b
hsa-miR-122	355.56	44.44	2	3.00	miR-122
hsa-miR-122*	133.33	266.67	11	-1.00	miR-122
hsa-miR-124	23.53	376.47	14	-4.00	miR-124
hsa-miR-124*	266.67	133.33	4	1.00	miR-124
hsa-miR-125a-3p	23.53	376.47	14	-4.00	miR-125a
hsa-miR-125a-5p	376.47	23.53	1	4.00	miR-125a
hsa-miR-125b	133.33	266.67	11	-1.00	miR-125b
hsa-miR-125b-1*	234.30	165.70	5	0.50	miR-125b-1
hsa-miR-125b-2*	165.70	234.30	10	-0.50	miR-125b-2
hsa-miR-126	165.70	234.30	10	-0.50	miR-126
hsa-miR-126*	1.00	1.00	6	0.00	miR-126
hsa-miR-127-3p	10.00	10.00	7	0.00	miR-127
hsa-miR-127-5p	23.53	376.47	14	-4.00	miR-127
hsa-miR-128a	23.53	376.47	14	-4.00	miR-128a
hsa-miR-129*	320.00	80.00	3	2.00	miR-129
hsa-miR-129-3p	100.00	100.00	8	0.00	miR-129
hsa-miR-129-5p	80.00	320.00	12	-2.00	miR-129
hsa-miR-130a	266.67	133.33	4	1.00	miR-130a
hsa-miR-130a*	80.00	320.00	12	-2.00	miR-130a
hsa-miR-130b	133.33	266.67	11	-1.00	miR-130b
hsa-miR-130b*	355.56	44.44	2	3.00	miR-130b
hsa-miR-132	80.00	320.00	12	-2.00	miR-132
hsa-miR-132*	266.67	133.33	4	1.00	miR-132
hsa-miR-133a	23.53	376.47	14	-4.00	miR-133a
hsa-miR-133b	320.00	80.00	3	2.00	miR-133b
hsa-miR-134	234.30	165.70	5	0.50	miR-134
hsa-miR-135a	100.00	100.00	8	0.00	miR-135a
hsa-miR-135a*	133.33	266.67	11	-1.00	miR-135a

hsa-miR-135b	266.67	133.33	4	1.00	miR-135b
hsa-miR-135b*	133.33	266.67	11	-1.00	miR-135b
hsa-miR-136	165.70	234.30	10	-0.50	miR-136
hsa-miR-136*	355.56	44.44	2	3.00	miR-136
hsa-miR-137	355.56	44.44	2	3.00	miR-137
hsa-miR-138	44.44	355.56	13	-3.00	miR-138
hsa-miR-138-1*	320.00	80.00	3	2.00	miR-138-1
hsa-miR-138-2*	100.00	100.00	8	0.00	miR-138-2
hsa-miR-139-3p	234.30	165.70	5	0.50	miR-139
hsa-miR-139-5p	376.47	23.53	1	4.00	miR-139
hsa-miR-140-3p	1.00	1.00	6	0.00	miR-140
hsa-miR-140-5p	1000.00	1000.00	9	0.00	miR-140
hsa-miR-141	1000.00	1000.00	9	0.00	miR-141
hsa-miR-141*	1.00	1.00	6	0.00	miR-141
hsa-miR-142-3p	355.56	44.44	2	3.00	miR-142
hsa-miR-142-5p	44.44	355.56	13	-3.00	miR-142
hsa-miR-143	320.00	80.00	3	2.00	miR-143
hsa-miR-143*	100.00	100.00	8	0.00	miR-143
hsa-miR-144	133.33	266.67	11	-1.00	miR-144
hsa-miR-144*	266.67	133.33	4	1.00	miR-144
hsa-miR-145	266.67	133.33	4	1.00	miR-145
hsa-miR-145*	100.00	100.00	8	0.00	miR-145
hsa-miR-146a	165.70	234.30	10	-0.50	miR-146a
hsa-miR-146a*	1.00	1.00	6	0.00	miR-146a
hsa-miR-146b-3p	80.00	320.00	12	-2.00	miR-146b
hsa-miR-146b-5p	355.56	44.44	2	3.00	miR-146b
hsa-miR-147	100.00	100.00	8	0.00	miR-147
hsa-miR-147b	266.67	133.33	4	1.00	miR-147b
hsa-miR-148a	266.67	133.33	4	1.00	miR-148a
hsa-miR-148a*	133.33	266.67	11	-1.00	miR-148a
hsa-miR-148b	100.00	100.00	8	0.00	miR-148b
hsa-miR-148b*	320.00	80.00	3	2.00	miR-148b
hsa-miR-149	80.00	320.00	12	-2.00	miR-149

hsa-miR-149*	100.00	100.00	8	0.00	miR-149
hsa-miR-150	1.00	1.00	6	0.00	miR-150
hsa-miR-150*	23.53	376.47	14	-4.00	miR-150
hsa-miR-151-3p	320.00	80.00	3	2.00	miR-151
hsa-miR-151-5p	100.00	100.00	8	0.00	miR-151
hsa-miR-152	133.33	266.67	11	-1.00	miR-152
hsa-miR-153	100.00	100.00	8	0.00	miR-153
hsa-miR-154	266.67	133.33	4	1.00	miR-154
hsa-miR-154*	355.56	44.44	2	3.00	miR-154
hsa-miR-155	234.30	165.70	5	0.50	miR-155
hsa-miR-155*	80.00	320.00	12	-2.00	miR-155
hsa-miR-15a	266.67	133.33	4	1.00	miR-15a
hsa-miR-15a*	23.53	376.47	14	-4.00	miR-15a
hsa-miR-15b	165.70	234.30	10	-0.50	miR-15b
hsa-miR-15b*	100.00	100.00	8	0.00	miR-15b
hsa-miR-16	80.00	320.00	12	-2.00	miR-16
hsa-miR-16-1*	10.00	10.00	7	0.00	miR-16-1
hsa-miR-16-2*	320.00	80.00	3	2.00	miR-16-2
hsa-miR-17	1.00	1.00	6	0.00	miR-17
hsa-miR-17*	320.00	80.00	3	2.00	miR-17
hsa-miR-181a	80.00	320.00	12	-2.00	miR-181a
hsa-miR-181a*	133.33	266.67	11	-1.00	miR-181a
hsa-miR-181a-2*	266.67	133.33	4	1.00	miR-181a-2
hsa-miR-181b	100.00	100.00	8	0.00	miR-181b
hsa-miR-181c	10.00	10.00	7	0.00	miR-181c
hsa-miR-181c*	234.30	165.70	5	0.50	miR-181c
hsa-miR-181d	376.47	23.53	1	4.00	miR-181d
hsa-miR-182	133.33	266.67	11	-1.00	miR-182
hsa-miR-182*	376.47	23.53	1	4.00	miR-182
hsa-miR-183	266.67	133.33	4	1.00	miR-183
hsa-miR-183*	165.70	234.30	10	-0.50	miR-183
hsa-miR-184	23.53	376.47	14	-4.00	miR-184
hsa-miR-185	234.30	165.70	5	0.50	miR-185

hsa-miR-185*	266.67	133.33	4	1.00	miR-185
hsa-miR-186	320.00	80.00	3	2.00	miR-186
hsa-miR-186*	133.33	266.67	11	-1.00	miR-186
hsa-miR-187	1.00	1.00	6	0.00	miR-187
hsa-miR-187*	80.00	320.00	12	-2.00	miR-187
hsa-miR-188-3p	320.00	80.00	3	2.00	miR-188
hsa-miR-188-5p	80.00	320.00	12	-2.00	miR-188
hsa-miR-18a	234.30	165.70	5	0.50	miR-18a
hsa-miR-18a*	234.30	165.70	5	0.50	miR-18a
hsa-miR-18b	1000.00	1000.00	9	0.00	miR-18b
hsa-miR-18b*	133.33	266.67	11	-1.00	miR-18b
hsa-miR-190	100.00	100.00	8	0.00	miR-190
hsa-miR-190b	1.00	1.00	6	0.00	miR-190b
hsa-miR-191	266.67	133.33	4	1.00	miR-191
hsa-miR-191*	165.70	234.30	10	-0.50	miR-191
hsa-miR-192	133.33	266.67	11	-1.00	miR-192
hsa-miR-192*	266.67	133.33	4	1.00	miR-192
hsa-miR-193a-3p	376.47	23.53	1	4.00	miR-193a
hsa-miR-193a-5p	133.33	266.67	11	-1.00	miR-193a
hsa-miR-193b	165.70	234.30	10	-0.50	miR-193b
hsa-miR-193b*	266.67	133.33	4	1.00	miR-193b
hsa-miR-194	100.00	100.00	8	0.00	miR-194
hsa-miR-194*	80.00	320.00	12	-2.00	miR-194
hsa-miR-195	234.30	165.70	5	0.50	miR-195
hsa-miR-195*	133.33	266.67	11	-1.00	miR-195
hsa-miR-196a	1.00	1.00	6	0.00	miR-196a
hsa-miR-196a*	44.44	355.56	13	-3.00	miR-196a
hsa-miR-196b	133.33	266.67	11	-1.00	miR-196b
hsa-miR-197	376.47	23.53	1	4.00	miR-197
hsa-miR-198	44.44	355.56	13	-3.00	miR-198
hsa-miR-199a-3p	10.00	10.00	7	0.00	miR-199a
hsa-miR-199a-5p	100.00	100.00	8	0.00	miR-199a
hsa-miR-199b-5p	355.56	44.44	2	3.00	miR-199b

hsa-miR-19a	133.33	266.67	11	-1.00	miR-19a
hsa-miR-19a*	10.00	10.00	7	0.00	miR-19a
hsa-miR-19b	266.67	133.33	4	1.00	miR-19b
hsa-miR-19b-1*	165.70	234.30	10	-0.50	miR-19b-1
hsa-miR-19b-2*	355.56	44.44	2	3.00	miR-19b-2
hsa-miR-200a	355.56	44.44	2	3.00	miR-200a
hsa-miR-200a*	23.53	376.47	14	-4.00	miR-200a
hsa-miR-200b	44.44	355.56	13	-3.00	miR-200b
hsa-miR-200b*	266.67	133.33	4	1.00	miR-200b
hsa-miR-200c	266.67	133.33	4	1.00	miR-200c
hsa-miR-200c*	133.33	266.67	11	-1.00	miR-200c
hsa-miR-202	320.00	80.00	3	2.00	miR-202
hsa-miR-202*	133.33	266.67	11	-1.00	miR-202
hsa-miR-203	10.00	10.00	7	0.00	miR-203
hsa-miR-204	1000.00	1000.00	9	0.00	miR-204
hsa-miR-205	165.70	234.30	10	-0.50	miR-205
hsa-miR-206	1.00	1.00	6	0.00	miR-206
hsa-miR-208	44.44	355.56	13	-3.00	miR-208
hsa-miR-208b	234.30	165.70	5	0.50	miR-208b
hsa-miR-20a	266.67	133.33	4	1.00	miR-20a
hsa-miR-20a*	266.67	133.33	4	1.00	miR-20a
hsa-miR-20b	133.33	266.67	11	-1.00	miR-20b
hsa-miR-20b*	1000.00	1000.00	9	0.00	miR-20b
hsa-miR-21	320.00	80.00	3	2.00	miR-21
hsa-miR-21*	23.53	376.47	14	-4.00	miR-21
hsa-miR-210	44.44	355.56	13	-3.00	miR-210
hsa-miR-211	320.00	80.00	3	2.00	miR-211
hsa-miR-212	320.00	80.00	3	2.00	miR-212
hsa-miR-214	23.53	376.47	14	-4.00	miR-214
hsa-miR-214*	266.67	133.33	4	1.00	miR-214
hsa-miR-215	266.67	133.33	4	1.00	miR-215
hsa-miR-216a	80.00	320.00	12	-2.00	miR-216a
hsa-miR-216b	234.30	165.70	5	0.50	miR-216b

hsa-miR-217	133.33	266.67	11	-1.00	miR-217
hsa-miR-218	80.00	320.00	12	-2.00	miR-218
hsa-miR-218-1*	165.70	234.30	10	-0.50	miR-218-1
hsa-miR-218-2*	1.00	1.00	6	0.00	miR-218-2
hsa-miR-219-5p	266.67	133.33	4	1.00	miR-219
hsa-miR-219-1-3p	266.67	133.33	4	1.00	miR-219-1
hsa-miR-219-2-3p	23.53	376.47	14	-4.00	miR-219-2
hsa-miR-22	1000.00	1000.00	9	0.00	miR-22
hsa-miR-22*	234.30	165.70	5	0.50	miR-22
hsa-miR-220	10.00	10.00	7	0.00	miR-220
hsa-miR-220b	23.53	376.47	14	-4.00	miR-220b
hsa-miR-220c	44.44	355.56	13	-3.00	miR-220c
hsa-miR-221	44.44	355.56	13	-3.00	miR-221
hsa-miR-221*	320.00	80.00	3	2.00	miR-221
hsa-miR-222	376.47	23.53	1	4.00	miR-222
hsa-miR-222*	1000.00	1000.00	9	0.00	miR-222
hsa-miR-223	23.53	376.47	14	-4.00	miR-223
hsa-miR-223*	266.67	133.33	4	1.00	miR-223
hsa-miR-224	133.33	266.67	11	-1.00	miR-224
hsa-miR-23a	133.33	266.67	11	-1.00	miR-23a
hsa-miR-23a*	133.33	266.67	11	-1.00	miR-23a
hsa-miR-23b	355.56	44.44	2	3.00	miR-23b
hsa-miR-23b*	1.00	1.00	6	0.00	miR-23b
hsa-miR-24	133.33	266.67	11	-1.00	miR-24
hsa-miR-24-1*	1000.00	1000.00	9	0.00	miR-24-1
hsa-miR-24-2*	376.47	23.53	1	4.00	miR-24-2
hsa-miR-25	44.44	355.56	13	-3.00	miR-25
hsa-miR-25*	320.00	80.00	3	2.00	miR-25
hsa-miR-26a	355.56	44.44	2	3.00	miR-26a
hsa-miR-26a-1*	10.00	10.00	7	0.00	miR-26a-1
hsa-miR-26a-2*	23.53	376.47	14	-4.00	miR-26a-2
hsa-miR-26b	1000.00	1000.00	9	0.00	miR-26b
hsa-miR-26b*	165.70	234.30	10	-0.50	miR-26b

hsa-miR-27a	1000.00	1000.00	9	0.00	miR-27a
hsa-miR-27a*	355.56	44.44	2	3.00	miR-27a
hsa-miR-27b	1.00	1.00	6	0.00	miR-27b
hsa-miR-27b*	44.44	355.56	13	-3.00	miR-27b
hsa-miR-28-3p	234.30	165.70	5	0.50	miR-28
hsa-miR-28-5p	133.33	266.67	11	-1.00	miR-28
hsa-miR-296-3p	165.70	234.30	10	-0.50	miR-296
hsa-miR-296-5p	10.00	10.00	7	0.00	miR-296
hsa-miR-298	133.33	266.67	11	-1.00	miR-298
hsa-miR-299-3p	1.00	1.00	6	0.00	miR-299
hsa-miR-299-5p	100.00	100.00	8	0.00	miR-299
hsa-miR-29a	266.67	133.33	4	1.00	miR-29a
hsa-miR-29a*	44.44	355.56	13	-3.00	miR-29a
hsa-miR-29b	266.67	133.33	4	1.00	miR-29b
hsa-miR-29b-1*	355.56	44.44	2	3.00	miR-29b-1
hsa-miR-29b-2*	80.00	320.00	12	-2.00	miR-29b-2
hsa-miR-29c	100.00	100.00	8	0.00	miR-29c
hsa-miR-29c*	1.00	1.00	6	0.00	miR-29c
hsa-miR-300	23.53	376.47	14	-4.00	miR-300
hsa-miR-301a	1000.00	1000.00	9	0.00	miR-301a
hsa-miR-301b	266.67	133.33	4	1.00	miR-301b
hsa-miR-302a	376.47	23.53	1	4.00	miR-302a
hsa-miR-302a*	133.33	266.67	11	-1.00	miR-302a
hsa-miR-302b	23.53	376.47	14	-4.00	miR-302b
hsa-miR-302b*	133.33	266.67	11	-1.00	miR-302b
hsa-miR-302c	100.00	100.00	8	0.00	miR-302c
hsa-miR-302c*	44.44	355.56	13	-3.00	miR-302c
hsa-miR-302d	234.30	165.70	5	0.50	miR-302d
hsa-miR-302d*	266.67	133.33	4	1.00	miR-302d
hsa-miR-30a	10.00	10.00	7	0.00	miR-30a
hsa-miR-30a*	266.67	133.33	4	1.00	miR-30a
hsa-miR-30b	44.44	355.56	13	-3.00	miR-30b
hsa-miR-30b*	320.00	80.00	3	2.00	miR-30b

hsa-miR-30c	266.67	133.33	4	1.00	miR-30c
hsa-miR-30c-1*	133.33	266.67	11	-1.00	miR-30c-1
hsa-miR-30c-2*	10.00	10.00	7	0.00	miR-30c-2
hsa-miR-30d	165.70	234.30	10	-0.50	miR-30d
hsa-miR-30d*	1000.00	1000.00	9	0.00	miR-30d
hsa-miR-30e	376.47	23.53	1	4.00	miR-30e
hsa-miR-30e*	80.00	320.00	12	-2.00	miR-30e
hsa-miR-31	44.44	355.56	13	-3.00	miR-31
hsa-miR-31*	234.30	165.70	5	0.50	miR-31
hsa-miR-32	10.00	10.00	7	0.00	miR-32
hsa-miR-32*	80.00	320.00	12	-2.00	miR-32
hsa-miR-320	133.33	266.67	11	-1.00	miR-320
hsa-miR-323-3p	234.30	165.70	5	0.50	miR-323
hsa-miR-323-5p	100.00	100.00	8	0.00	miR-323
hsa-miR-324-3p	320.00	80.00	3	2.00	miR-324
hsa-miR-324-5p	1000.00	1000.00	9	0.00	miR-324
hsa-miR-325	1000.00	1000.00	9	0.00	miR-325
hsa-miR-326	234.30	165.70	5	0.50	miR-326
hsa-miR-328	266.67	133.33	4	1.00	miR-328
hsa-miR-329	44.44	355.56	13	-3.00	miR-329
hsa-miR-330-3p	165.70	234.30	10	-0.50	miR-330
hsa-miR-330-5p	266.67	133.33	4	1.00	miR-330
hsa-miR-331-3p	133.33	266.67	11	-1.00	miR-331
hsa-miR-331-5p	10.00	10.00	7	0.00	miR-331
hsa-miR-335	133.33	266.67	11	-1.00	miR-335
hsa-miR-335*	376.47	23.53	1	4.00	miR-335
hsa-miR-337-3p	165.70	234.30	10	-0.50	miR-337
hsa-miR-337-5p	10.00	10.00	7	0.00	miR-337
hsa-miR-338-3p	80.00	320.00	12	-2.00	miR-338
hsa-miR-338-5p	320.00	80.00	3	2.00	miR-338
hsa-miR-339-3p	23.53	376.47	14	-4.00	miR-339
hsa-miR-339-5p	10.00	10.00	7	0.00	miR-339
hsa-miR-33a	376.47	23.53	1	4.00	miR-33a

hsa-miR-33a*	100.00	100.00	8	0.00	miR-33a
hsa-miR-33b	44.44	355.56	13	-3.00	miR-33b
hsa-miR-33b*	376.47	23.53	1	4.00	miR-33b
hsa-miR-340	100.00	100.00	8	0.00	miR-340
hsa-miR-340*	320.00	80.00	3	2.00	miR-340
hsa-miR-342-3p	376.47	23.53	1	4.00	miR-342
hsa-miR-342-5p	23.53	376.47	14	-4.00	miR-342
hsa-miR-345	133.33	266.67	11	-1.00	miR-345
hsa-miR-346	100.00	100.00	8	0.00	miR-346
hsa-miR-34a	165.70	234.30	10	-0.50	miR-34a
hsa-miR-34a*	355.56	44.44	2	3.00	miR-34a
hsa-miR-34b	266.67	133.33	4	1.00	miR-34b
hsa-miR-34b*	266.67	133.33	4	1.00	miR-34b
hsa-miR-34c-3p	1000.00	1000.00	9	0.00	miR-34c
hsa-miR-34c-5p	23.53	376.47	14	-4.00	miR-34c
hsa-miR-361-3p	10.00	10.00	7	0.00	miR-361
hsa-miR-361-5p	376.47	23.53	1	4.00	miR-361
hsa-miR-362-3p	234.30	165.70	5	0.50	miR-362
hsa-miR-362-5p	23.53	376.47	14	-4.00	miR-362
hsa-miR-363	234.30	165.70	5	0.50	miR-363
hsa-miR-363*	80.00	320.00	12	-2.00	miR-363
hsa-miR-365	1000.00	1000.00	9	0.00	miR-365
hsa-miR-367	80.00	320.00	12	-2.00	miR-367
hsa-miR-367*	355.56	44.44	2	3.00	miR-367
hsa-miR-369-3p	165.70	234.30	10	-0.50	miR-369
hsa-miR-369-5p	133.33	266.67	11	-1.00	miR-369
hsa-miR-370	23.53	376.47	14	-4.00	miR-370
hsa-miR-371-3p	266.67	133.33	4	1.00	miR-371
hsa-miR-371-5p	80.00	320.00	12	-2.00	miR-371
hsa-miR-372	1000.00	1000.00	9	0.00	miR-372
hsa-miR-373	355.56	44.44	2	3.00	miR-373
hsa-miR-373*	1.00	1.00	6	0.00	miR-373
hsa-miR-374a	266.67	133.33	4	1.00	miR-374a

hsa-miR-374a*	165.70	234.30	10	-0.50	miR-374a
hsa-miR-374b	133.33	266.67	11	-1.00	miR-374b
hsa-miR-374b*	1.00	1.00	6	0.00	miR-374b
hsa-miR-375	266.67	133.33	4	1.00	miR-375
hsa-miR-376a	320.00	80.00	3	2.00	miR-376a
hsa-miR-376a*	165.70	234.30	10	-0.50	miR-376a
hsa-miR-376b	133.33	266.67	11	-1.00	miR-376b
hsa-miR-376c	80.00	320.00	12	-2.00	miR-376c
hsa-miR-377	266.67	133.33	4	1.00	miR-377
hsa-miR-377*	80.00	320.00	12	-2.00	miR-377
hsa-miR-378	44.44	355.56	13	-3.00	miR-378
hsa-miR-378*	234.30	165.70	5	0.50	miR-378
hsa-miR-379	266.67	133.33	4	1.00	miR-379
hsa-miR-379*	266.67	133.33	4	1.00	miR-379
hsa-miR-380	355.56	44.44	2	3.00	miR-380
hsa-miR-380*	23.53	376.47	14	-4.00	miR-380
hsa-miR-381	320.00	80.00	3	2.00	miR-381
hsa-miR-382	133.33	266.67	11	-1.00	miR-382
hsa-miR-383	1000.00	1000.00	9	0.00	miR-383
hsa-miR-384	320.00	80.00	3	2.00	miR-384
hsa-miR-409-3p	355.56	44.44	2	3.00	miR-409
hsa-miR-409-5p	1000.00	1000.00	9	0.00	miR-409
hsa-miR-410	100.00	100.00	8	0.00	miR-410
hsa-miR-411	165.70	234.30	10	-0.50	miR-411
hsa-miR-411*	23.53	376.47	14	-4.00	miR-411
hsa-miR-412	1000.00	1000.00	9	0.00	miR-412
hsa-miR-421	266.67	133.33	4	1.00	miR-421
hsa-miR-422a	320.00	80.00	3	2.00	miR-422a
hsa-miR-423-3p	266.67	133.33	4	1.00	miR-423
hsa-miR-423-5p	1000.00	1000.00	9	0.00	miR-423
hsa-miR-424	355.56	44.44	2	3.00	miR-424
hsa-miR-424*	1000.00	1000.00	9	0.00	miR-424
hsa-miR-425	100.00	100.00	8	0.00	miR-425

hsa-miR-425*	23.53	376.47	14	-4.00	miR-425
hsa-miR-429	133.33	266.67	11	-1.00	miR-429
hsa-miR-431	1.00	1.00	6	0.00	miR-431
hsa-miR-431*	320.00	80.00	3	2.00	miR-431
hsa-miR-432	376.47	23.53	1	4.00	miR-432
hsa-miR-432*	234.30	165.70	5	0.50	miR-432
hsa-miR-433	1000.00	1000.00	9	0.00	miR-433
hsa-miR-448	165.70	234.30	10	-0.50	miR-448
hsa-miR-449a	133.33	266.67	11	-1.00	miR-449a
hsa-miR-449b	376.47	23.53	1	4.00	miR-449b
hsa-miR-450a	1.00	1.00	6	0.00	miR-450a
hsa-miR-450b-3p	320.00	80.00	3	2.00	miR-450b
hsa-miR-450b-5p	100.00	100.00	8	0.00	miR-450b
hsa-miR-451	1000.00	1000.00	9	0.00	miR-451
hsa-miR-452	133.33	266.67	11	-1.00	miR-452
hsa-miR-452*	1000.00	1000.00	9	0.00	miR-452
hsa-miR-453	1.00	1.00	6	0.00	miR-453
hsa-miR-454	23.53	376.47	14	-4.00	miR-454
hsa-miR-454*	320.00	80.00	3	2.00	miR-454
hsa-miR-455-3p	266.67	133.33	4	1.00	miR-455
hsa-miR-455-5p	44.44	355.56	13	-3.00	miR-455
hsa-miR-483-3p	133.33	266.67	11	-1.00	miR-483
hsa-miR-483-5p	10.00	10.00	7	0.00	miR-483
hsa-miR-484	234.30	165.70	5	0.50	miR-484
hsa-miR-485-3p	133.33	266.67	11	-1.00	miR-485
hsa-miR-485-5p	376.47	23.53	1	4.00	miR-485
hsa-miR-486-3p	44.44	355.56	13	-3.00	miR-486
hsa-miR-486-5p	266.67	133.33	4	1.00	miR-486
hsa-miR-487a	10.00	10.00	7	0.00	miR-487a
hsa-miR-487b	44.44	355.56	13	-3.00	miR-487b
hsa-miR-488	133.33	266.67	11	-1.00	miR-488
hsa-miR-488*	266.67	133.33	4	1.00	miR-488
hsa-miR-489	133.33	266.67	11	-1.00	miR-489

hsa-miR-490-3p	376.47	23.53	1	4.00	miR-490
hsa-miR-490-5p	165.70	234.30	10	-0.50	miR-490
hsa-miR-491-3p	23.53	376.47	14	-4.00	miR-491
hsa-miR-491-5p	266.67	133.33	4	1.00	miR-491
hsa-miR-492	376.47	23.53	1	4.00	miR-492
hsa-miR-493	133.33	266.67	11	-1.00	miR-493
hsa-miR-493*	10.00	10.00	7	0.00	miR-493
hsa-miR-494	23.53	376.47	14	-4.00	miR-494
hsa-miR-495	1000.00	1000.00	9	0.00	miR-495
hsa-miR-496	10.00	10.00	7	0.00	miR-496
hsa-miR-497	266.67	133.33	4	1.00	miR-497
hsa-miR-497*	100.00	100.00	8	0.00	miR-497
hsa-miR-498	44.44	355.56	13	-3.00	miR-498
hsa-miR-499-3p	1.00	1.00	6	0.00	miR-499
hsa-miR-499-5p	133.33	266.67	11	-1.00	miR-499
hsa-miR-500	165.70	234.30	10	-0.50	miR-500
hsa-miR-500*	100.00	100.00	8	0.00	miR-500
hsa-miR-501-3p	266.67	133.33	4	1.00	miR-501
hsa-miR-501-5p	1.00	1.00	6	0.00	miR-501
hsa-miR-502-3p	80.00	320.00	12	-2.00	miR-502
hsa-miR-502-5p	266.67	133.33	4	1.00	miR-502
hsa-miR-503	266.67	133.33	4	1.00	miR-503
hsa-miR-504	165.70	234.30	10	-0.50	miR-504
hsa-miR-505	10.00	10.00	7	0.00	miR-505
hsa-miR-505*	80.00	320.00	12	-2.00	miR-505
hsa-miR-506	80.00	320.00	12	-2.00	miR-506
hsa-miR-507	133.33	266.67	11	-1.00	miR-507
hsa-miR-508-3p	133.33	266.67	11	-1.00	miR-508
hsa-miR-508-5p	320.00	80.00	3	2.00	miR-508
hsa-miR-509-3p	266.67	133.33	4	1.00	miR-509
hsa-miR-509-5p	376.47	23.53	1	4.00	miR-509
hsa-miR-509-3-5p	1000.00	1000.00	9	0.00	miR-509-3
hsa-miR-510	23.53	376.47	14	-4.00	miR-510

hsa-miR-511	165.70	234.30	10	-0.50	miR-511
hsa-miR-512-3p	10.00	10.00	7	0.00	miR-512
hsa-miR-512-5p	100.00	100.00	8	0.00	miR-512
hsa-miR-513-3p	1.00	1.00	6	0.00	miR-513
hsa-miR-513-5p	355.56	44.44	2	3.00	miR-513
hsa-miR-514	44.44	355.56	13	-3.00	miR-514
hsa-miR-515-3p	10.00	10.00	7	0.00	miR-515
hsa-miR-515-5p	1000.00	1000.00	9	0.00	miR-515
hsa-miR-516a-3p	100.00	100.00	8	0.00	miR-516a
hsa-miR-516a-5p	100.00	100.00	8	0.00	miR-516a
hsa-miR-516b	266.67	133.33	4	1.00	miR-516b
hsa-miR-517*	266.67	133.33	4	1.00	miR-517
hsa-miR-517a	165.70	234.30	10	-0.50	miR-517a
hsa-miR-517b	234.30	165.70	5	0.50	miR-517b
hsa-miR-517c	355.56	44.44	2	3.00	miR-517c
hsa-miR-518d-5p	23.53	376.47	14	-4.00	miR-518
hsa-miR-518a-3p	100.00	100.00	8	0.00	miR-518a
hsa-miR-518a-5p	320.00	80.00	3	2.00	miR-518a
hsa-miR-518b	266.67	133.33	4	1.00	miR-518b
hsa-miR-518c	133.33	266.67	11	-1.00	miR-518c
hsa-miR-518c*	266.67	133.33	4	1.00	miR-518c
hsa-miR-518d-3p	80.00	320.00	12	-2.00	miR-518d
hsa-miR-518e	1000.00	1000.00	9	0.00	miR-518e
hsa-miR-518e*	1.00	1.00	6	0.00	miR-518e
hsa-miR-518f	44.44	355.56	13	-3.00	miR-518f
hsa-miR-518f*	133.33	266.67	11	-1.00	miR-518f
hsa-miR-519a	1.00	1.00	6	0.00	miR-519a
hsa-miR-519b-3p	1000.00	1000.00	9	0.00	miR-519b
hsa-miR-519c-3p	320.00	80.00	3	2.00	miR-519c
hsa-miR-519d	376.47	23.53	1	4.00	miR-519d
hsa-miR-519e	133.33	266.67	11	-1.00	miR-519e
hsa-miR-519e*	266.67	133.33	4	1.00	miR-519e
hsa-miR-520a-3p	1.00	1.00	6	0.00	miR-520a

hsa-miR-520a-5p	376.47	23.53	1	4.00	miR-520a
hsa-miR-520c-3p	10.00	10.00	7	0.00	miR-520c
hsa-miR-520d-3p	355.56	44.44	2	3.00	miR-520d
hsa-miR-520d-5p	44.44	355.56	13	-3.00	miR-520d
hsa-miR-520e	165.70	234.30	10	-0.50	miR-520e
hsa-miR-520f	320.00	80.00	3	2.00	miR-520f
hsa-miR-520g	44.44	355.56	13	-3.00	miR-520g
hsa-miR-521	266.67	133.33	4	1.00	miR-521
hsa-miR-522	266.67	133.33	4	1.00	miR-522
hsa-miR-523	133.33	266.67	11	-1.00	miR-523
hsa-miR-524-3p	165.70	234.30	10	-0.50	miR-524
hsa-miR-524-5p	234.30	165.70	5	0.50	miR-524
hsa-miR-525-3p	376.47	23.53	1	4.00	miR-525
hsa-miR-525-5p	133.33	266.67	11	-1.00	miR-525
hsa-miR-526b	80.00	320.00	12	-2.00	miR-526b
hsa-miR-526b*	355.56	44.44	2	3.00	miR-526b
hsa-miR-532-3p	1000.00	1000.00	9	0.00	miR-532
hsa-miR-532-5p	234.30	165.70	5	0.50	miR-532
hsa-miR-539	266.67	133.33	4	1.00	miR-539
hsa-miR-541	44.44	355.56	13	-3.00	miR-541
hsa-miR-541*	133.33	266.67	11	-1.00	miR-541
hsa-miR-542-3p	133.33	266.67	11	-1.00	miR-542
hsa-miR-542-5p	376.47	23.53	1	4.00	miR-542
hsa-miR-543	320.00	80.00	3	2.00	miR-543
hsa-miR-544	133.33	266.67	11	-1.00	miR-544
hsa-miR-545	80.00	320.00	12	-2.00	miR-545
hsa-miR-545*	165.70	234.30	10	-0.50	miR-545
hsa-miR-548a-3p	23.53	376.47	14	-4.00	miR-548a
hsa-miR-548a-5p	1.00	1.00	6	0.00	miR-548a
hsa-miR-548b-3p	234.30	165.70	5	0.50	miR-548b
hsa-miR-548b-5p	100.00	100.00	8	0.00	miR-548b
hsa-miR-548c-3p	133.33	266.67	11	-1.00	miR-548c
hsa-miR-548c-5p	133.33	266.67	11	-1.00	miR-548c

hsa-miR-548d-3p	44.44	355.56	13	-3.00	miR-548d
hsa-miR-548d-5p	376.47	23.53	1	4.00	miR-548d
hsa-miR-549	44.44	355.56	13	-3.00	miR-549
hsa-miR-550	100.00	100.00	8	0.00	miR-550
hsa-miR-550*	266.67	133.33	4	1.00	miR-550
hsa-miR-551a	100.00	100.00	8	0.00	miR-551a
hsa-miR-551b	266.67	133.33	4	1.00	miR-551b
hsa-miR-551b*	1.00	1.00	6	0.00	miR-551b
hsa-miR-552	10.00	10.00	7	0.00	miR-552
hsa-miR-553	355.56	44.44	2	3.00	miR-553
hsa-miR-554	165.70	234.30	10	-0.50	miR-554
hsa-miR-555	44.44	355.56	13	-3.00	miR-555
hsa-miR-556-3p	1000.00	1000.00	9	0.00	miR-556
hsa-miR-556-5p	320.00	80.00	3	2.00	miR-556
hsa-miR-557	266.67	133.33	4	1.00	miR-557
hsa-miR-558	1000.00	1000.00	9	0.00	miR-558
hsa-miR-559	1.00	1.00	6	0.00	miR-559
hsa-miR-560	133.33	266.67	11	-1.00	miR-560
hsa-miR-561	80.00	320.00	12	-2.00	miR-561
hsa-miR-562	320.00	80.00	3	2.00	miR-562
hsa-miR-563	1000.00	1000.00	9	0.00	miR-563
hsa-miR-564	1.00	1.00	6	0.00	miR-564
hsa-miR-565	10.00	10.00	7	0.00	miR-565
hsa-miR-566	1000.00	1000.00	9	0.00	miR-566
hsa-miR-567	133.33	266.67	11	-1.00	miR-567
hsa-miR-568	10.00	10.00	7	0.00	miR-568
hsa-miR-569	44.44	355.56	13	-3.00	miR-569
hsa-miR-570	10.00	10.00	7	0.00	miR-570
hsa-miR-571	133.33	266.67	11	-1.00	miR-571
hsa-miR-572	100.00	100.00	8	0.00	miR-572
hsa-miR-573	80.00	320.00	12	-2.00	miR-573
hsa-miR-574-3p	234.30	165.70	5	0.50	miR-574
hsa-miR-574-5p	133.33	266.67	11	-1.00	miR-574

hsa-miR-575	376.47	23.53	1	4.00	miR-575
hsa-miR-576-3p	133.33	266.67	11	-1.00	miR-576
hsa-miR-576-5p	320.00	80.00	3	2.00	miR-576
hsa-miR-577	266.67	133.33	4	1.00	miR-577
hsa-miR-578	320.00	80.00	3	2.00	miR-578
hsa-miR-579	266.67	133.33	4	1.00	miR-579
hsa-miR-580	80.00	320.00	12	-2.00	miR-580
hsa-miR-581	133.33	266.67	11	-1.00	miR-581
hsa-miR-582-3p	80.00	320.00	12	-2.00	miR-582
hsa-miR-582-5p	355.56	44.44	2	3.00	miR-582
hsa-miR-583	1.00	1.00	6	0.00	miR-583
hsa-miR-584	376.47	23.53	1	4.00	miR-584
hsa-miR-585	80.00	320.00	12	-2.00	miR-585
hsa-miR-586	10.00	10.00	7	0.00	miR-586
hsa-miR-587	376.47	23.53	1	4.00	miR-587
hsa-miR-588	10.00	10.00	7	0.00	miR-588
hsa-miR-589	10.00	10.00	7	0.00	miR-589
hsa-miR-589*	1000.00	1000.00	9	0.00	miR-589
hsa-miR-590-3p	165.70	234.30	10	-0.50	miR-590
hsa-miR-590-5p	10.00	10.00	7	0.00	miR-590
hsa-miR-591	80.00	320.00	12	-2.00	miR-591
hsa-miR-592	266.67	133.33	4	1.00	miR-592
hsa-miR-593	266.67	133.33	4	1.00	miR-593
hsa-miR-593*	100.00	100.00	8	0.00	miR-593
hsa-miR-595	80.00	320.00	12	-2.00	miR-595
hsa-miR-596	320.00	80.00	3	2.00	miR-596
hsa-miR-597	133.33	266.67	11	-1.00	miR-597
hsa-miR-598	165.70	234.30	10	-0.50	miR-598
hsa-miR-599	320.00	80.00	3	2.00	miR-599
hsa-miR-600	355.56	44.44	2	3.00	miR-600
hsa-miR-601	133.33	266.67	11	-1.00	miR-601
hsa-miR-602	165.70	234.30	10	-0.50	miR-602
hsa-miR-603	266.67	133.33	4	1.00	miR-603

hsa-miR-604	100.00	100.00	8	0.00	miR-604
hsa-miR-605	1.00	1.00	6	0.00	miR-605
hsa-miR-606	44.44	355.56	13	-3.00	miR-606
hsa-miR-607	44.44	355.56	13	-3.00	miR-607
hsa-miR-608	1000.00	1000.00	9	0.00	miR-608
hsa-miR-609	355.56	44.44	2	3.00	miR-609
hsa-miR-610	165.70	234.30	10	-0.50	miR-610
hsa-miR-611	1.00	1.00	6	0.00	miR-611
hsa-miR-612	1.00	1.00	6	0.00	miR-612
hsa-miR-613	234.30	165.70	5	0.50	miR-613
hsa-miR-614	1.00	1.00	6	0.00	miR-614
hsa-miR-615-3p	133.33	266.67	11	-1.00	miR-615
hsa-miR-615-5p	376.47	23.53	1	4.00	miR-615
hsa-miR-616	80.00	320.00	12	-2.00	miR-616
hsa-miR-616*	266.67	133.33	4	1.00	miR-616
hsa-miR-617	376.47	23.53	1	4.00	miR-617
hsa-miR-618	376.47	23.53	1	4.00	miR-618
hsa-miR-619	355.56	44.44	2	3.00	miR-619
hsa-miR-620	23.53	376.47	14	-4.00	miR-620
hsa-miR-621	10.00	10.00	7	0.00	miR-621
hsa-miR-622	100.00	100.00	8	0.00	miR-622
hsa-miR-623	266.67	133.33	4	1.00	miR-623
hsa-miR-624	165.70	234.30	10	-0.50	miR-624
hsa-miR-624*	10.00	10.00	7	0.00	miR-624
hsa-miR-625	376.47	23.53	1	4.00	miR-625
hsa-miR-625*	133.33	266.67	11	-1.00	miR-625
hsa-miR-626	234.30	165.70	5	0.50	miR-626
hsa-miR-627	234.30	165.70	5	0.50	miR-627
hsa-miR-628-3p	266.67	133.33	4	1.00	miR-628
hsa-miR-628-5p	133.33	266.67	11	-1.00	miR-628
hsa-miR-629	266.67	133.33	4	1.00	miR-629
hsa-miR-629*	80.00	320.00	12	-2.00	miR-629
hsa-miR-630	23.53	376.47	14	-4.00	miR-630

hsa-miR-631	10.00	10.00	7	0.00	miR-631
hsa-miR-632	80.00	320.00	12	-2.00	miR-632
hsa-miR-633	165.70	234.30	10	-0.50	miR-633
hsa-miR-634	266.67	133.33	4	1.00	miR-634
hsa-miR-635	23.53	376.47	14	-4.00	miR-635
hsa-miR-636	23.53	376.47	14	-4.00	miR-636
hsa-miR-637	133.33	266.67	11	-1.00	miR-637
hsa-miR-638	320.00	80.00	3	2.00	miR-638
hsa-miR-639	1.00	1.00	6	0.00	miR-639
hsa-miR-640	266.67	133.33	4	1.00	miR-640
hsa-miR-641	80.00	320.00	12	-2.00	miR-641
hsa-miR-642	44.44	355.56	13	-3.00	miR-642
hsa-miR-643	23.53	376.47	14	-4.00	miR-643
hsa-miR-644	80.00	320.00	12	-2.00	miR-644
hsa-miR-645	266.67	133.33	4	1.00	miR-645
hsa-miR-646	133.33	266.67	11	-1.00	miR-646
hsa-miR-647	44.44	355.56	13	-3.00	miR-647
hsa-miR-648	376.47	23.53	1	4.00	miR-648
hsa-miR-649	1.00	1.00	6	0.00	miR-649
hsa-miR-650	376.47	23.53	1	4.00	miR-650
hsa-miR-651	376.47	23.53	1	4.00	miR-651
hsa-miR-652	44.44	355.56	13	-3.00	miR-652
hsa-miR-653	376.47	23.53	1	4.00	miR-653
hsa-miR-654-3p	100.00	100.00	8	0.00	miR-654
hsa-miR-654-5p	376.47	23.53	1	4.00	miR-654
hsa-miR-655	266.67	133.33	4	1.00	miR-655
hsa-miR-656	1000.00	1000.00	9	0.00	miR-656
hsa-miR-657	376.47	23.53	1	4.00	miR-657
hsa-miR-658	165.70	234.30	10	-0.50	miR-658
hsa-miR-659	100.00	100.00	8	0.00	miR-659
hsa-miR-660	234.30	165.70	5	0.50	miR-660
hsa-miR-661	266.67	133.33	4	1.00	miR-661
hsa-miR-662	320.00	80.00	3	2.00	miR-662

hsa-miR-663	234.30	165.70	5	0.50	miR-663
hsa-miR-665	355.56	44.44	2	3.00	miR-665
hsa-miR-668	1.00	1.00	6	0.00	miR-668
hsa-miR-671-3p	355.56	44.44	2	3.00	miR-671
hsa-miR-671-5p	165.70	234.30	10	-0.50	miR-671
hsa-miR-672	1.00	1.00	6	0.00	miR-672
hsa-miR-674	165.70	234.30	10	-0.50	miR-674
hsa-miR-675	10.00	10.00	7	0.00	miR-675
hsa-miR-7	1.00	1.00	6	0.00	miR-7
hsa-miR-708	165.70	234.30	10	-0.50	miR-708
hsa-miR-708*	355.56	44.44	2	3.00	miR-708
hsa-miR-7-1*	100.00	100.00	8	0.00	miR-7-1
hsa-miR-7-2*	355.56	44.44	2	3.00	miR-7-2
hsa-miR-744	44.44	355.56	13	-3.00	miR-744
hsa-miR-744*	133.33	266.67	11	-1.00	miR-744
hsa-miR-758	266.67	133.33	4	1.00	miR-758
hsa-miR-760	10.00	10.00	7	0.00	miR-760
hsa-miR-765	1000.00	1000.00	9	0.00	miR-765
hsa-miR-766	23.53	376.47	14	-4.00	miR-766
hsa-miR-767-3p	10.00	10.00	7	0.00	miR-767
hsa-miR-767-5p	133.33	266.67	11	-1.00	miR-767
hsa-miR-768-3p	234.30	165.70	5	0.50	miR-768
hsa-miR-768-5p	266.67	133.33	4	1.00	miR-768
hsa-miR-769-3p	44.44	355.56	13	-3.00	miR-769
hsa-miR-769-5p	1000.00	1000.00	9	0.00	miR-769
hsa-miR-770-5p	133.33	266.67	11	-1.00	miR-770
hsa-miR-801	266.67	133.33	4	1.00	miR-801
hsa-miR-802	10.00	10.00	7	0.00	miR-802
hsa-miR-871	1.00	1.00	6	0.00	miR-871
hsa-miR-872	376.47	23.53	1	4.00	miR-872
hsa-miR-873	44.44	355.56	13	-3.00	miR-873
hsa-miR-874	355.56	44.44	2	3.00	miR-874
hsa-miR-875-3p	355.56	44.44	2	3.00	miR-875

hsa-miR-875-5p	44.44	355.56	13	-3.00	miR-875
hsa-miR-876-3p	133.33	266.67	11	-1.00	miR-876
hsa-miR-876-5p	320.00	80.00	3	2.00	miR-876
hsa-miR-877	44.44	355.56	13	-3.00	miR-877
hsa-miR-877*	133.33	266.67	11	-1.00	miR-877
hsa-miR-885-3p	80.00	320.00	12	-2.00	miR-885
hsa-miR-885-5p	266.67	133.33	4	1.00	miR-885
hsa-miR-886-3p	266.67	133.33	4	1.00	miR-886
hsa-miR-886-5p	355.56	44.44	2	3.00	miR-886
hsa-miR-887	355.56	44.44	2	3.00	miR-887
hsa-miR-888	234.30	165.70	5	0.50	miR-888
hsa-miR-888*	165.70	234.30	10	-0.50	miR-888
hsa-miR-889	320.00	80.00	3	2.00	miR-889
hsa-miR-890	100.00	100.00	8	0.00	miR-890
hsa-miR-891a	23.53	376.47	14	-4.00	miR-891a
hsa-miR-891b	234.30	165.70	5	0.50	miR-891b
hsa-miR-892a	23.53	376.47	14	-4.00	miR-892a
hsa-miR-892b	376.47	23.53	1	4.00	miR-892b
hsa-miR-9	376.47	23.53	1	4.00	miR-9
hsa-miR-9*	1000.00	1000.00	9	0.00	miR-9
hsa-miR-920	1.00	1.00	6	0.00	miR-920
hsa-miR-921	133.33	266.67	11	-1.00	miR-921
hsa-miR-922	23.53	376.47	14	-4.00	miR-922
hsa-miR-923	234.30	165.70	5	0.50	miR-923
hsa-miR-924	23.53	376.47	14	-4.00	miR-924
hsa-miR-92a	266.67	133.33	4	1.00	miR-92a
hsa-miR-92a-1*	234.30	165.70	5	0.50	miR-92a-1
hsa-miR-92a-2*	165.70	234.30	10	-0.50	miR-92a-2
hsa-miR-92b	133.33	266.67	11	-1.00	miR-92b
hsa-miR-92b*	10.00	10.00	7	0.00	miR-92b
hsa-miR-93	355.56	44.44	2	3.00	miR-93
hsa-miR-93*	133.33	266.67	11	-1.00	miR-93
hsa-miR-933	10.00	10.00	7	0.00	miR-933

hsa-miR-934	133.33	266.67	11	-1.00	miR-934
hsa-miR-935	44.44	355.56	13	-3.00	miR-935
hsa-miR-936	320.00	80.00	3	2.00	miR-936
hsa-miR-937	266.67	133.33	4	1.00	miR-937
hsa-miR-938	10.00	10.00	7	0.00	miR-938
hsa-miR-939	266.67	133.33	4	1.00	miR-939
hsa-miR-940	80.00	320.00	12	-2.00	miR-940
hsa-miR-941	320.00	80.00	3	2.00	miR-941
hsa-miR-942	355.56	44.44	2	3.00	miR-942
hsa-miR-943	1.00	1.00	6	0.00	miR-943
hsa-miR-944	10.00	10.00	7	0.00	miR-944
hsa-miR-95	1000.00	1000.00	9	0.00	miR-95
hsa-miR-96	1.00	1.00	6	0.00	miR-96
hsa-miR-96*	1000.00	1000.00	9	0.00	miR-96
hsa-miR-98	355.56	44.44	2	3.00	miR-98
hsa-miR-99a	23.53	376.47	14	-4.00	miR-99a
hsa-miR-99a*	320.00	80.00	3	2.00	miR-99a
hsa-miR-99b	234.30	165.70	5	0.50	miR-99b
hsa-miR-99b*	165.70	234.30	10	-0.50	miR-99b
hsa-miRPlus-A1025	376.47	23.53	1	4.00	miRPlus-A1025
hsa-miRPlus-A1026	133.33	266.67	11	-1.00	miRPlus-A1026
hsa-miRPlus-A1027	165.70	234.30	10	-0.50	miRPlus-A1027
hsa-miRPlus-A1031	355.56	44.44	2	3.00	miRPlus-A1031
hsa-miRPlus-A1042	133.33	266.67	11	-1.00	miRPlus-A1042
hsa-miRPlus-A1043	1000.00	1000.00	9	0.00	miRPlus-A1043
hsa-miRPlus-A1056	44.44	355.56	13	-3.00	miRPlus-A1056
hsa-miRPlus-A1065	133.33	266.67	11	-1.00	miRPlus-A1065
hsa-miRPlus-A1066	44.44	355.56	13	-3.00	miRPlus-A1066
hsa-miRPlus-A1072	100.00	100.00	8	0.00	miRPlus-A1072
hsa-miRPlus-A1073	234.30	165.70	5	0.50	miRPlus-A1073
hsa-miRPlus-A1075	266.67	133.33	4	1.00	miRPlus-A1075
hsa-miRPlus-A1083	266.67	133.33	4	1.00	miRPlus-A1083
hsa-miRPlus-A1087	266.67	133.33	4	1.00	miRPlus-A1087

hsa-miRPlus-A1098	355.56	44.44	2	3.00	miRPlus-A1098
hsa-miRPlus-B1114	376.47	23.53	1	4.00	miRPlus-B1114
hsa-miRPlus-C1002	1000.00	1000.00	9	0.00	miRPlus-C1002
hsa-miRPlus-C1005	100.00	100.00	8	0.00	miRPlus-C1005
hsa-miRPlus-C1040	133.33	266.67	11	-1.00	miRPlus-C1040
hsa-miRPlus-C1049	100.00	100.00	8	0.00	miRPlus-C1049
hsa-miRPlus-C1057	355.56	44.44	2	3.00	miRPlus-C1057
hsa-miRPlus-C1066	234.30	165.70	5	0.50	miRPlus-C1066
hsa-miRPlus-C1070	23.53	376.47	14	-4.00	miRPlus-C1070
hsa-miRPlus-C1075	80.00	320.00	12	-2.00	miRPlus-C1075
hsa-miRPlus-C1076	320.00	80.00	3	2.00	miRPlus-C1076
hsa-miRPlus-C1087	23.53	376.47	14	-4.00	miRPlus-C1087
hsa-miRPlus-C1089	133.33	266.67	11	-1.00	miRPlus-C1089
hsa-miRPlus-C1099	355.56	44.44	2	3.00	miRPlus-C1099
hsa-miRPlus-C1100	234.30	165.70	5	0.50	miRPlus-C1100
hsa-miRPlus-C1110	133.33	266.67	11	-1.00	miRPlus-C1110
hsa-miRPlus-C1114	266.67	133.33	4	1.00	miRPlus-C1114
hsa-miRPlus-C1115	234.30	165.70	5	0.50	miRPlus-C1115
hsa-miRPlus-D1036	10.00	10.00	7	0.00	miRPlus-D1036
hsa-miRPlus-D1058	376.47	23.53	1	4.00	miRPlus-D1058
hsa-miRPlus-D1116	80.00	320.00	12	-2.00	miRPlus-D1116
hsa-miRPlus-D1120	1.00	1.00	6	0.00	miRPlus-D1120
