# Supplementary material for: Optimizing a Massive Parallel Sequencing Workflow for Quantitative miRNA Expression Analysis
Source: PLoS One. 2012 Feb 20;7(2):e31630. doi: 10.1371/journal.pone.0031630 (PMC3282730; doi:10.1371/journal.pone.0031630)
Supplement: Additional Information S2 — Subset of Willenbrock's A and B experiments which could be associated with a unique miRNA ENSEMBL gene identifier, (427 miRNAs). (PDF) [file pone.0031630.s002.pdf]

miRNA gene symbol	ENSEMBL geneID	gene concentration A	gene concentration B	$log_2(A/B)$ miRNA gene	fc groups	Type	Present in the benchmark set
miR-181d	ENSG00000207585	376.5	23.5	4.0	1	TP	Yes
miR-197	ENSG00000207709	376.5	23.5	4.0	1	TP	Yes
miR-24-2	ENSG00000209707	376.5	23.5	4.0	1	TP	Yes
miR-361	ENSG00000199051	386.5	33.5	3.5	1	TP	Yes
miR-449b	ENSG00000207728	376.5	23.5	4.0	1	TP	Yes
miR-492	ENSG00000208038	376.5	23.5	4.0	1	TP	Yes
miR-519d	ENSG00000207981	376.5	23.5	4.0	1	TP	Yes
miR-520a	ENSG00000207594	377.5	24.5	3.9	1	TP	Yes
miR-575	ENSG00000207746	376.5	23.5	4.0	1	TP	Yes
miR-584	ENSG00000207714	376.5	23.5	4.0	1	TP	Yes
miR-587	ENSG00000207577	376.5	23.5	4.0	1	TP	Yes
miR-615	ENSG00000207571	376.5	23.5	4.0	1	TP	Yes
miR-617	ENSG00000207763	376.5	23.5	4.0	1	TP	Yes
miR-618	ENSG00000208022	376.5	23.5	4.0	1	TP	Yes
miR-648	ENSG00000207780	376.5	23.5	4.0	1	TP	Yes
miR-650	ENSG00000207836	376.5	23.5	4.0	1	TP	Yes
miR-651	ENSG00000207628	376.5	23.5	4.0	1	TP	Yes
miR-653	ENSG00000208014	376.5	23.5	4.0	1	TP	Yes
miR-657	ENSG00000207736	376.5	23.5	4.0	1	TP	Yes
miR-892b	ENSG00000216098	376.5	23.5	4.0	1	TP	Yes
miR-137	ENSG00000207958	355.6	44.4	3.0	2	TP	Yes
miR-199b	ENSG00000207581	355.6	44.4	3.0	2	TP	Yes
miR-19b-2	ENSG00000207812	355.6	44.4	3.0	2	TP	Yes
miR-23b	ENSG00000207563	356.6	45.4	3.0	2	TP	Yes
miR-29b-1	ENSG00000207748	355.6	44.4	3.0	2	TP	Yes
miR-373	ENSG00000199143	356.6	45.4	3.0	2	TP	Yes
miR-517c	ENSG00000207838	355.6	44.4	3.0	2	TP	Yes
miR-553	ENSG00000207750	355.6	44.4	3.0	2	TP	Yes
miR-600	ENSG00000207740	355.6	44.4	3.0	2	TP	Yes

miR-609	ENSG00000208033	355.6	44.4	3.0	2	TP	Yes
miR-619	ENSG00000207622	355.6	44.4	3.0	2	TP	Yes
miR-665	ENSG00000211521	355.6	44.4	3.0	2	TP	Yes
miR-7-2	ENSG00000207703	355.6	44.4	3.0	2	TP	Yes
miR-874	ENSG00000216009	355.6	44.4	3.0	2	TP	Yes
miR-887	ENSG00000216077	355.6	44.4	3.0	2	TP	Yes
miR-942	ENSG00000215930	355.6	44.4	3.0	2	TP	Yes
miR-98	ENSG00000207787	355.6	44.4	3.0	2	TP	Yes
miR-133b	ENSG00000199080	320.0	80.0	2.0	3	TP	Yes
miR-138-1	ENSG00000207954	320.0	80.0	2.0	3	TP	Yes
miR-16-2	ENSG00000198987	320.0	80.0	2.0	3	TP	Yes
miR-17	ENSG00000207745	321.0	81.0	2.0	3	TP	Yes
miR-211	ENSG00000207702	320.0	80.0	2.0	3	TP	Yes
miR-212	ENSG00000207953	320.0	80.0	2.0	3	TP	Yes
miR-381	ENSG00000199020	320.0	80.0	2.0	3	TP	Yes
miR-422a	ENSG00000199156	320.0	80.0	2.0	3	TP	Yes
miR-431	ENSG00000208001	321.0	81.0	2.0	3	TP	Yes
miR-519c	ENSG00000207788	320.0	80.0	2.0	3	TP	Yes
miR-520f	ENSG00000207555	320.0	80.0	2.0	3	TP	Yes
miR-543	ENSG00000212040	320.0	80.0	2.0	3	TP	Yes
miR-562	ENSG00000207626	320.0	80.0	2.0	3	TP	Yes
miR-578	ENSG00000207559	320.0	80.0	2.0	3	TP	Yes
miR-596	ENSG00000207826	320.0	80.0	2.0	3	TP	Yes
miR-599	ENSG00000207804	320.0	80.0	2.0	3	TP	Yes
miR-638	ENSG00000207972	320.0	80.0	2.0	3	TP	Yes
miR-662	ENSG00000207579	320.0	80.0	2.0	3	TP	Yes
miR-889	ENSG00000216099	320.0	80.0	2.0	3	TP	Yes
miR-936	ENSG00000216083	320.0	80.0	2.0	3	TP	Yes
let-7d	ENSG00000199133	81.0	321.0	-2.0	5	TP	Yes
miR-187	ENSG00000207797	81.0	321.0	-2.0	5	TP	Yes
miR-216a	ENSG00000207798	80.0	320.0	-2.0	5	TP	Yes
miR-29b-2	ENSG00000207790	80.0	320.0	-2.0	5	TP	Yes
miR-302b	ENSG00000199058	156.9	643.1	-2.0	5	TP	Yes

miR-376c	ENSG00000238682	80.0	320.0	-2.0	5	TP	Yes
miR-506	ENSG00000207731	80.0	320.0	-2.0	5	TP	Yes
miR-518d	ENSG00000207747	80.0	320.0	-2.0	5	TP	Yes
miR-561	ENSG00000207951	80.0	320.0	-2.0	5	TP	Yes
miR-573	ENSG00000207697	80.0	320.0	-2.0	5	TP	Yes
miR-580	ENSG00000207756	80.0	320.0	-2.0	5	TP	Yes
miR-585	ENSG00000207619	80.0	320.0	-2.0	5	TP	Yes
miR-591	ENSG00000208025	80.0	320.0	-2.0	5	TP	Yes
miR-595	ENSG00000207637	80.0	320.0	-2.0	5	TP	Yes
miR-632	ENSG00000207928	80.0	320.0	-2.0	5	TP	Yes
miR-641	ENSG00000207631	80.0	320.0	-2.0	5	TP	Yes
miR-644	ENSG00000207997	80.0	320.0	-2.0	5	TP	Yes
miR-940	ENSG00000216095	80.0	320.0	-2.0	5	TP	Yes
miR-1	ENSG00000207569	44.4	355.6	-3.0	6	TP	Yes
miR-210	ENSG00000199038	44.4	355.6	-3.0	6	TP	Yes
miR-220c	ENSG00000216151	44.4	355.6	-3.0	6	TP	Yes
miR-27b	ENSG00000207864	45.4	356.6	-3.0	6	TP	Yes
miR-487b	ENSG00000207754	44.4	355.6	-3.0	6	TP	Yes
miR-498	ENSG00000207869	44.4	355.6	-3.0	6	TP	Yes
miR-520g	ENSG00000207799	44.4	355.6	-3.0	6	TP	Yes
miR-549	ENSG00000208003	44.4	355.6	-3.0	6	TP	Yes
miR-555	ENSG00000207720	44.4	355.6	-3.0	6	TP	Yes
miR-569	ENSG00000207963	44.4	355.6	-3.0	6	TP	Yes
miR-606	ENSG00000207583	44.4	355.6	-3.0	6	TP	Yes
miR-607	ENSG00000207976	44.4	355.6	-3.0	6	TP	Yes
miR-642	ENSG00000207773	44.4	355.6	-3.0	6	TP	Yes
miR-647	ENSG00000207554	44.4	355.6	-3.0	6	TP	Yes
miR-652	ENSG00000208013	44.4	355.6	-3.0	6	TP	Yes
miR-873	ENSG00000215939	44.4	355.6	-3.0	6	TP	Yes
miR-935	ENSG00000215998	44.4	355.6	-3.0	6	TP	Yes
let-7b	ENSG00000207875	33.5	386.5	-3.5	7	TP	Yes
miR-127	ENSG00000207608	33.5	386.5	-3.5	7	TP	Yes
miR-150	ENSG00000207782	24.5	377.5	-3.9	7	TP	Yes

miR-184	ENSG00000207695	23.5	376.5	-4.0	7	TP	Yes
miR-219-2	ENSG00000207955	23.5	376.5	-4.0	7	TP	Yes
miR-220b	ENSG00000215937	23.5	376.5	-4.0	7	TP	Yes
miR-26a-2	ENSG00000207789	23.5	376.5	-4.0	7	TP	Yes
miR-300	ENSG00000215957	23.5	376.5	-4.0	7	TP	Yes
miR-339	ENSG00000199023	33.5	386.5	-3.5	7	TP	Yes
miR-370	ENSG00000199005	23.5	376.5	-4.0	7	TP	Yes
miR-494	ENSG00000194717	23.5	376.5	-4.0	7	TP	Yes
miR-510	ENSG00000207641	23.5	376.5	-4.0	7	TP	Yes
miR-620	ENSG00000207967	23.5	376.5	-4.0	7	TP	Yes
miR-630	ENSG00000207690	23.5	376.5	-4.0	7	TP	Yes
miR-635	ENSG00000207561	23.5	376.5	-4.0	7	TP	Yes
miR-636	ENSG00000207556	23.5	376.5	-4.0	7	TP	Yes
miR-643	ENSG00000208002	23.5	376.5	-4.0	7	TP	Yes
miR-766	ENSG00000211578	23.5	376.5	-4.0	7	TP	Yes
miR-891a	ENSG00000216056	23.5	376.5	-4.0	7	TP	Yes
miR-892a	ENSG00000215943	23.5	376.5	-4.0	7	TP	Yes
miR-922	ENSG00000216042	23.5	376.5	-4.0	7	TP	Yes
miR-190b	ENSG00000215938	1.0	1.0	0.0	4	TN	Yes
miR-206	ENSG00000207604	1.0	1.0	0.0	4	TN	Yes
miR-218-2	ENSG00000207739	1.0	1.0	0.0	4	TN	Yes
miR-453	ENSG00000208004	1.0	1.0	0.0	4	TN	Yes
miR-564	ENSG00000207783	1.0	1.0	0.0	4	TN	Yes
miR-612	ENSG00000207727	1.0	1.0	0.0	4	TN	Yes
miR-639	ENSG00000207707	1.0	1.0	0.0	4	TN	Yes
miR-920	ENSG00000216192	1.0	1.0	0.0	4	TN	Yes
miR-30c-2	ENSG00000199094	10.0	10.0	0.0	4	TN	Yes
miR-520c	ENSG00000207738	10.0	10.0	0.0	4	TN	Yes
miR-552	ENSG00000207941	10.0	10.0	0.0	4	TN	Yes
miR-621	ENSG00000207652	10.0	10.0	0.0	4	TN	Yes
miR-631	ENSG00000207636	10.0	10.0	0.0	4	TN	Yes
miR-760	ENSG00000211575	10.0	10.0	0.0	4	TN	Yes
miR-933	ENSG00000215973	10.0	10.0	0.0	4	TN	Yes

miR-138-2	ENSG00000207649	100.0	100.0	0.0	4	TN	Yes
miR-147	ENSG00000207814	100.0	100.0	0.0	4	TN	Yes
miR-551a	ENSG00000207776	100.0	100.0	0.0	4	TN	Yes
miR-572	ENSG00000207716	100.0	100.0	0.0	4	TN	Yes
miR-604	ENSG00000207612	100.0	100.0	0.0	4	TN	Yes
miR-622	ENSG00000207858	100.0	100.0	0.0	4	TN	Yes
miR-659	ENSG00000207696	100.0	100.0	0.0	4	TN	Yes
miR-125a	ENSG00000208008	400.0	400.0	0.0	4	TN	Yes
miR-192	ENSG00000207648	400.0	400.0	0.0	4	TN	Yes
miR-488	ENSG00000202609	400.0	400.0	0.0	4	TN	Yes
miR-519e	ENSG00000207810	400.0	400.0	0.0	4	TN	Yes
miR-524	ENSG00000207977	400.0	400.0	0.0	4	TN	Yes
miR-99b	ENSG00000207550	400.0	400.0	0.0	4	TN	Yes
miR-24-1	ENSG00000207617	1000.0	1000.0	0.0	4	TN	Yes
miR-301a	ENSG00000207996	1000.0	1000.0	0.0	4	TN	Yes
miR-372	ENSG00000199095	1000.0	1000.0	0.0	4	TN	Yes
miR-383	ENSG00000199127	1000.0	1000.0	0.0	4	TN	Yes
miR-566	ENSG00000207922	1000.0	1000.0	0.0	4	TN	Yes
miR-608	ENSG00000207551	1000.0	1000.0	0.0	4	TN	Yes
miR-765	ENSG00000211581	1000.0	1000.0	0.0	4	TN	Yes
miR-518e	ENSG00000207987	1001.0	1001.0	0.0	4	TN	Yes
miR-589	ENSG00000207973	1010.0	1010.0	0.0	4	TN	Yes
let-7e	ENSG00000198972	610.8	189.2	1.7	NA	TP	No
miR-139	ENSG00000207809	610.8	189.2	1.7	NA	TP	No
miR-143	ENSG00000208035	420.0	180.0	1.2	NA	TP	No
miR-147b	ENSG00000211519	266.7	133.3	1.0	NA	TP	No
miR-148b	ENSG00000199122	420.0	180.0	1.2	NA	TP	No
miR-151	ENSG00000207792	420.0	180.0	1.2	NA	TP	No
miR-154	ENSG00000207978	622.2	177.8	1.8	NA	TP	No
miR-181a-2	ENSG00000207595	266.7	133.3	1.0	NA	TP	No
miR-20a	ENSG00000199149	533.3	266.7	1.0	NA	TP	No
miR-215	ENSG00000207590	266.7	133.3	1.0	NA	TP	No
miR-219-1	ENSG00000199036	266.7	133.3	1.0	NA	TP	No

miR-301b	ENSG00000212102	266.7	133.3	1.0	NA	TP	No
miR-328	ENSG00000207948	266.7	133.3	1.0	NA	TP	No
miR-33a	ENSG00000207932	476.5	123.5	1.9	NA	TP	No
miR-340	ENSG00000198995	420.0	180.0	1.2	NA	TP	No
miR-34b	ENSG00000207811	533.3	266.7	1.0	NA	TP	No
miR-375	ENSG00000198973	266.7	133.3	1.0	NA	TP	No
miR-379	ENSG00000199088	533.3	266.7	1.0	NA	TP	No
miR-421	ENSG00000202566	266.7	133.3	1.0	NA	TP	No
miR-432	ENSG00000207793	610.8	189.2	1.7	NA	TP	No
miR-450b	ENSG00000216001	420.0	180.0	1.2	NA	TP	No
miR-490	ENSG00000207597	542.2	257.8	1.1	NA	TP	No
miR-501	ENSG00000211538	267.7	134.3	1.0	NA	TP	No
miR-503	ENSG00000208005	266.7	133.3	1.0	NA	TP	No
miR-518b	ENSG00000207862	266.7	133.3	1.0	NA	TP	No
miR-522	ENSG00000207806	266.7	133.3	1.0	NA	TP	No
miR-539	ENSG00000202560	266.7	133.3	1.0	NA	TP	No
miR-551b	ENSG00000207717	267.7	134.3	1.0	NA	TP	No
miR-557	ENSG00000207974	266.7	133.3	1.0	NA	TP	No
miR-577	ENSG00000207931	266.7	133.3	1.0	NA	TP	No
miR-579	ENSG00000207956	266.7	133.3	1.0	NA	TP	No
miR-592	ENSG00000207692	266.7	133.3	1.0	NA	TP	No
miR-603	ENSG00000207930	266.7	133.3	1.0	NA	TP	No
miR-623	ENSG00000207719	266.7	133.3	1.0	NA	TP	No
miR-634	ENSG00000207943	266.7	133.3	1.0	NA	TP	No
miR-640	ENSG00000207821	266.7	133.3	1.0	NA	TP	No
miR-645	ENSG00000208018	266.7	133.3	1.0	NA	TP	No
miR-654	ENSG00000207934	476.5	123.5	1.9	NA	TP	No
miR-655	ENSG00000207646	266.7	133.3	1.0	NA	TP	No
miR-661	ENSG00000207574	266.7	133.3	1.0	NA	TP	No
miR-758	ENSG00000211582	266.7	133.3	1.0	NA	TP	No
miR-937	ENSG00000216090	266.7	133.3	1.0	NA	TP	No
miR-939	ENSG00000216133	266.7	133.3	1.0	NA	TP	No
let-7g	ENSG00000199150	456.5	343.5	0.4	NA	TP	No

miR-10b	ENSG00000207744	366.7	233.3	0.7	NA	TP	No
miR-122	ENSG00000207778	488.9	311.1	0.7	NA	TP	No
miR-125b-1	ENSG00000207971	234.3	165.7	0.5	NA	TP	No
miR-130b	ENSG00000207751	488.9	311.1	0.7	NA	TP	No
miR-134	ENSG00000207993	234.3	165.7	0.5	NA	TP	No
miR-136	ENSG00000207942	521.3	278.7	0.9	NA	TP	No
miR-145	ENSG00000207700	366.7	233.3	0.7	NA	TP	No
miR-146b	ENSG00000202569	435.6	364.4	0.3	NA	TP	No
miR-181c	ENSG00000207613	244.3	175.7	0.5	NA	TP	No
miR-182	ENSG00000207990	509.8	290.2	0.8	NA	TP	No
miR-183	ENSG00000207691	432.4	367.6	0.2	NA	TP	No
miR-185	ENSG00000208023	501.0	299.0	0.7	NA	TP	No
miR-186	ENSG00000207721	453.3	346.7	0.4	NA	TP	No
miR-18a	ENSG00000199180	468.6	331.4	0.5	NA	TP	No
miR-191	ENSG00000207605	432.4	367.6	0.2	NA	TP	No
miR-193a	ENSG00000207614	509.8	290.2	0.8	NA	TP	No
miR-193b	ENSG00000207639	432.4	367.6	0.2	NA	TP	No
miR-202	ENSG00000199089	453.3	346.7	0.4	NA	TP	No
miR-208b	ENSG00000215991	234.3	165.7	0.5	NA	TP	No
miR-216b	ENSG00000211520	234.3	165.7	0.5	NA	TP	No
miR-22	ENSG00000199060	1234.3	1165.7	0.1	NA	TP	No
miR-222	ENSG00000207725	1376.5	1023.5	0.4	NA	TP	No
miR-27a	ENSG00000207808	1355.6	1044.4	0.4	NA	TP	No
miR-302a	ENSG00000207927	509.8	290.2	0.8	NA	TP	No
miR-302d	ENSG00000199145	501.0	299.0	0.7	NA	TP	No
miR-30a	ENSG00000207827	276.7	143.3	0.9	NA	TP	No
miR-30e	ENSG00000198974	456.5	343.5	0.4	NA	TP	No
miR-323	ENSG00000199069	334.3	265.7	0.3	NA	TP	No
miR-324	ENSG00000199053	1320.0	1080.0	0.3	NA	TP	No
miR-326	ENSG00000199090	234.3	165.7	0.5	NA	TP	No
miR-330	ENSG00000199066	432.4	367.6	0.2	NA	TP	No
miR-335	ENSG00000199043	509.8	290.2	0.8	NA	TP	No
miR-33b	ENSG00000207839	420.9	379.1	0.2	NA	TP	No

miR-34a	ENSG00000207865	521.3	278.7	0.9	NA	TP	No
miR-367	ENSG00000199169	435.6	364.4	0.3	NA	TP	No
miR-374a	ENSG00000199168	432.4	367.6	0.2	NA	TP	No
miR-409	ENSG00000199107	1355.6	1044.4	0.4	NA	TP	No
miR-423	ENSG00000199071	1266.7	1133.3	0.2	NA	TP	No
miR-424	ENSG00000199097	1355.6	1044.4	0.4	NA	TP	No
miR-484	ENSG00000202641	234.3	165.7	0.5	NA	TP	No
miR-485	ENSG00000208027	509.8	290.2	0.8	NA	TP	No
miR-497	ENSG00000207791	366.7	233.3	0.7	NA	TP	No
miR-508	ENSG00000207589	453.3	346.7	0.4	NA	TP	No
miR-517b	ENSG00000207837	234.3	165.7	0.5	NA	TP	No
miR-525	ENSG00000207711	509.8	290.2	0.8	NA	TP	No
miR-526b	ENSG00000207580	435.6	364.4	0.3	NA	TP	No
miR-532	ENSG00000207758	1234.3	1165.7	0.1	NA	TP	No
miR-542	ENSG00000207784	509.8	290.2	0.8	NA	TP	No
miR-548b	ENSG00000207982	334.3	265.7	0.3	NA	TP	No
miR-556	ENSG00000207729	1320.0	1080.0	0.3	NA	TP	No
miR-576	ENSG00000207988	453.3	346.7	0.4	NA	TP	No
miR-582	ENSG00000202601	435.6	364.4	0.3	NA	TP	No
miR-593	ENSG00000207588	366.7	233.3	0.7	NA	TP	No
miR-613	ENSG00000207983	234.3	165.7	0.5	NA	TP	No
miR-625	ENSG00000207781	509.8	290.2	0.8	NA	TP	No
miR-626	ENSG00000207766	234.3	165.7	0.5	NA	TP	No
miR-627	ENSG00000207712	234.3	165.7	0.5	NA	TP	No
miR-660	ENSG00000207970	234.3	165.7	0.5	NA	TP	No
miR-663	ENSG00000207985	234.3	165.7	0.5	NA	TP	No
miR-671	ENSG00000211517	521.3	278.7	0.9	NA	TP	No
miR-708	ENSG00000211997	521.3	278.7	0.9	NA	TP	No
miR-876	ENSG00000215966	453.3	346.7	0.4	NA	TP	No
miR-891b	ENSG00000216064	234.3	165.7	0.5	NA	TP	No
miR-92a-1	ENSG00000207968	234.3	165.7	0.5	NA	TP	No
miR-93	ENSG00000207757	488.9	311.1	0.7	NA	TP	No
let-7c	ENSG00000199030	233.3	366.7	-0.7	NA	TP	No

let-7i	ENSG00000199179	367.6	432.4	-0.2	NA	TP	No
miR-106b	ENSG00000208036	379.1	420.9	-0.2	NA	TP	No
miR-10a	ENSG00000207777	311.1	488.9	-0.7	NA	TP	No
miR-125b-2	ENSG00000207863	165.7	234.3	-0.5	NA	TP	No
miR-126	ENSG00000199161	166.7	235.3	-0.5	NA	TP	No
miR-130a	ENSG00000208009	346.7	453.3	-0.4	NA	TP	No
miR-132	ENSG00000207724	346.7	453.3	-0.4	NA	TP	No
miR-146a	ENSG00000207936	166.7	235.3	-0.5	NA	TP	No
miR-155	ENSG00000207795	314.3	485.7	-0.6	NA	TP	No
miR-15a	ENSG00000207718	290.2	509.8	-0.8	NA	TP	No
miR-15b	ENSG00000207779	265.7	334.3	-0.3	NA	TP	No
miR-195	ENSG00000207929	367.6	432.4	-0.2	NA	TP	No
miR-19a	ENSG00000207610	143.3	276.7	-0.9	NA	TP	No
miR-19b-1	ENSG00000207560	165.7	234.3	-0.5	NA	TP	No
miR-200a	ENSG00000207607	379.1	420.9	-0.2	NA	TP	No
miR-200b	ENSG00000207730	311.1	488.9	-0.7	NA	TP	No
miR-205	ENSG00000207623	165.7	234.3	-0.5	NA	TP	No
miR-20b	ENSG00000207710	1133.3	1266.7	-0.2	NA	TP	No
miR-21	ENSG00000199004	343.5	456.5	-0.4	NA	TP	No
miR-214	ENSG00000207949	290.2	509.8	-0.8	NA	TP	No
miR-218-1	ENSG00000207732	165.7	234.3	-0.5	NA	TP	No
miR-221	ENSG00000207870	364.4	435.6	-0.3	NA	TP	No
miR-223	ENSG00000207939	290.2	509.8	-0.8	NA	TP	No
miR-25	ENSG00000207547	364.4	435.6	-0.3	NA	TP	No
miR-26b	ENSG00000199121	1165.7	1234.3	-0.1	NA	TP	No
miR-28	ENSG00000207651	367.6	432.4	-0.2	NA	TP	No
miR-296	ENSG00000207950	175.7	244.3	-0.5	NA	TP	No
miR-29a	ENSG00000198981	311.1	488.9	-0.7	NA	TP	No
miR-30b	ENSG00000207582	364.4	435.6	-0.3	NA	TP	No
miR-30d	ENSG00000199153	1165.7	1234.3	-0.1	NA	TP	No
miR-31	ENSG00000199177	278.7	521.3	-0.9	NA	TP	No
miR-331	ENSG00000199172	143.3	276.7	-0.9	NA	TP	No
miR-337	ENSG00000199151	175.7	244.3	-0.5	NA	TP	No

miR-34c	ENSG00000207562	1023.5	1376.5	-0.4	NA	TP	No
miR-363	ENSG00000207572	314.3	485.7	-0.6	NA	TP	No
miR-369	ENSG00000199025	299.0	501.0	-0.7	NA	TP	No
miR-371	ENSG00000199031	346.7	453.3	-0.4	NA	TP	No
miR-377	ENSG00000199015	346.7	453.3	-0.4	NA	TP	No
miR-378	ENSG00000199047	278.7	521.3	-0.9	NA	TP	No
miR-380	ENSG00000198982	379.1	420.9	-0.2	NA	TP	No
miR-448	ENSG00000199001	165.7	234.3	-0.5	NA	TP	No
miR-452	ENSG00000207753	1133.3	1266.7	-0.2	NA	TP	No
miR-454	ENSG00000211514	343.5	456.5	-0.4	NA	TP	No
miR-455	ENSG00000207726	311.1	488.9	-0.7	NA	TP	No
miR-483	ENSG00000207805	143.3	276.7	-0.9	NA	TP	No
miR-486	ENSG00000221035	311.1	488.9	-0.7	NA	TP	No
miR-491	ENSG00000207609	290.2	509.8	-0.8	NA	TP	No
miR-493	ENSG00000207989	143.3	276.7	-0.9	NA	TP	No
miR-500	ENSG00000207785	265.7	334.3	-0.3	NA	TP	No
miR-502	ENSG00000208007	346.7	453.3	-0.4	NA	TP	No
miR-504	ENSG00000207800	165.7	234.3	-0.5	NA	TP	No
miR-517a	ENSG00000207734	165.7	234.3	-0.5	NA	TP	No
miR-520e	ENSG00000207599	165.7	234.3	-0.5	NA	TP	No
miR-554	ENSG00000207606	165.7	234.3	-0.5	NA	TP	No
miR-574	ENSG00000207944	367.6	432.4	-0.2	NA	TP	No
miR-590	ENSG00000207741	175.7	244.3	-0.5	NA	TP	No
miR-598	ENSG00000207600	165.7	234.3	-0.5	NA	TP	No
miR-602	ENSG00000207693	165.7	234.3	-0.5	NA	TP	No
miR-610	ENSG00000207874	165.7	234.3	-0.5	NA	TP	No
miR-616	ENSG00000208028	346.7	453.3	-0.4	NA	TP	No
miR-624	ENSG00000207952	175.7	244.3	-0.5	NA	TP	No
miR-629	ENSG00000207965	346.7	453.3	-0.4	NA	TP	No
miR-633	ENSG00000207552	165.7	234.3	-0.5	NA	TP	No
miR-658	ENSG00000207945	165.7	234.3	-0.5	NA	TP	No
miR-767	ENSG00000211583	143.3	276.7	-0.9	NA	TP	No
miR-769	ENSG00000211580	1044.4	1355.6	-0.4	NA	TP	No

miR-885	ENSG00000216135	346.7	453.3	-0.4	NA	TP	No
miR-92a-2	ENSG00000208034	165.7	234.3	-0.5	NA	TP	No
miR-92b	ENSG00000208011	143.3	276.7	-0.9	NA	TP	No
miR-99a	ENSG00000207638	343.5	456.5	-0.4	NA	TP	No
miR-18b	ENSG00000211137	1133.3	1266.7	-0.2	NA	TP	No
miR-100	ENSG00000207994	134.3	267.7	-1.0	NA	TP	No
miR-106a	ENSG00000207602	213.3	586.7	-1.5	NA	TP	No
miR-149	ENSG00000207611	180.0	420.0	-1.2	NA	TP	No
miR-152	ENSG00000207947	133.3	266.7	-1.0	NA	TP	No
miR-196b	ENSG00000207584	133.3	266.7	-1.0	NA	TP	No
miR-217	ENSG00000207548	133.3	266.7	-1.0	NA	TP	No
miR-224	ENSG00000207621	133.3	266.7	-1.0	NA	TP	No
miR-23a	ENSG00000207980	266.7	533.3	-1.0	NA	TP	No
miR-298	ENSG00000216031	133.3	266.7	-1.0	NA	TP	No
miR-302c	ENSG00000199102	144.4	455.6	-1.7	NA	TP	No
miR-30c-1	ENSG00000207962	133.3	266.7	-1.0	NA	TP	No
miR-32	ENSG00000207698	90.0	330.0	-1.9	NA	TP	No
miR-345	ENSG00000198984	133.3	266.7	-1.0	NA	TP	No
miR-362	ENSG00000208015	257.8	542.2	-1.1	NA	TP	No
miR-374b	ENSG00000212027	134.3	267.7	-1.0	NA	TP	No
miR-382	ENSG00000207742	133.3	266.7	-1.0	NA	TP	No
miR-411	ENSG00000199109	189.2	610.8	-1.7	NA	TP	No
miR-425	ENSG00000199032	123.5	476.5	-1.9	NA	TP	No
miR-429	ENSG00000198976	133.3	266.7	-1.0	NA	TP	No
miR-449a	ENSG00000198983	133.3	266.7	-1.0	NA	TP	No
miR-489	ENSG00000207656	133.3	266.7	-1.0	NA	TP	No
miR-499	ENSG00000207635	134.3	267.7	-1.0	NA	TP	No
miR-505	ENSG00000207633	90.0	330.0	-1.9	NA	TP	No
miR-507	ENSG00000207969	133.3	266.7	-1.0	NA	TP	No
miR-518f	ENSG00000207706	177.8	622.2	-1.8	NA	TP	No
miR-523	ENSG00000208016	133.3	266.7	-1.0	NA	TP	No
miR-541	ENSG00000216179	177.8	622.2	-1.8	NA	TP	No
miR-544	ENSG00000207587	133.3	266.7	-1.0	NA	TP	No

miR-545	ENSG00000207820	245.7	554.3	-1.2	NA	TP	No
miR-548c	ENSG00000207546	266.7	533.3	-1.0	NA	TP	No
miR-567	ENSG00000207940	133.3	266.7	-1.0	NA	TP	No
miR-571	ENSG00000207642	133.3	266.7	-1.0	NA	TP	No
miR-581	ENSG00000207627	133.3	266.7	-1.0	NA	TP	No
miR-597	ENSG00000207701	133.3	266.7	-1.0	NA	TP	No
miR-601	ENSG00000207991	133.3	266.7	-1.0	NA	TP	No
miR-637	ENSG00000207733	133.3	266.7	-1.0	NA	TP	No
miR-646	ENSG00000207802	133.3	266.7	-1.0	NA	TP	No
miR-744	ENSG00000211589	177.8	622.2	-1.8	NA	TP	No
miR-770	ENSG00000211574	133.3	266.7	-1.0	NA	TP	No
miR-877	ENSG00000216101	177.8	622.2	-1.8	NA	TP	No
miR-921	ENSG00000215952	133.3	266.7	-1.0	NA	TP	No
miR-934	ENSG00000216060	133.3	266.7	-1.0	NA	TP	No
miR-107	ENSG00000198997	1000.0	1000.0	0.0	NA	TN	No
miR-135b	ENSG00000199059	400.0	400.0	0.0	NA	TN	No
miR-140	ENSG00000208017	1001.0	1001.0	0.0	NA	TN	No
miR-141	ENSG00000207708	1001.0	1001.0	0.0	NA	TN	No
miR-142	ENSG00000207567	400.0	400.0	0.0	NA	TN	No
miR-144	ENSG00000207618	400.0	400.0	0.0	NA	TN	No
miR-148a	ENSG00000199085	400.0	400.0	0.0	NA	TN	No
miR-16-1	ENSG00000208006	10.0	10.0	0.0	NA	TN	No
miR-188	ENSG00000207768	400.0	400.0	0.0	NA	TN	No
miR-190	ENSG00000222326	100.0	100.0	0.0	NA	TN	No
miR-200c	ENSG00000207713	400.0	400.0	0.0	NA	TN	No
miR-203	ENSG00000207568	10.0	10.0	0.0	NA	TN	No
miR-204	ENSG00000207935	1000.0	1000.0	0.0	NA	TN	No
miR-26a-1	ENSG00000199075	10.0	10.0	0.0	NA	TN	No
miR-299	ENSG00000207749	101.0	101.0	0.0	NA	TN	No
miR-29c	ENSG00000207966	101.0	101.0	0.0	NA	TN	No
miR-325	ENSG00000207995	1000.0	1000.0	0.0	NA	TN	No
miR-338	ENSG00000211563	400.0	400.0	0.0	NA	TN	No
miR-342	ENSG00000199082	400.0	400.0	0.0	NA	TN	No

miR-346	ENSG00000199104	100.0	100.0	0.0	NA	TN	No
miR-410	ENSG00000199092	100.0	100.0	0.0	NA	TN	No
miR-412	ENSG00000199012	1000.0	1000.0	0.0	NA	TN	No
miR-451	ENSG00000207794	1000.0	1000.0	0.0	NA	TN	No
miR-487a	ENSG00000207558	10.0	10.0	0.0	NA	TN	No
miR-495	ENSG00000207743	1000.0	1000.0	0.0	NA	TN	No
miR-496	ENSG00000207961	10.0	10.0	0.0	NA	TN	No
miR-509-3	ENSG00000212014	1000.0	1000.0	0.0	NA	TN	No
miR-518c	ENSG00000207553	400.0	400.0	0.0	NA	TN	No
miR-519b	ENSG00000207825	1000.0	1000.0	0.0	NA	TN	No
miR-520d	ENSG00000207735	400.0	400.0	0.0	NA	TN	No
miR-558	ENSG00000207653	1000.0	1000.0	0.0	NA	TN	No
miR-559	ENSG00000207923	1.0	1.0	0.0	NA	TN	No
miR-563	ENSG00000207815	1000.0	1000.0	0.0	NA	TN	No
miR-570	ENSG00000207650	10.0	10.0	0.0	NA	TN	No
miR-583	ENSG00000207578	1.0	1.0	0.0	NA	TN	No
miR-586	ENSG00000207769	10.0	10.0	0.0	NA	TN	No
miR-588	ENSG00000207632	10.0	10.0	0.0	NA	TN	No
miR-605	ENSG00000207813	1.0	1.0	0.0	NA	TN	No
miR-611	ENSG00000207601	1.0	1.0	0.0	NA	TN	No
miR-614	ENSG00000207817	1.0	1.0	0.0	NA	TN	No
miR-628	ENSG00000207964	400.0	400.0	0.0	NA	TN	No
miR-656	ENSG00000207959	1000.0	1000.0	0.0	NA	TN	No
miR-668	ENSG00000211506	1.0	1.0	0.0	NA	TN	No
miR-675	ENSG00000211502	10.0	10.0	0.0	NA	TN	No
miR-7-1	ENSG00000207603	100.0	100.0	0.0	NA	TN	No
miR-802	ENSG00000211590	10.0	10.0	0.0	NA	TN	No
miR-875	ENSG00000216069	400.0	400.0	0.0	NA	TN	No
miR-888	ENSG00000216005	400.0	400.0	0.0	NA	TN	No
miR-890	ENSG00000216075	100.0	100.0	0.0	NA	TN	No
miR-938	ENSG00000216035	10.0	10.0	0.0	NA	TN	No
miR-943	ENSG00000216105	1.0	1.0	0.0	NA	TN	No
miR-944	ENSG00000216058	10.0	10.0	0.0	NA	TN	No

miR-95	ENSG00000207807	1000.0	1000.0	0.0	NA	TN	No
miR-96	ENSG00000199158	1001.0	1001.0	0.0	NA	TN	No
