# Supplementary material for: Optimizing a Massive Parallel Sequencing Workflow for Quantitative miRNA Expression Analysis
Source: PLoS One. 2012 Feb 20;7(2):e31630. doi: 10.1371/journal.pone.0031630 (PMC3282730; doi:10.1371/journal.pone.0031630)
Supplement: Additional Information S3 — Structure of experiments encompassing the same set of spike-in data with different backgrounds. (PDF) [file pone.0031630.s003.pdf]

Table S3: Datasets built to evaluate the effect of background data on statistics performance

True positive set	A1	A2	B3	B4
**bk1**	BG1	BG2	BG3	BG4
**bk2**	BG2	BG3	BG4	BG1
**bk3**	BG3	BG4	BG1	BG2
**bk4**	BG4	BG1	BG2	BG3
**bk5**	BG1+BG3	BG2	BG3	BG4
**bk6**	BG1	BG2+BG4	BG3	BG4
**bk7**	BG1	BG2+BG4	BG3+BG1	BG4
**bk8**	BG1+BG3	BG2+BG4	BG3	BG4
