# Supplementary figures and images for: Optimizing a Massive Parallel Sequencing Workflow for Quantitative miRNA Expression Analysis
Source: PLoS One. 2012 Feb 20;7(2):e31630. doi: 10.1371/journal.pone.0031630 (PMC3282730; doi:10.1371/journal.pone.0031630)

# baySeq bk1

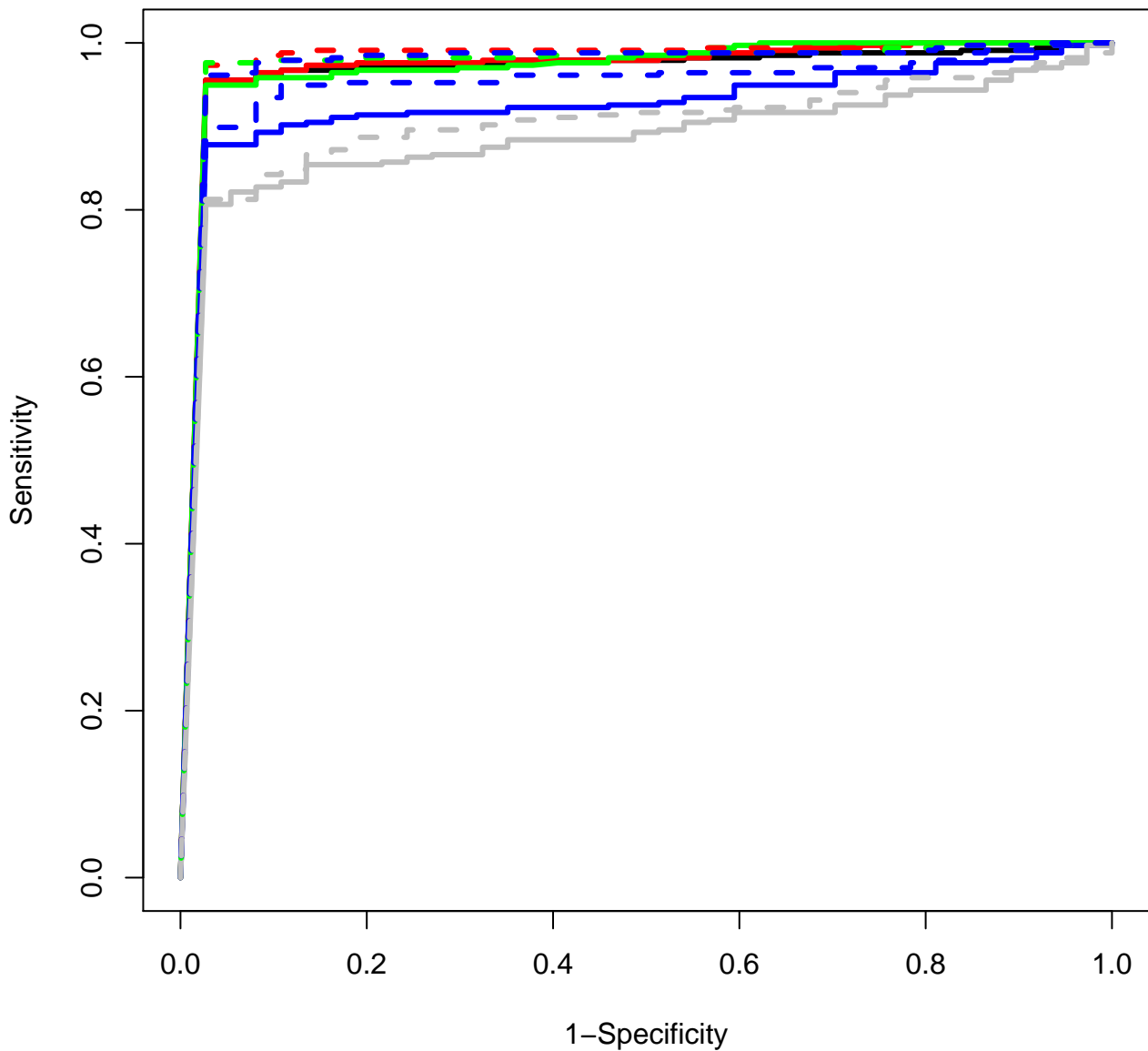

baySeq bk2

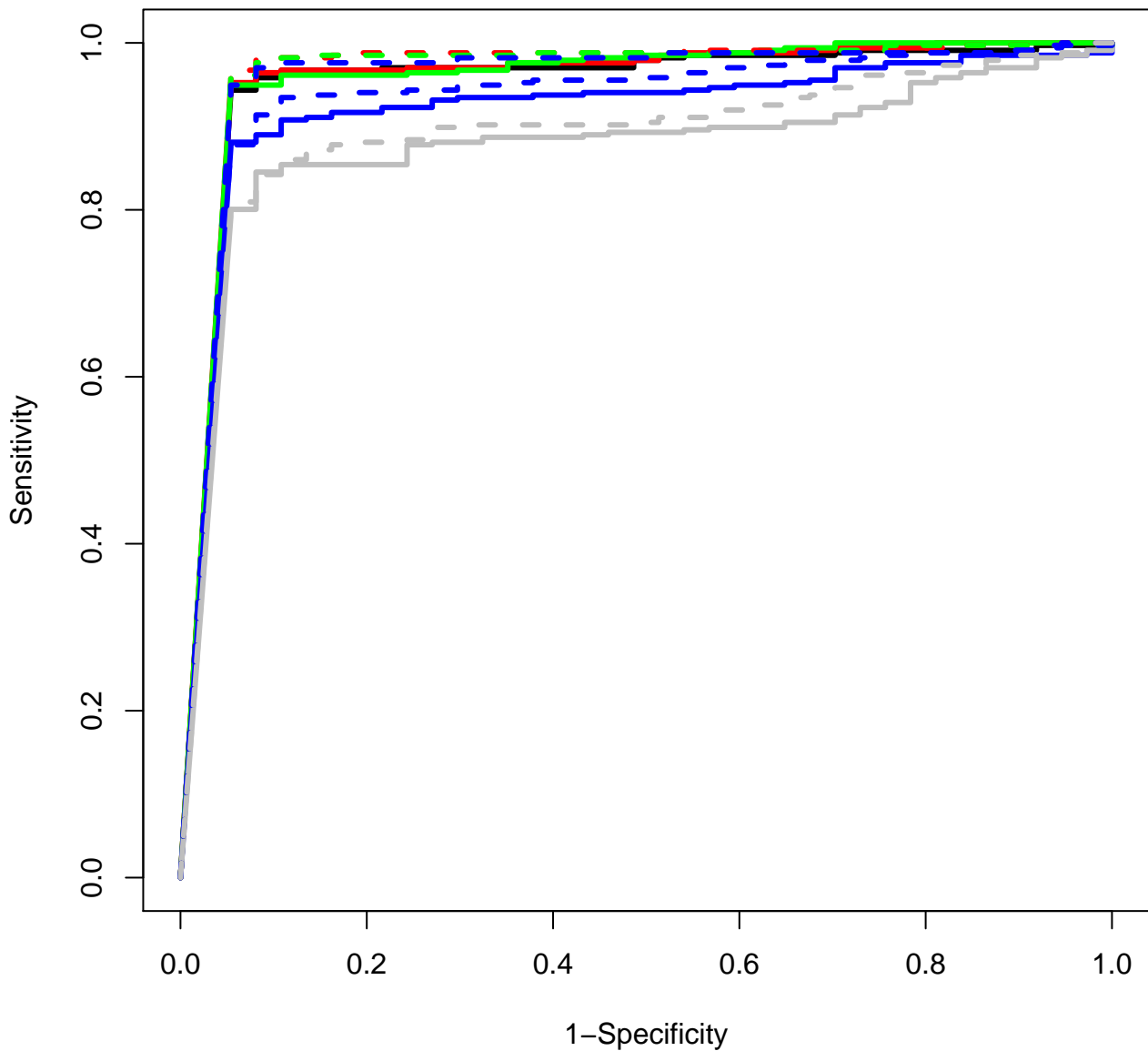

## baySeq bk3

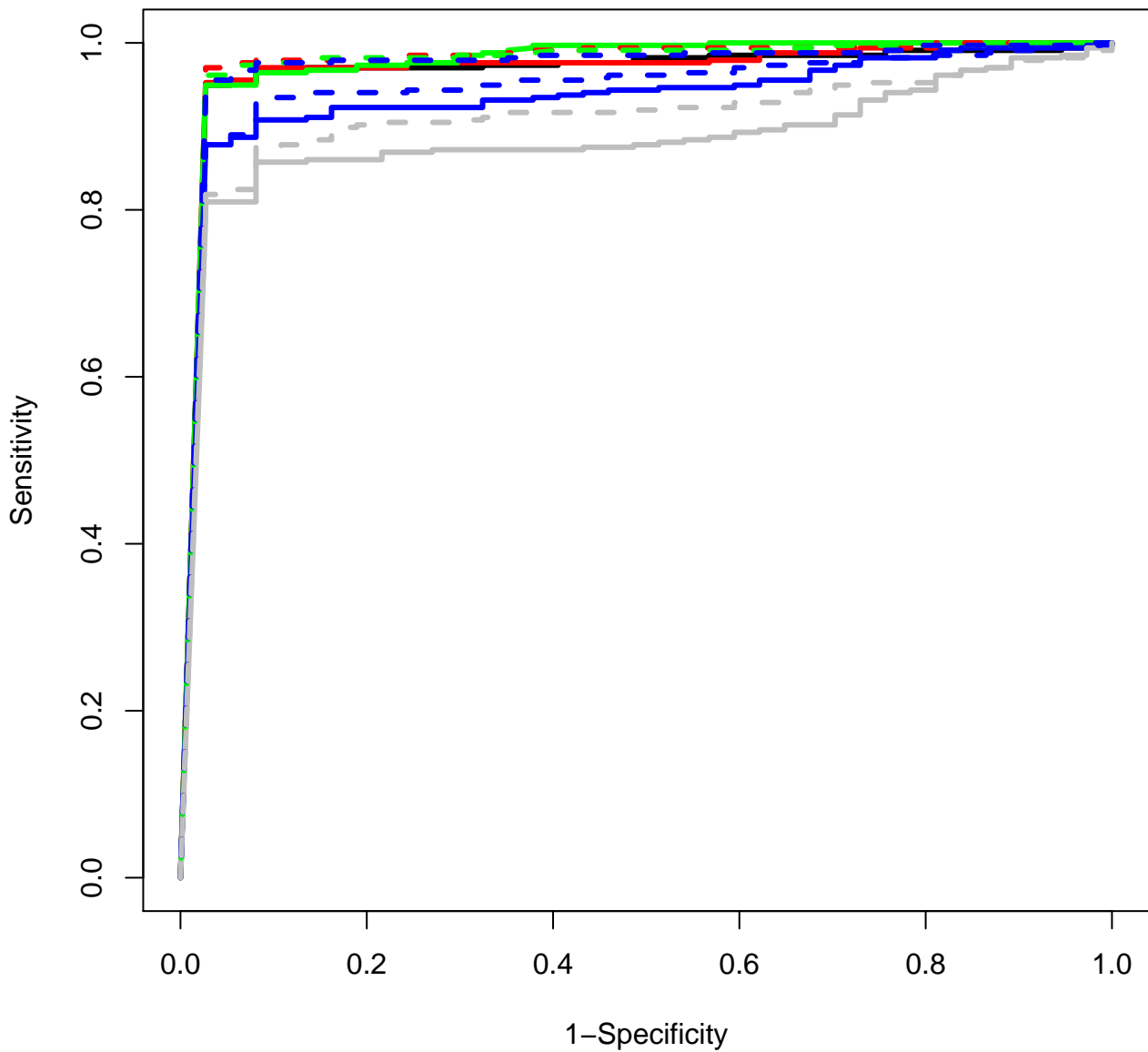

## baySeq bk4

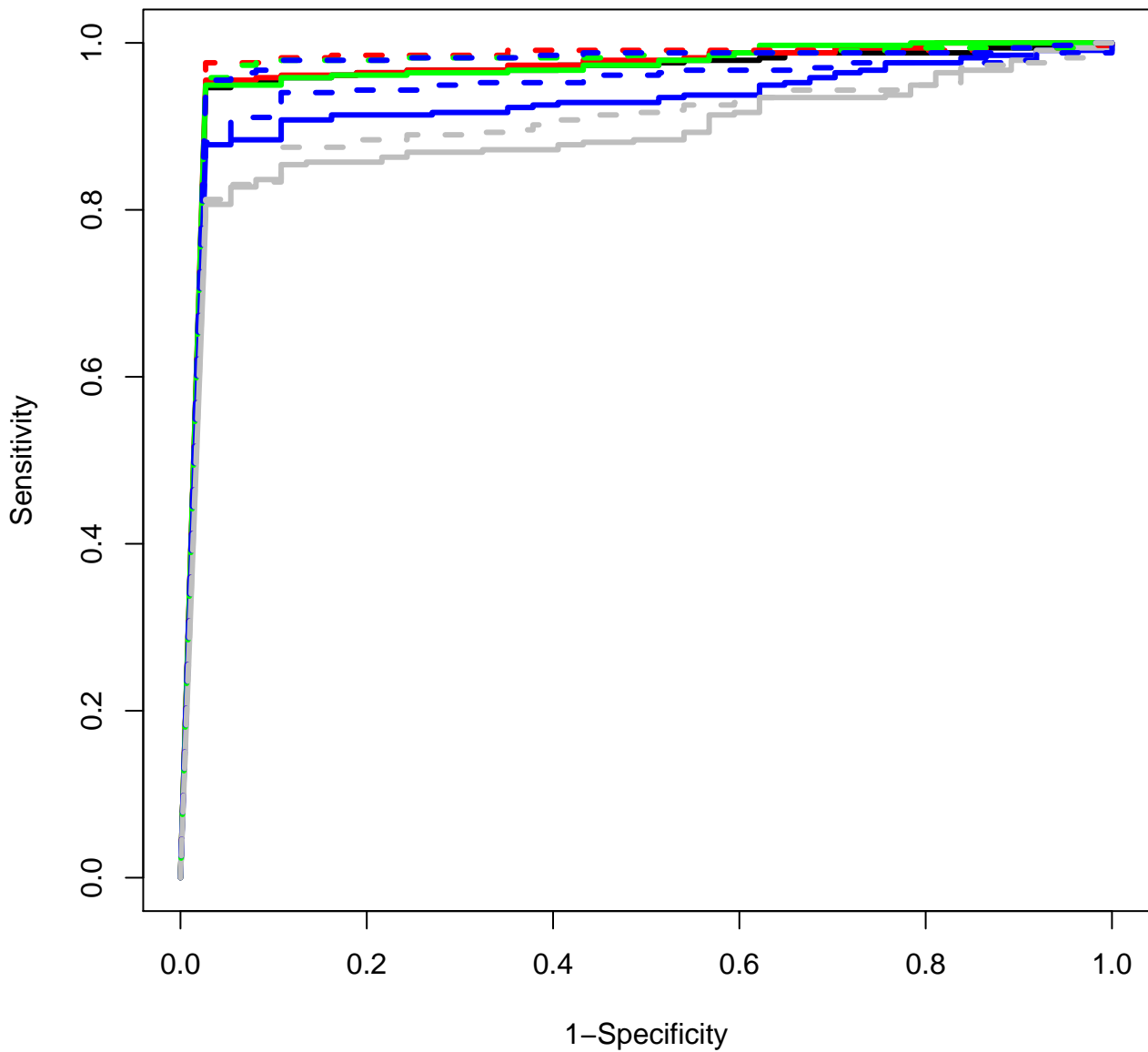

baySeq bk5

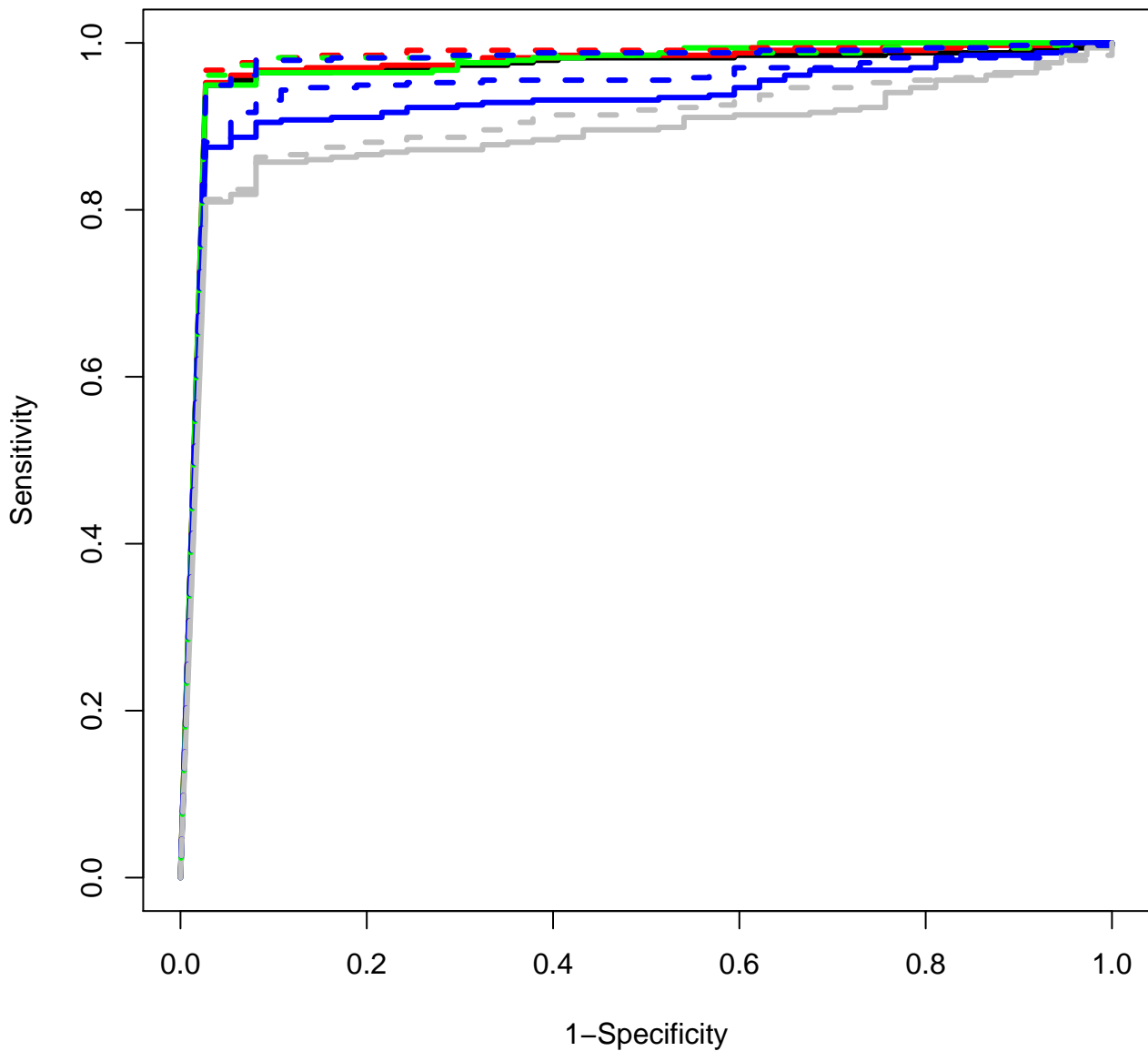

baySeq bk6

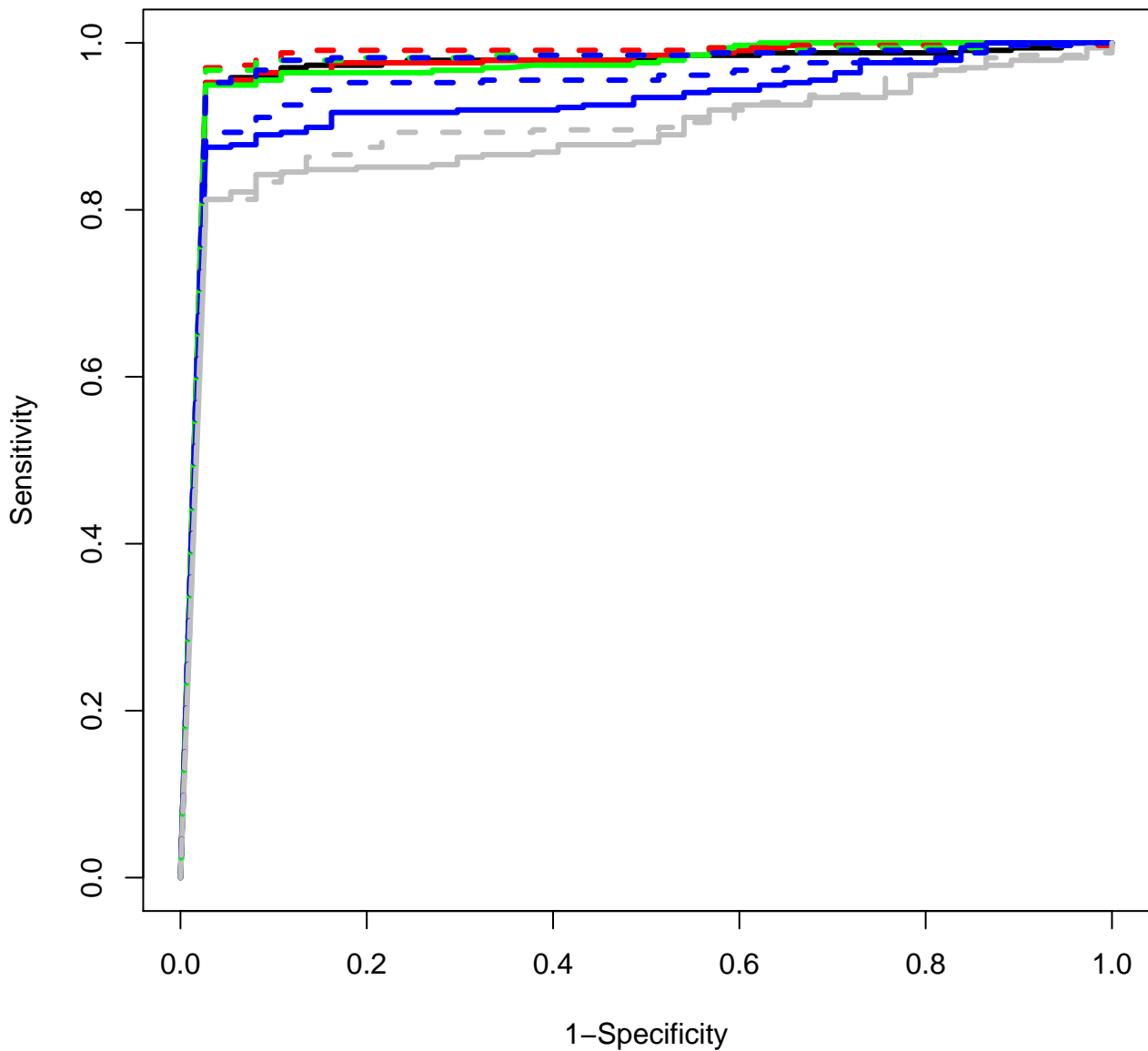

baySeq bk7

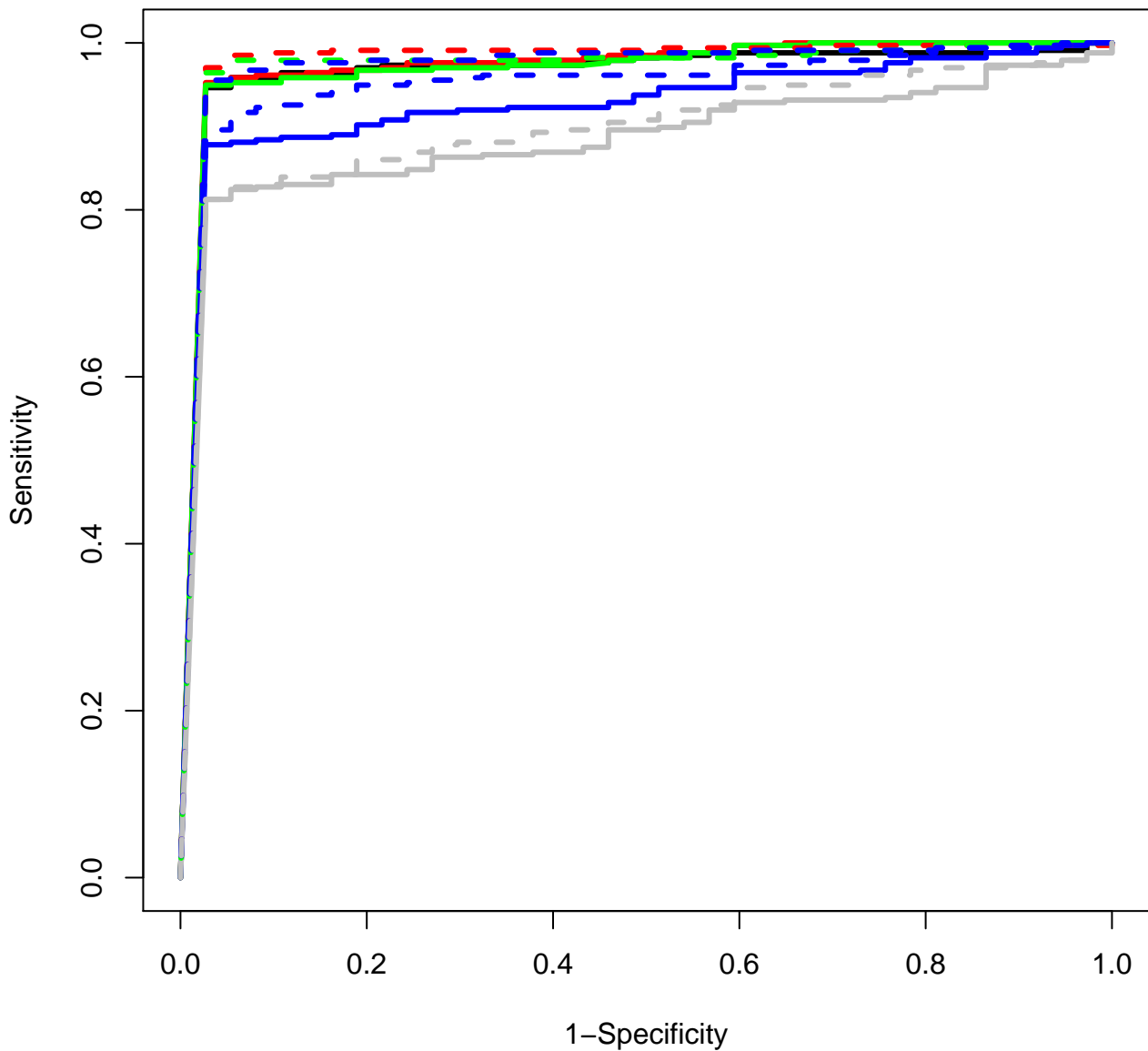

baySeq bk8

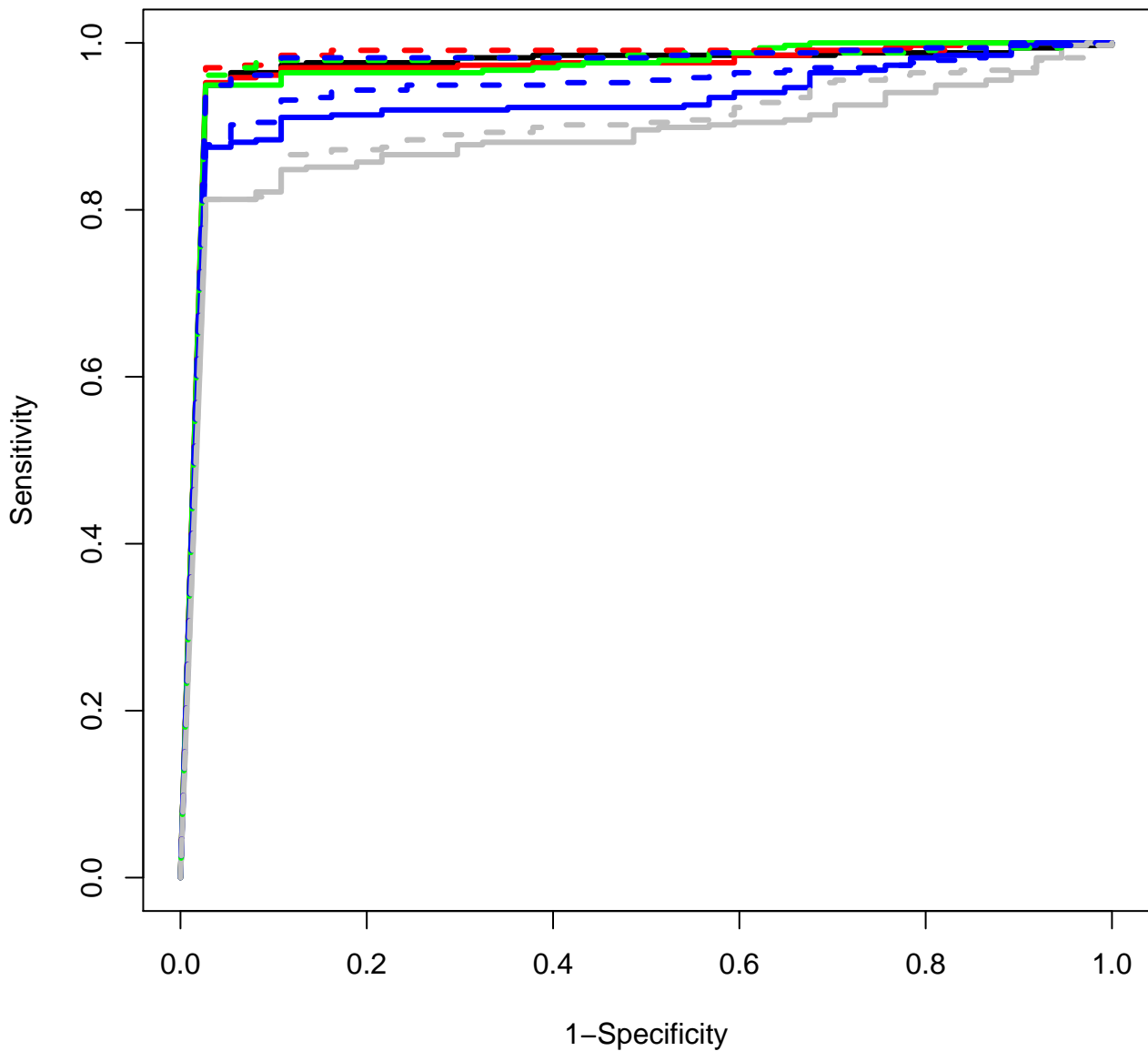

Supplement: Additional Information S4 — ROC curves describing differential expression for baySeq in presence of different backgrounds. (PDF) [file pone.0031630.s004.pdf]

# DESeq bk1

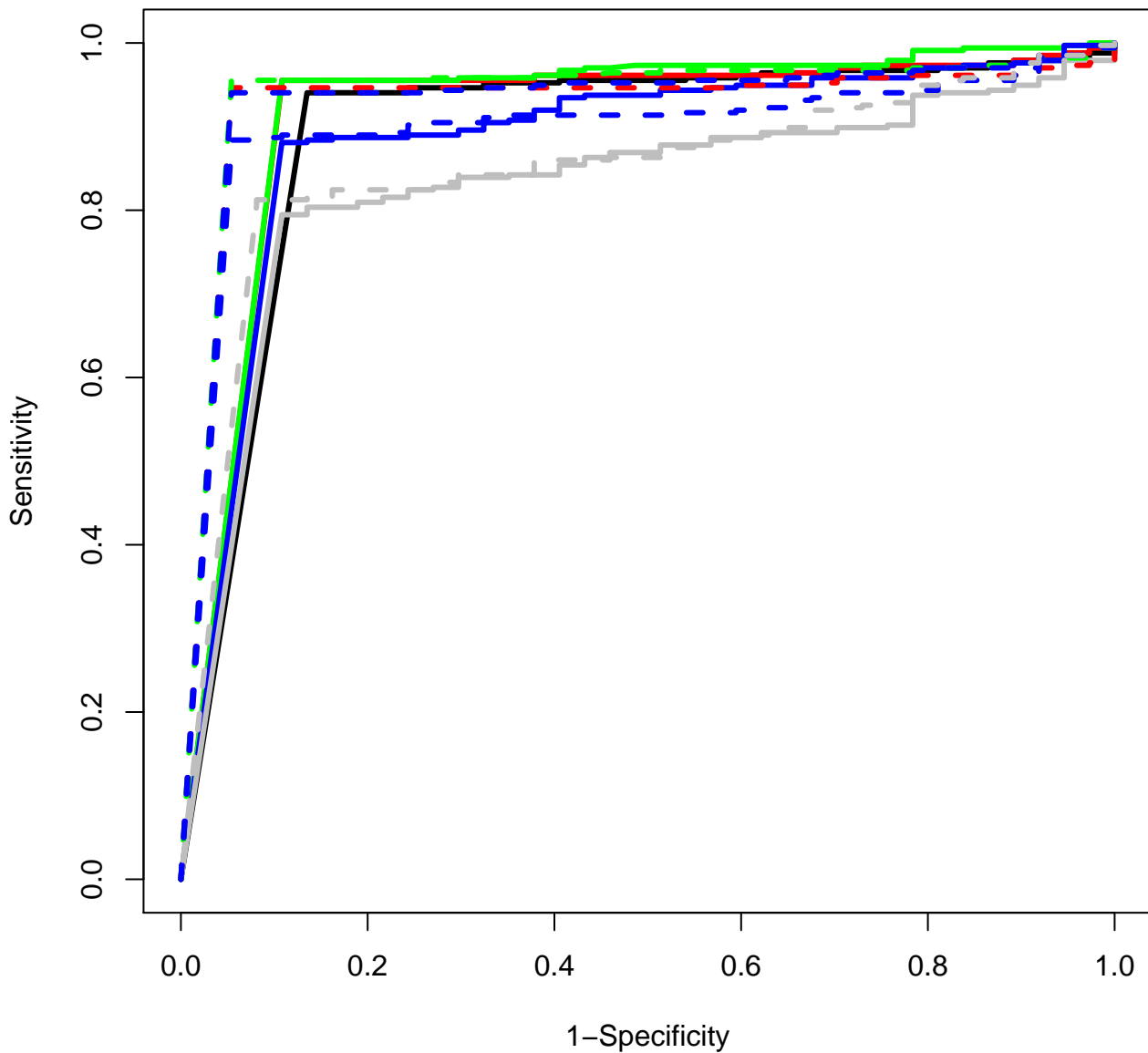

# DESeq bk2

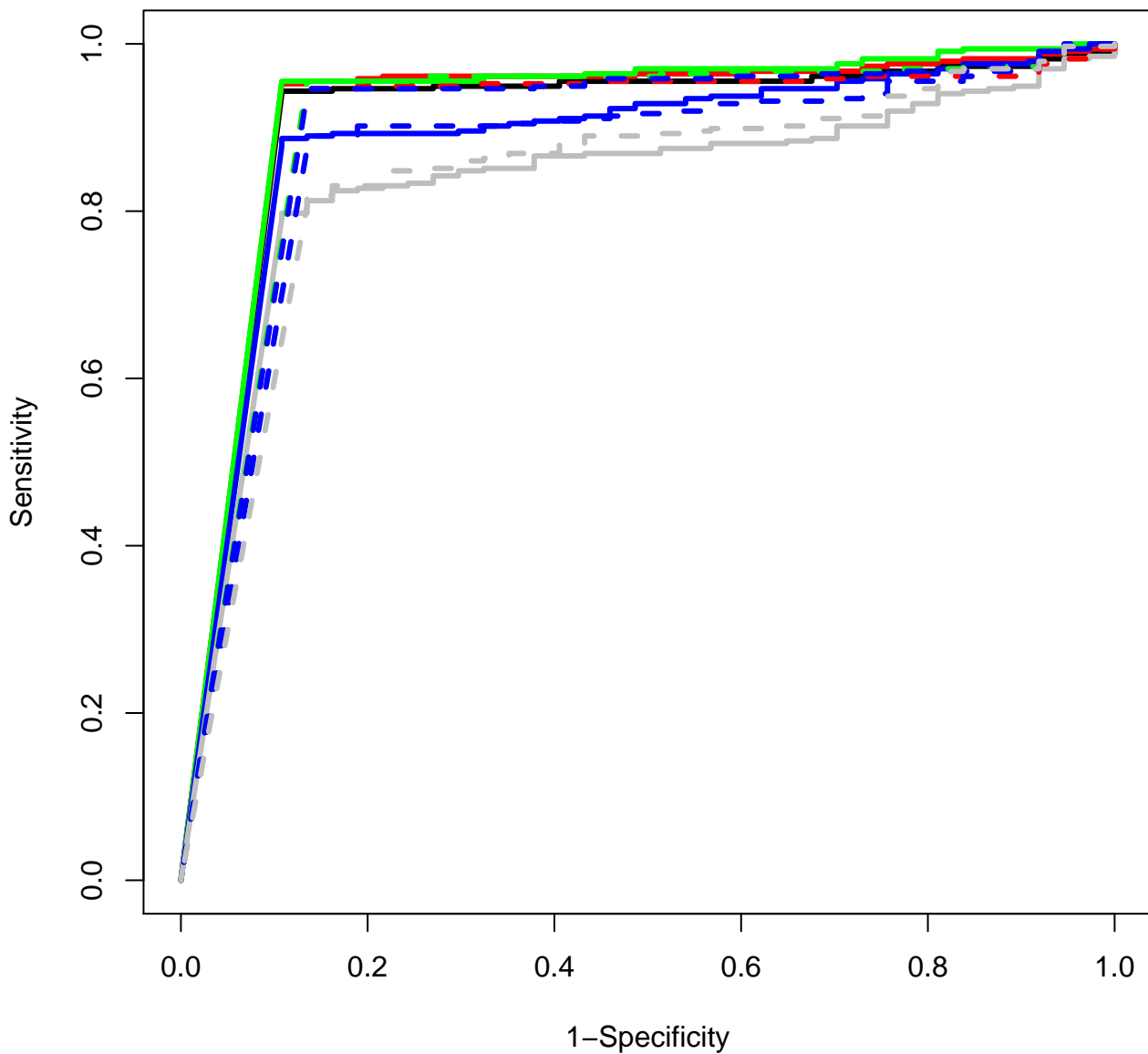

# DESeq bk3

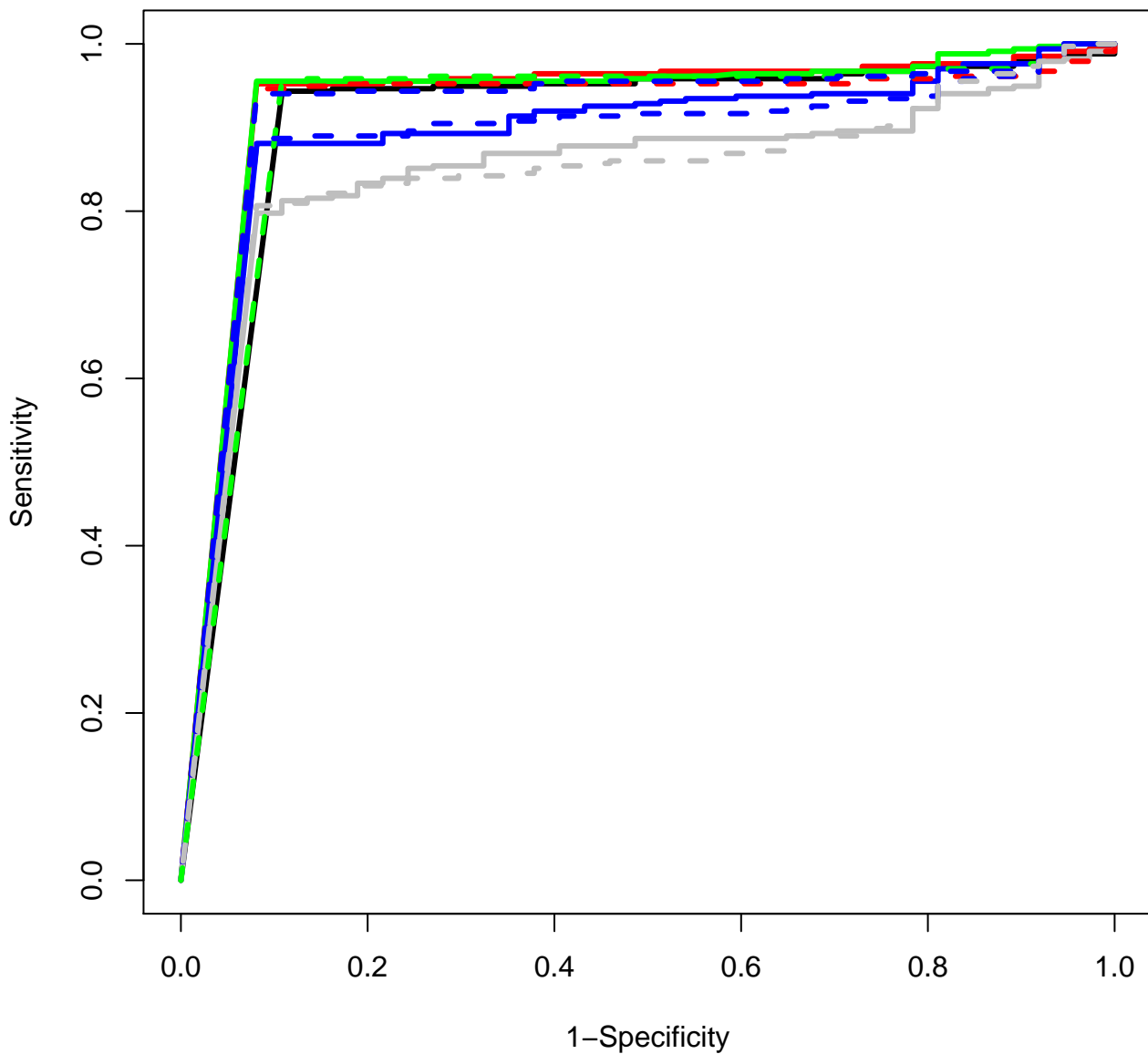

DESeq bk4

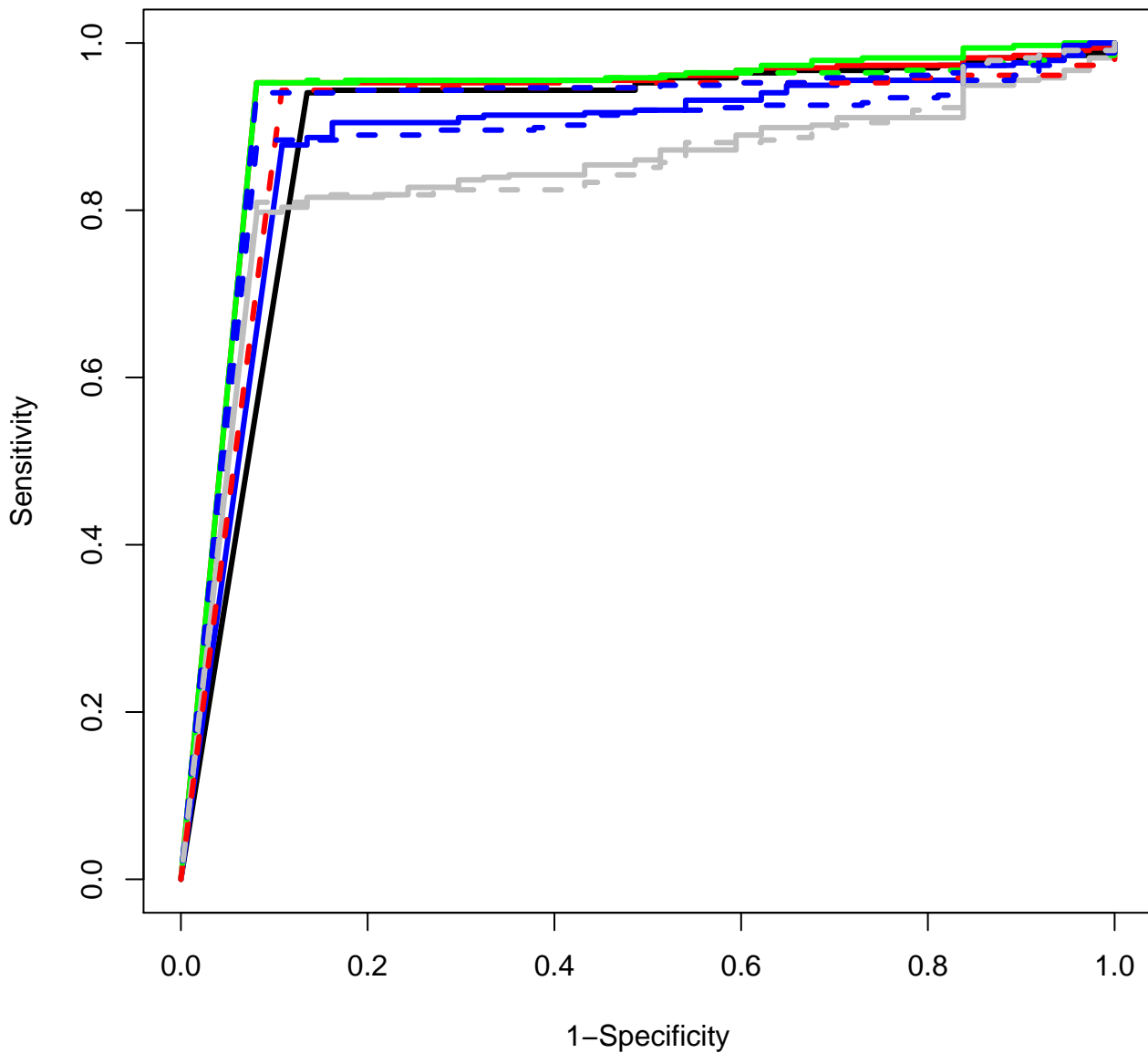

# DESeq bk5

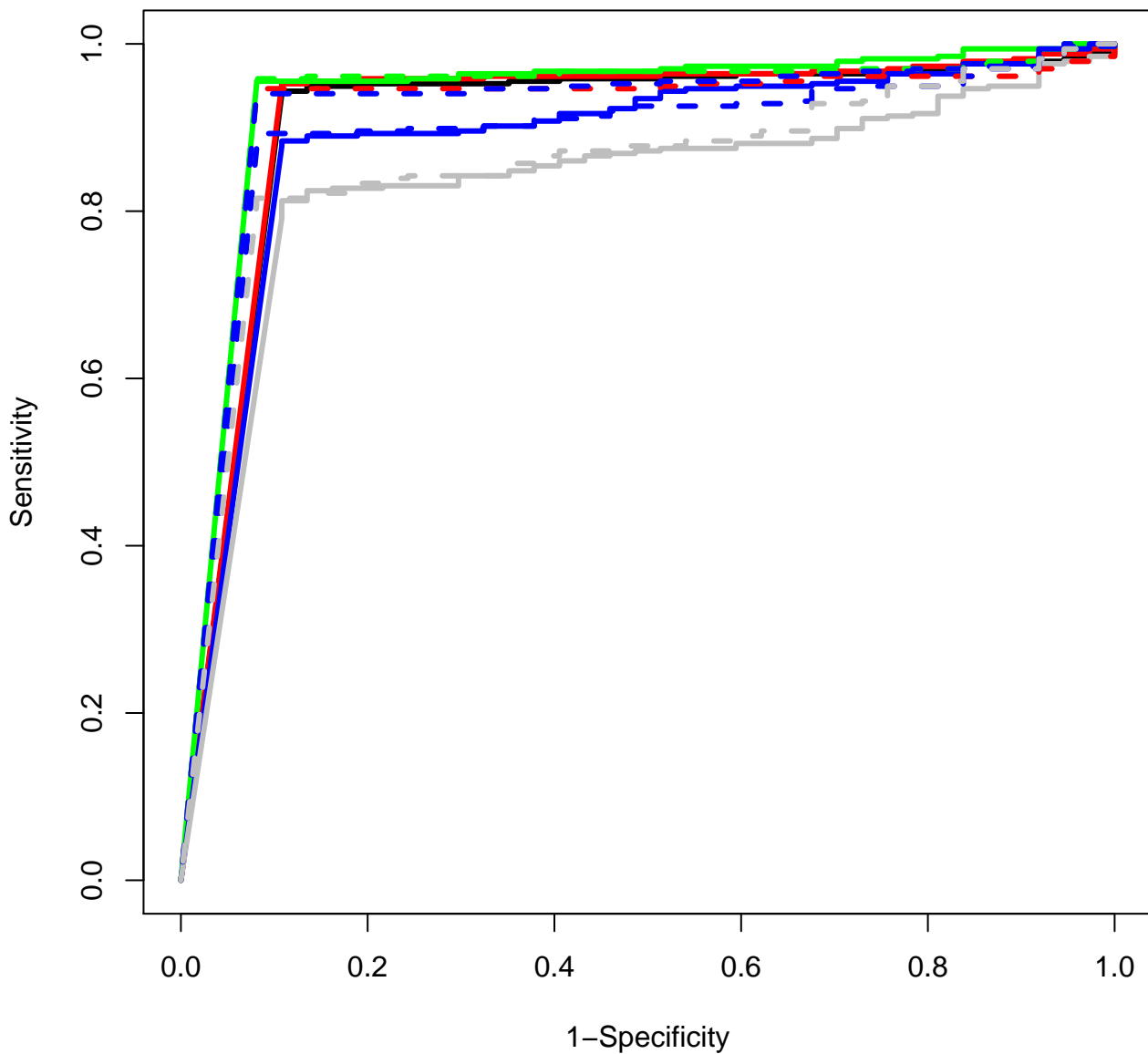

# DESeq bk6

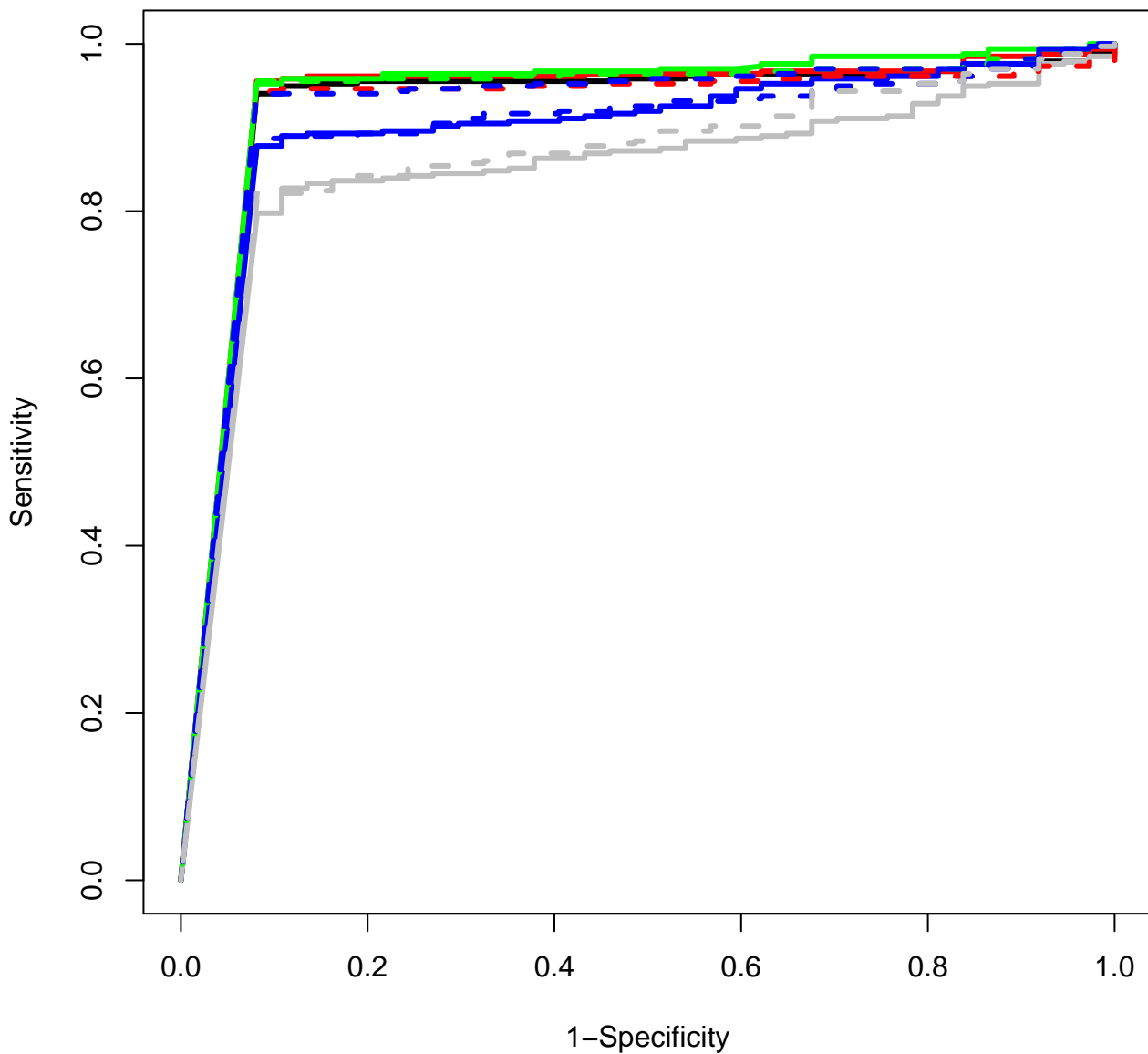

DESeq bk7

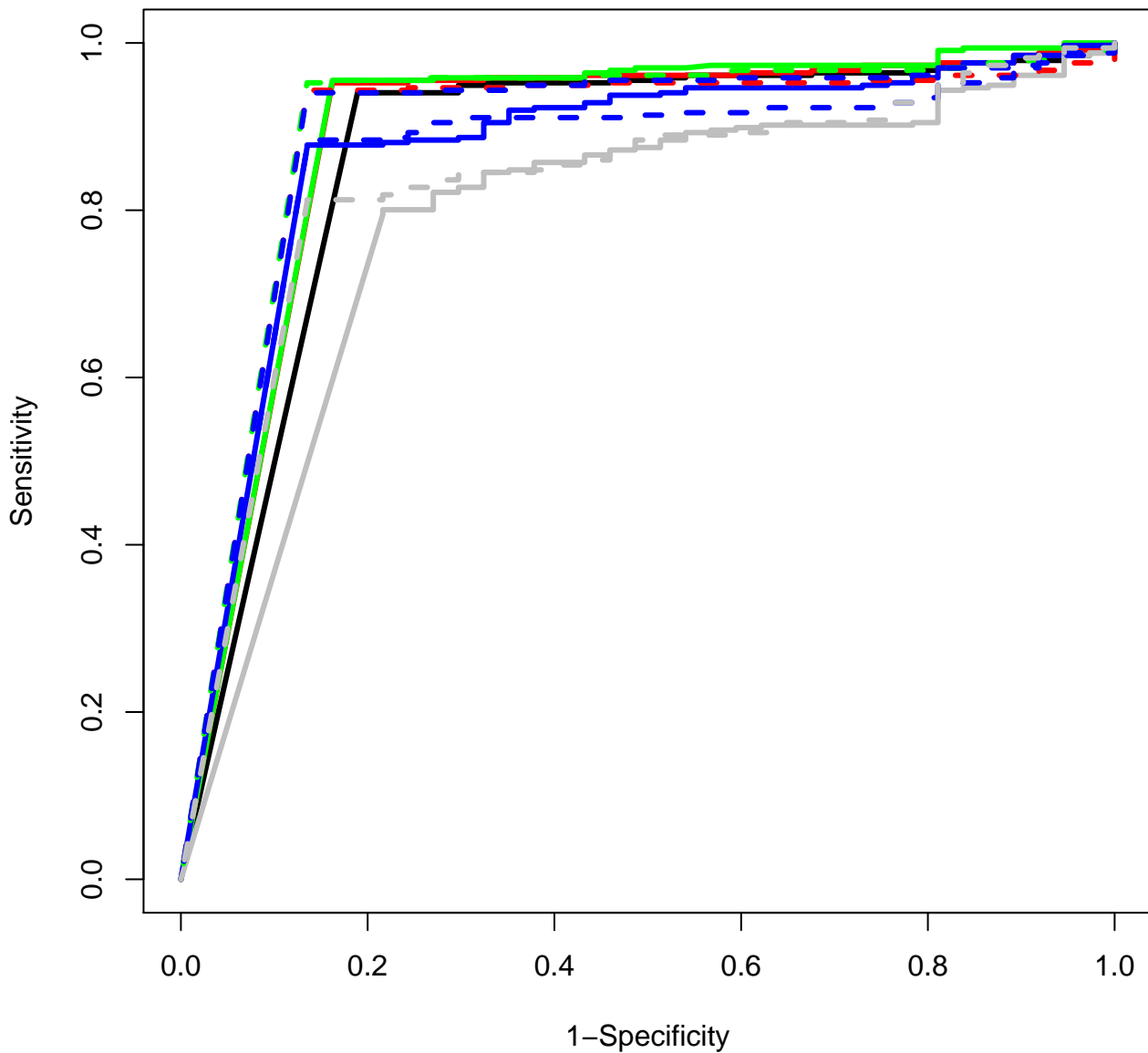

DESeq bk8

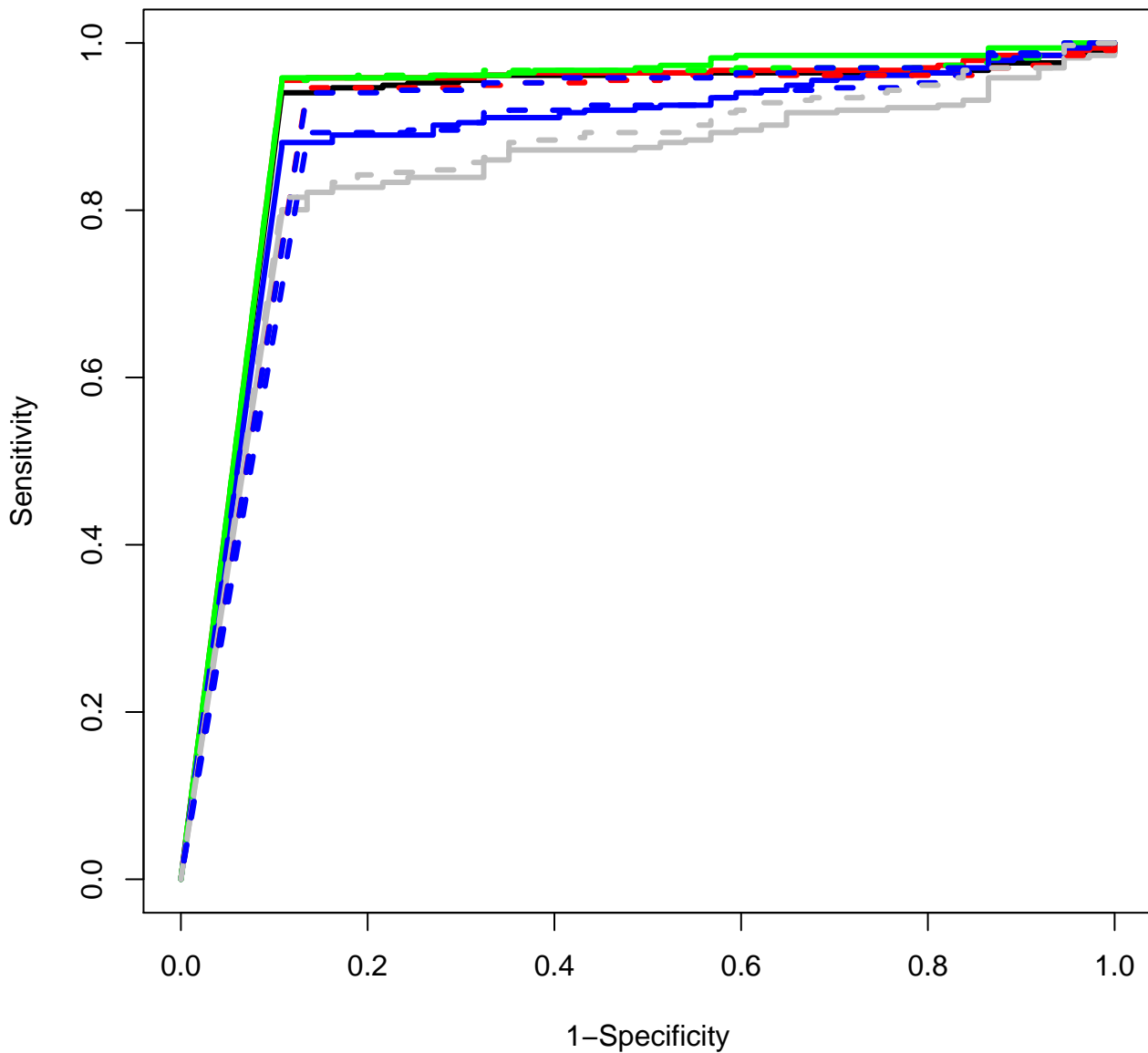

Supplement: Additional Information S5 — ROC curves describing differential expression for DESeq in presence of different backgrounds. (PDF) [file pone.0031630.s005.pdf]
