# Supplementary material for: Optimizing a Massive Parallel Sequencing Workflow for Quantitative miRNA Expression Analysis
Source: PLoS One. 2012 Feb 20;7(2):e31630. doi: 10.1371/journal.pone.0031630 (PMC3282730; doi:10.1371/journal.pone.0031630)
Supplement: Additional Information S6 — ROC curves describing differential expression for rank Product in presence of different backgrounds. (PDF) [file pone.0031630.s006.pdf]

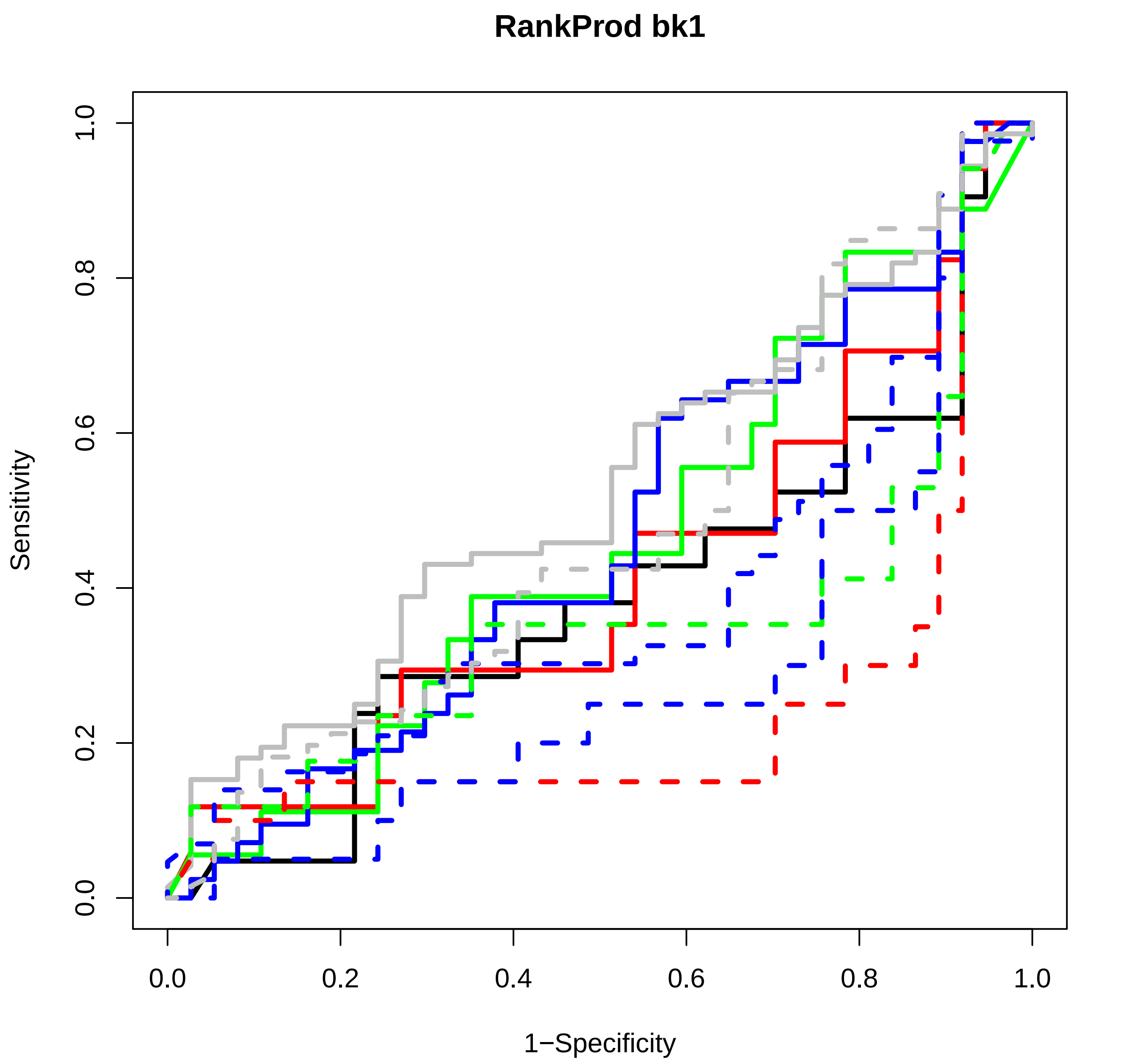

RankProd bk1
Sensitivity
1−Specificity
0.0
0.2
0.4
0.6
0.8
1.0

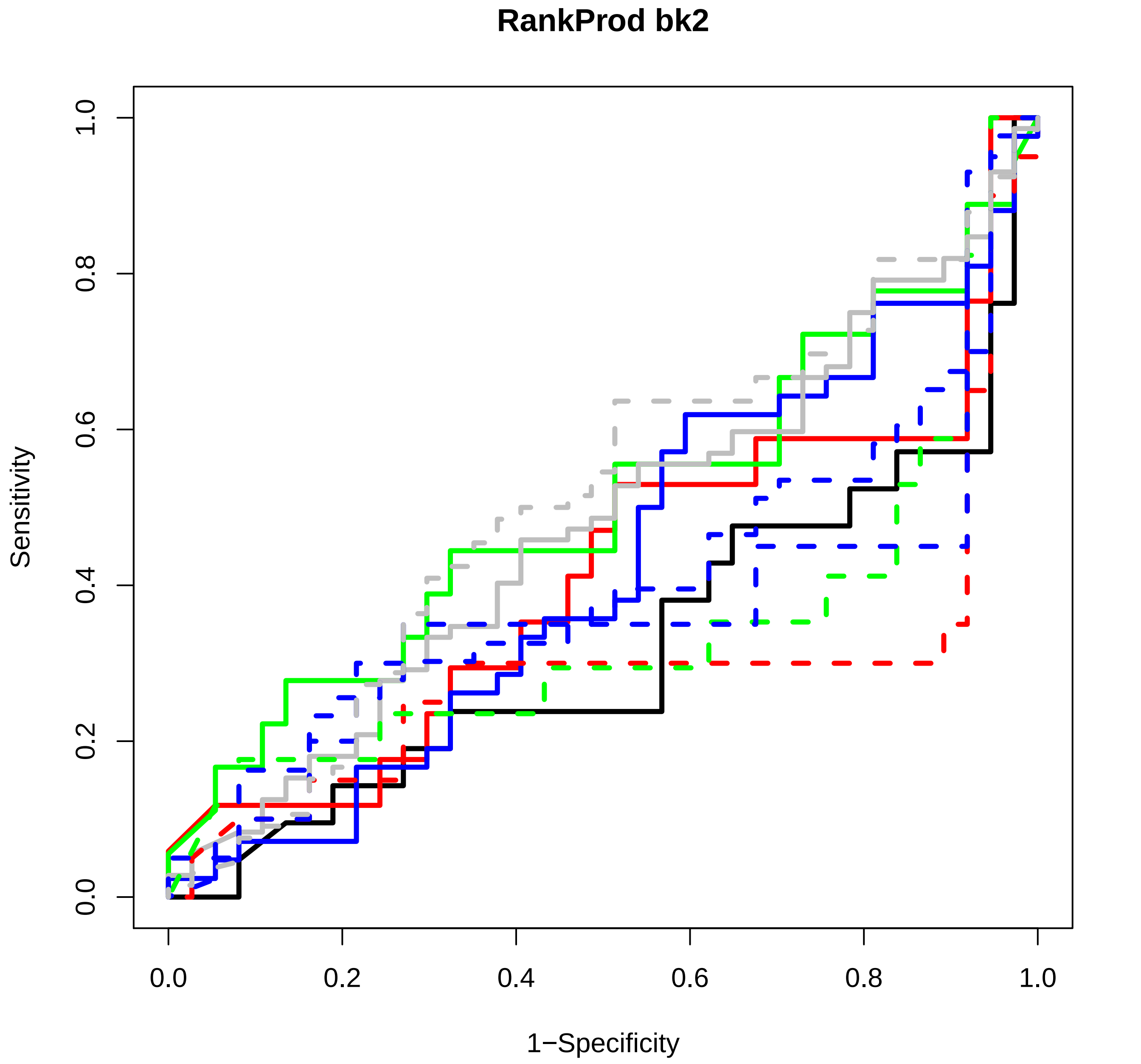
RankProd bk2
Sensitivity
1−Specificity
0.0
0.2
0.4
0.6
0.8
1.0
0.0
0.2
0.4
0.6
0.8
1.0

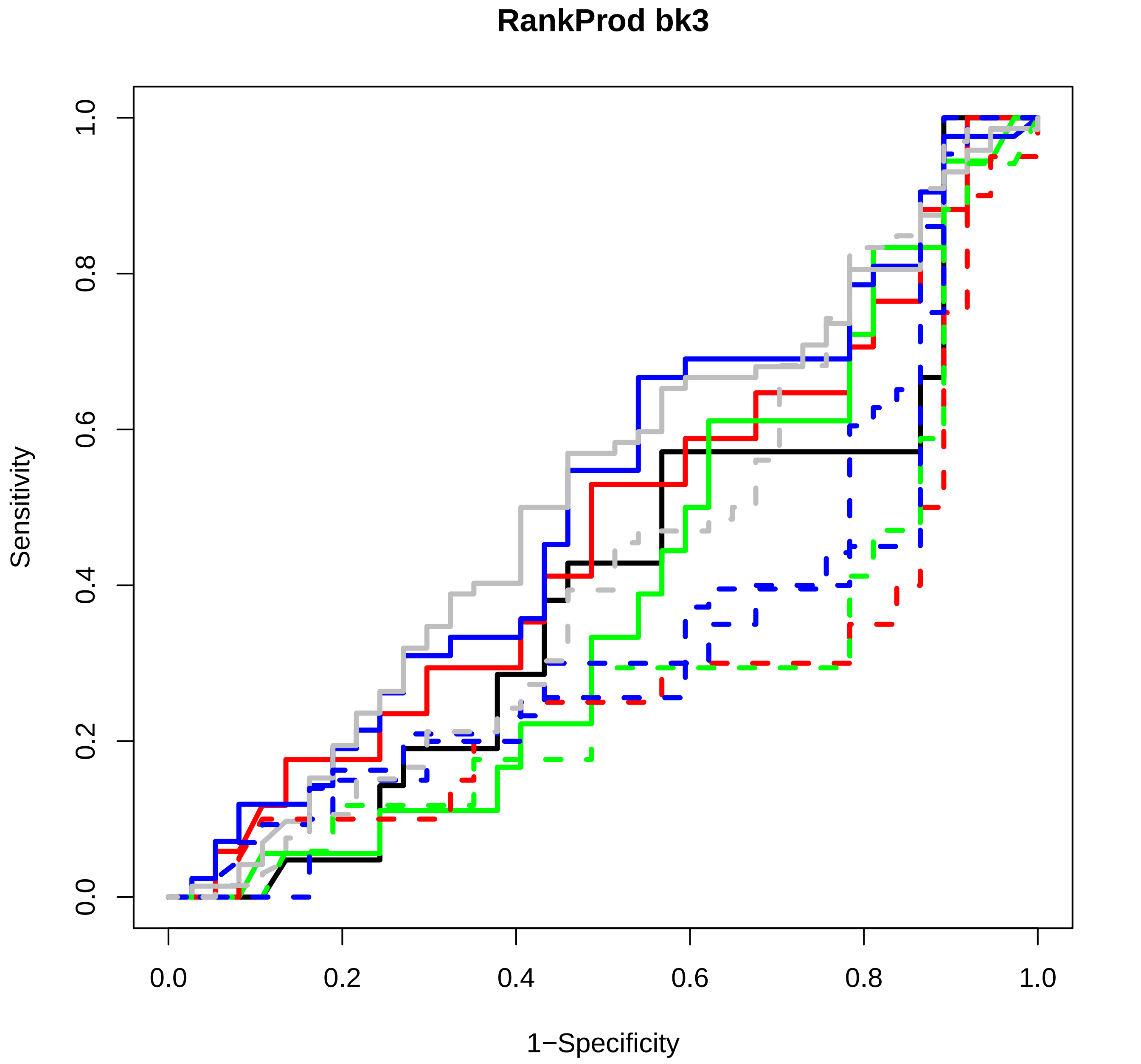
RankProd bk3
Sensitivity
1−Specificity
0.0
0.2
0.4
0.6
0.8
1.0
0.0
0.2
0.4
0.6
0.8
1.0

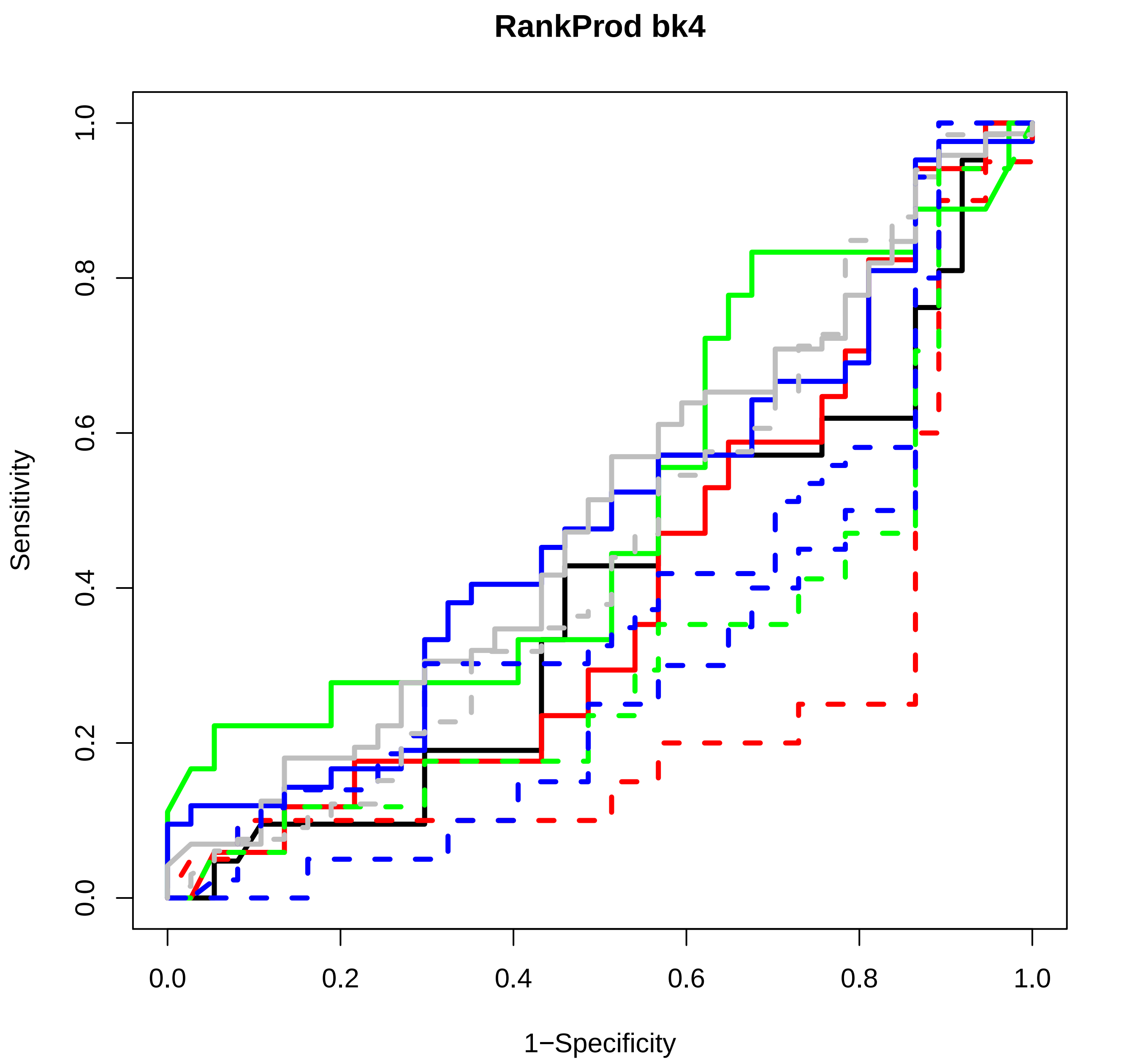
RankProd bk4
Sensitivity
1−Specificity
0.0
0.2
0.4
0.6
0.8
1.0

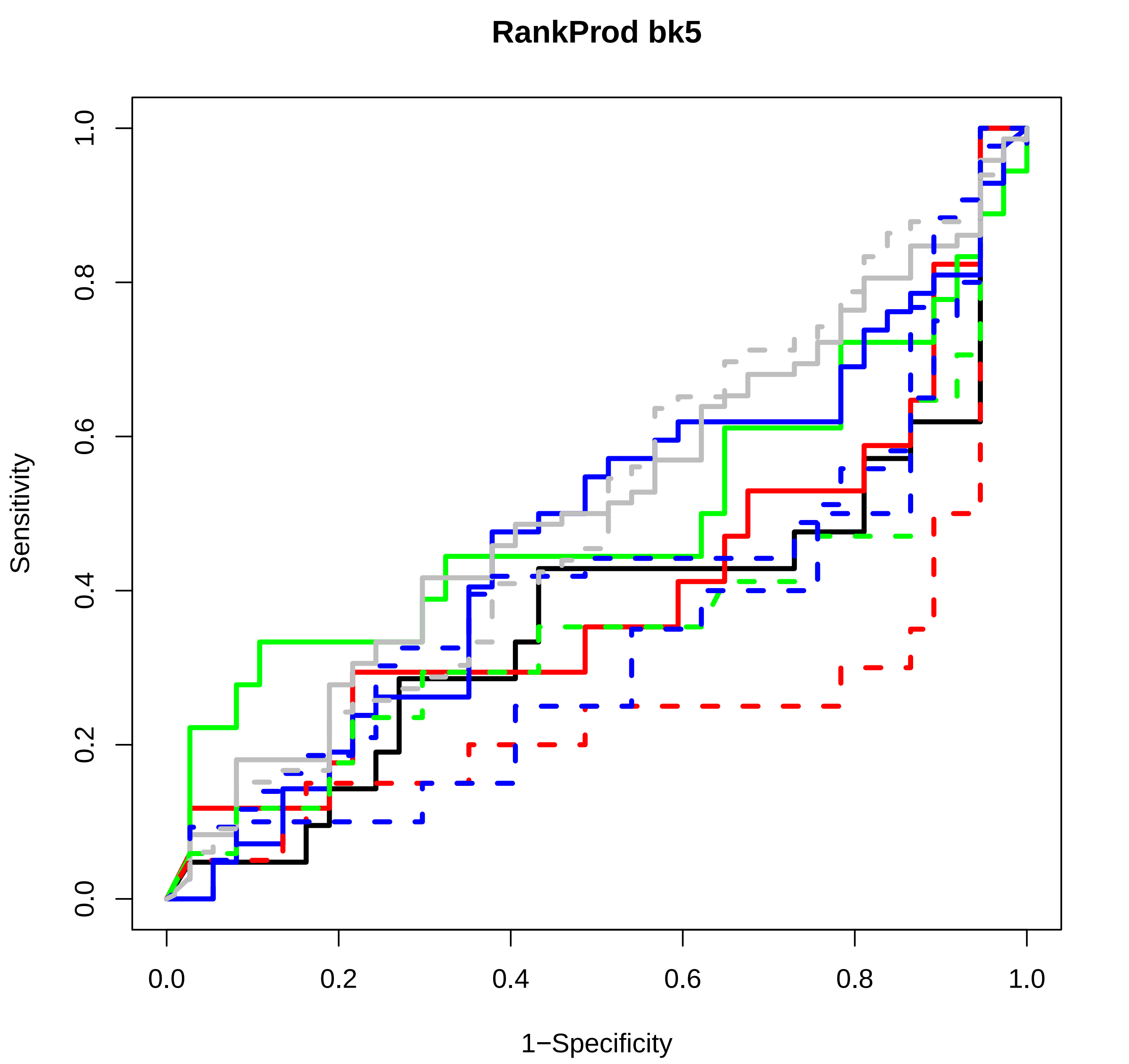

RankProd bk5
Sensitivity
1−Specificity
0.0
0.2
0.4
0.6
0.8
1.0
0.0
0.2
0.4
0.6
0.8
1.0

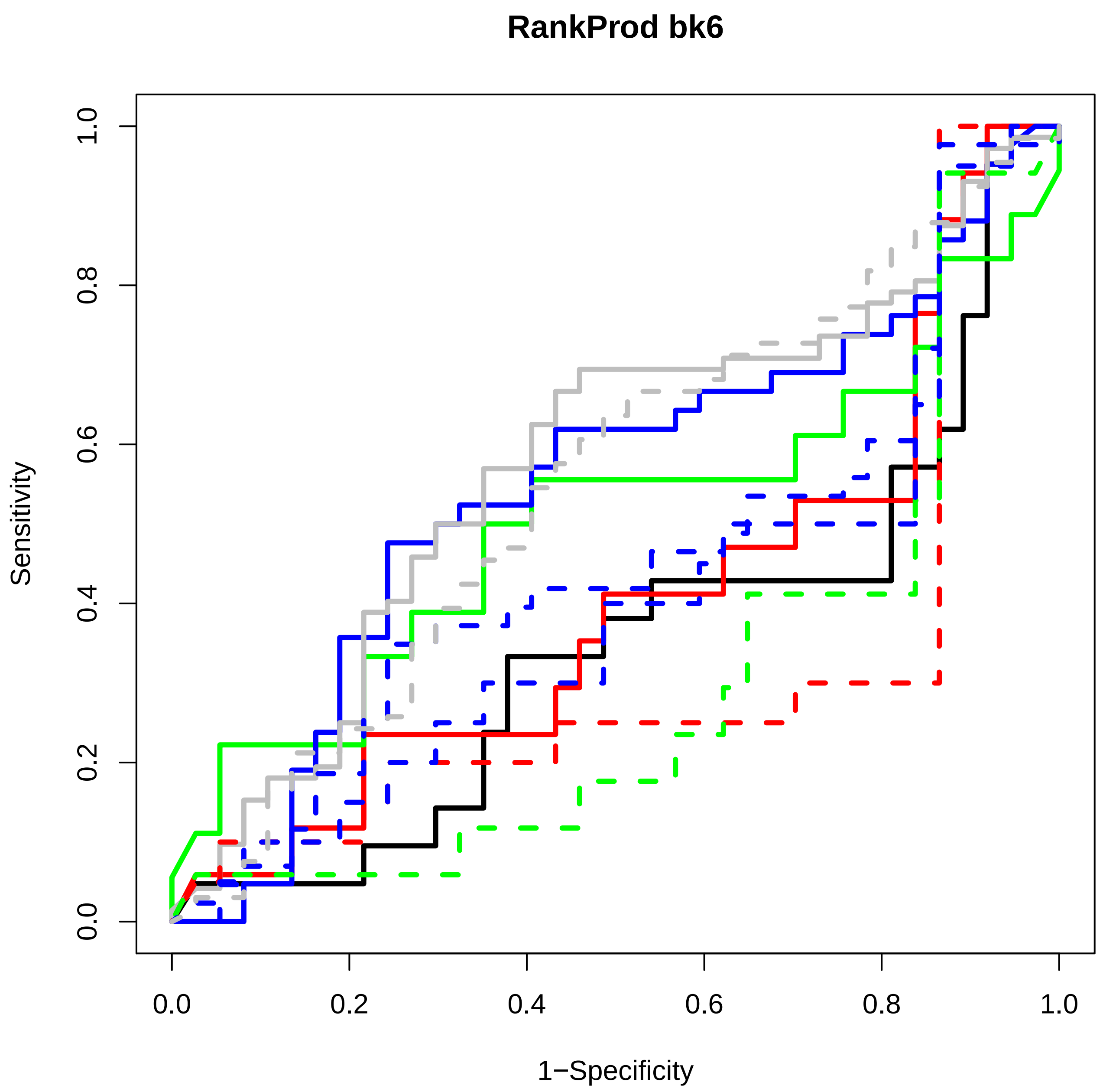

RankProd bk6
Sensitivity
1−Specificity
0.0
0.2
0.4
0.6
0.8
1.0
0.0
0.2
0.4
0.6
0.8
1.0

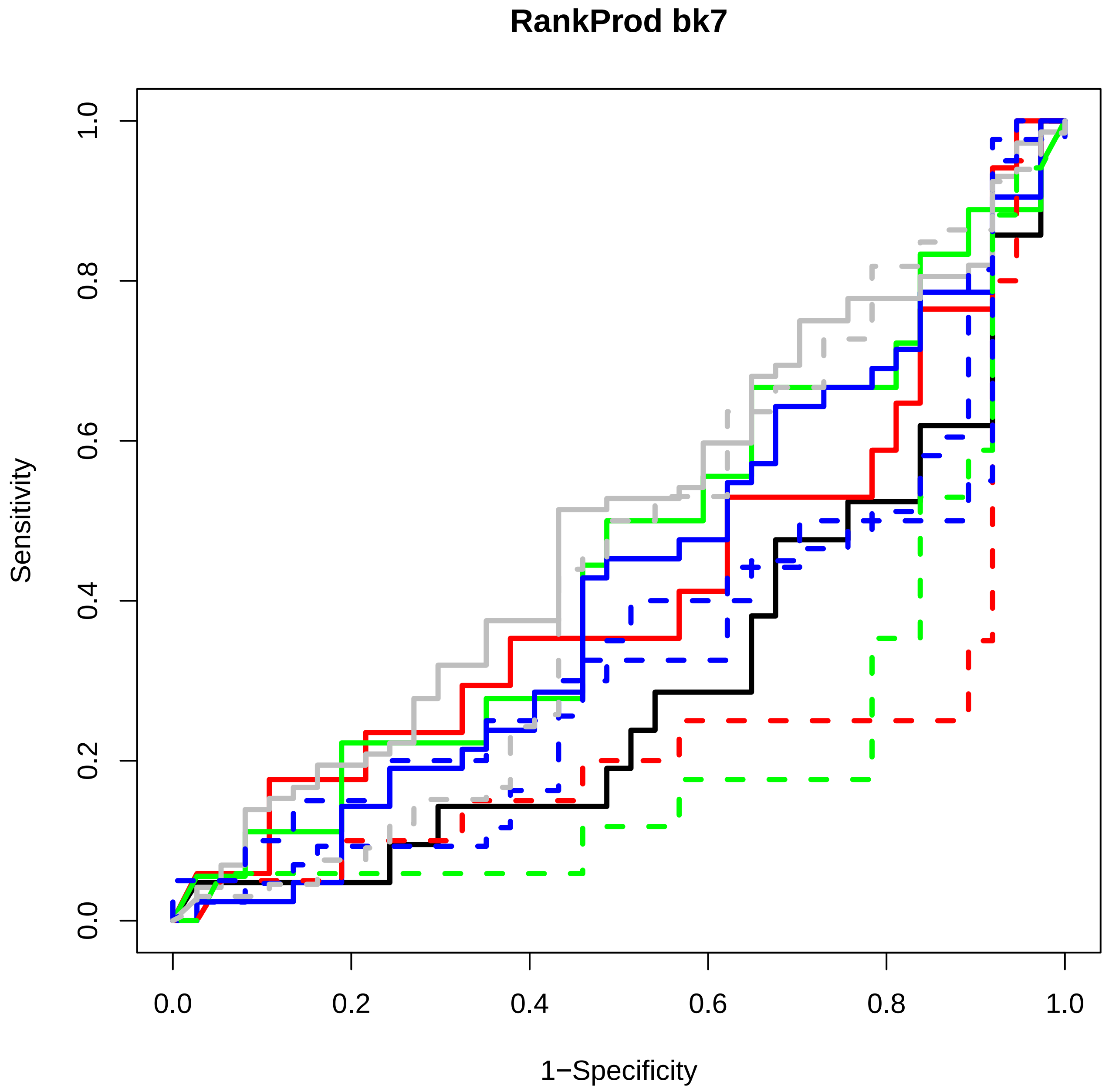

RankProd bk7
Sensitivity
1−Specificity
0.0
0.2
0.4
0.6
0.8
1.0
0.0
0.2
0.4
0.6
0.8
1.0

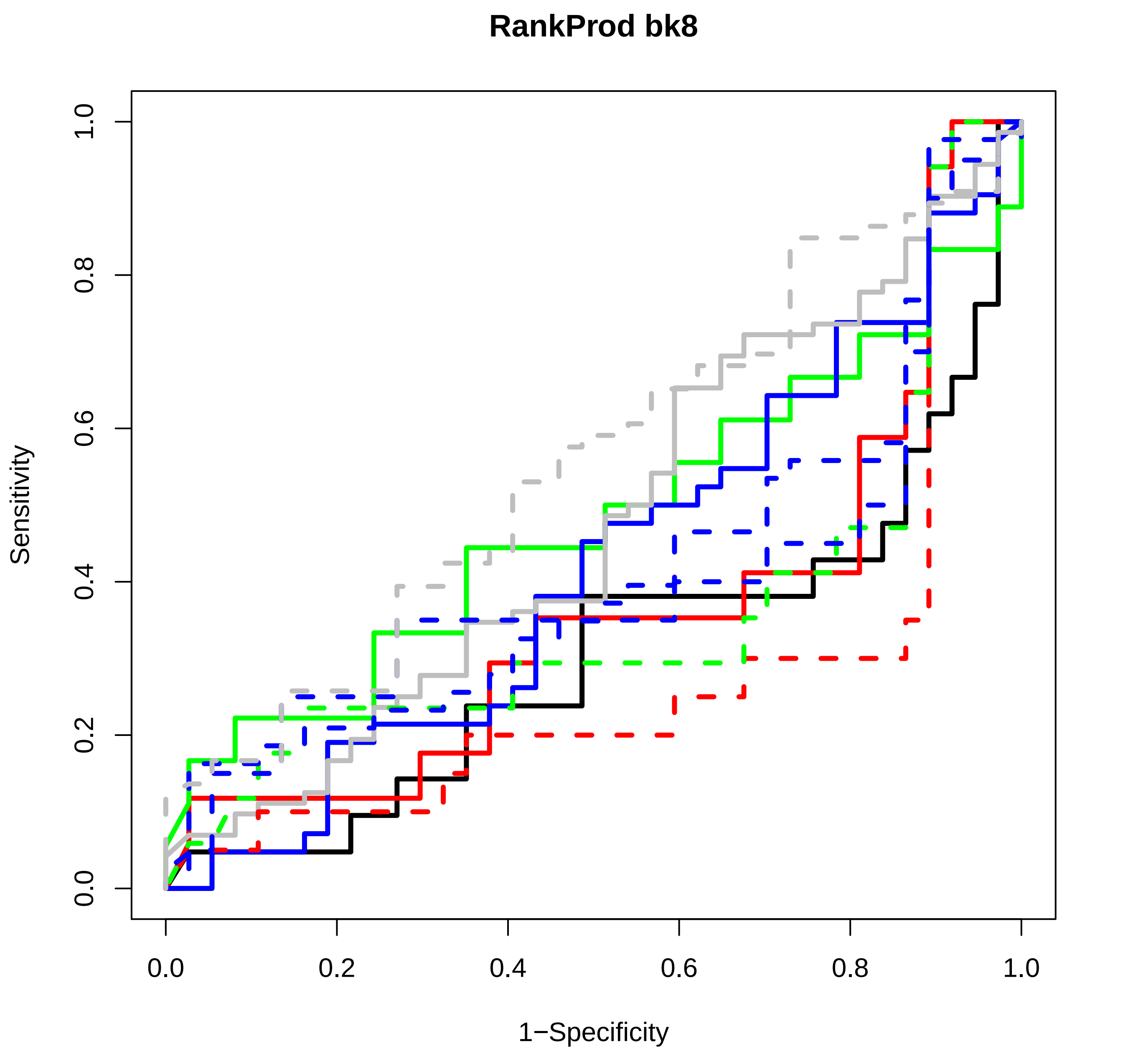
RankProd bk8
Sensitivity
1−Specificity
0.0
0.2
0.4
0.6
0.8
1.0
